# Variation in community and ambulance care processes for out-of-hospital cardiac arrest during the COVID-19 pandemic: a systematic review and meta-analysis

**DOI:** 10.1038/s41598-021-04749-9

**Published:** 2022-01-17

**Authors:** Yoshio Masuda, Seth En Teoh, Jun Wei Yeo, Darren Jun Hao Tan, Daryl Lin Jimian, Shir Lynn Lim, Marcus Eng Hock Ong, Audrey L. Blewer, Andrew Fu Wah Ho

**Affiliations:** 1 Yong Loo Lin School of Medicine, National University of Singapore, Singapore, Singapore; 2 Department of Cardiology, National University Heart Center, Singapore, Singapore; 3 Department of Emergency Medicine, Singapore General Hospital, C/O Office C, 1 Outram Rd, Singapore, 169608 Singapore; 4 Health Services & Systems Research, Duke-NUS Medical School, Singapore, Singapore; 5 Department of Family Medicine and Community Health and Department of Population Health Sciences, Duke University, Durham, NC USA; 6 Pre-Hospital and Emergency Research Centre, Duke-NUS Medical School, Singapore, Singapore

**Keywords:** Cardiology, Health policy

## Abstract

Bystander cardiopulmonary resuscitation (BCPR), early defibrillation and timely treatment by emergency medical services (EMS) can double the chance of survival from out-of-hospital sudden cardiac arrest (OHCA). We investigated the effect of the COVID-19 pandemic on the pre-hospital chain of survival. We searched five bibliographical databases for articles that compared prehospital OHCA care processes during and before the COVID-19 pandemic. Random effects meta-analyses were conducted, and meta-regression with mixed-effect models and subgroup analyses were conducted where appropriate. The search yielded 966 articles; 20 articles were included in our analysis. OHCA at home was more common during the pandemic (OR 1.38, 95% CI 1.11–1.71, p = 0.0069). BCPR did not differ during and before the COVID-19 pandemic (OR 0.94, 95% CI 0.80–1.11, p = 0.4631), although bystander defibrillation was significantly lower during the COVID-19 pandemic (OR 0.65, 95% CI 0.48–0.88, p = 0.0107). EMS call-to-arrival time was significantly higher during the COVID-19 pandemic (SMD 0.27, 95% CI 0.13–0.40, p = 0.0006). Resuscitation duration did not differ significantly between pandemic and pre-pandemic timeframes. The COVID-19 pandemic significantly affected prehospital processes for OHCA. These findings may inform future interventions, particularly to consider interventions to increase BCPR and improve the pre-hospital chain of survival.

## Introduction

Out-of-hospital cardiac arrest (OHCA) is a time-critical medical emergency, in which clinical outcomes are thoroughly dependent on a well-organized “chain of survival”^1,2^. This prehospital component of the “chain of survival” involves timely and seamless bystander cardiopulmonary resuscitation (BCPR), the use of automated external defibrillators (AED), as well as treatment by emergency medical services (EMS). However, the unprecedented coronavirus disease 2019 (COVID-19) pandemic had a poorly understood impact on EMS resources and was believed to have disrupted the prehospital “chain of survival” particularly layperson or bystander response^3,4^. There is tremendous scientific and public health interest in how community and EMS-related processes were altered since these confer larger survival impact relative to advanced hospital-based interventions, and the benefits of the latter are confined to those who had received timely prehospital interventions^5^. Understanding the impact of the COVID-19 pandemic on OHCA care processes is key to planning of future public health programs and policies to improve OHCA outcomes in the post-pandemic era and pandemic-preparedness.

Studies that assessed the impact of the pandemic on the prehospital “chain of survival”, particularly early bystander response, have reported inconsistent findings^6,7^. Marijon et al. reported a decrease in BCPR possibly because bystanders were more hesitant to perform CPR on possible COVID-19 cases^8^. This was particularly worrying since BCPR with early defibrillation may double a victim’s chances of survival^9^. This observation, however, was not found in some other studies^10,11^, prompting a review of the current literature on BCPR before and during the pandemic and on related variables such as OHCA at home, witnessed OHCA, and bystander AED use. EMS care processes in the pre-hospital “chain of survival” are also key factors contributing to improved outcomes in OHCA, including EMS resuscitation attempts and duration, EMS call to arrival times, and the use of various advanced life support measures such as endotracheal intubation, supraglottic airway devices, amiodarone, epinephrine, and mechanical CPR. These factors are critical in the pre-hospital management of OHCA patients before accessing advanced care^12^. Previously, Lim et al. suggested that EMS call to arrival times during the pandemic may have risen due to challenges such as increased personal protective equipment (PPE) requirements^13^. However, the impact of the pandemic on EMS care processes is unclear.

Therefore, this systematic review and meta-analysis aimed to investigate the effect of the COVID-19 pandemic on OHCA care processes and clarify the role of the pre-hospital “chain of survival” during the pandemic. While there exist previous reviews on similar topics, none have specifically focused and comprehensively reviewed the impact of COVID-19 on the pre-hospital “chain of survival”^3,14^. We hypothesized that OHCA increased in the home and BCPR rates decreased during the pandemic. Furthermore, we postulated that EMS call to arrival times increased during the pandemic.

## Methods

This systematic review and meta-analysis adhered to the Preferred Reporting Items for Systematic Reviews and Meta-Analyses (PRISMA) guidelines^15^. It is registered in the International Prospective Register of Systematic Reviews (PROSPERO) (CRD42021274223)^16^.

### Search strategy

The search strategy was developed in consultation with a medical information specialist at NUS, Singapore. We utilized the Medical Subject Headings (MeSH) term “heart arrest” and the non-MeSH terms “sudden cardiac arrest, sudden cardiac death, out of hospital cardiac arrest (OOHCA), out-of-hospital cardiac arrest, cardiac arrest, OHCA, OOHCA, COVID-19, Coronavirus, severe acute respiratory syndrome coronavirus 2 (SARS-CoV-2)”. An exhaustive literature search was performed in five bibliographic databases from the date of the first reported COVID-19 case (December 31, 2019) to May 3rd, 2021: PubMed, EMBASE, Web of Science, Scopus and The Cochrane Central Register of Controlled Trials (CENTRAL) in The Cochrane Library. References of relevant articles were hand-searched to identify additional relevant studies. The search strategy is available in the Appendix I.

### Selection criteria

The inclusion criteria were: (A) patients with OHCA during the COVID-19 pandemic; (B) articles that reported any of the following characteristics and outcomes: OHCA at home, unwitnessed OHCA, BCPR, AED use, EMS resuscitation attempted, resuscitation duration, EMS call to arrival time, use of endotracheal intubation, use of supraglottic airway, use of amiodarone, use of epinephrine, use of mechanical CPR; and (C) articles that compared the above mentioned outcomes during and before the pandemic. We ensured that there was no overlapping or repeated data from the included studies.

The exclusion criteria were: (A) all articles not written in the English language; (B) all articles that did not utilize a historical control (comparing outcomes during and before the pandemic); (C) case reports; (D) case series with fewer than five patients; and (E) conference abstracts and posters.

The web-based platform Rayyan QCRI was utilized to perform article deduplication, screening and assessment for final eligibility^17^. Two authors (Y.M and S.E.T) performed the literature search and evaluated the eligibility of studies independently. Disagreements were resolved after consensus with the senior author, A.F.W.H.

### Data extraction and quality assessment

Three authors (Y.M, S.E.T, D.J.H.T) independently extracted data from included studies to a spreadsheet. Any conflicts with data collection were arbitrated after consensus with a senior author, A.F.W.H. We extracted the following data—(A) study characteristics including first author details, year of publication, study origin, study design and population, time periods and sample sizes of (i) COVID-19 pandemic (ii) Pre-COVID-19 pandemic; (B) patient characteristics including age and gender; (C) community processes-related outcomes such as OHCA incidence at home, unwitnessed OHCA events, BCPR, and AED use; and (D) EMS processes-related outcomes such as EMS resuscitation attempted, resuscitation duration, EMS call to arrival time, endotracheal intubation and supraglottic airway, amiodarone and epinephrine, and use of mechanical CPR. If the data presented were missing or unclear, we contacted the corresponding author by email for clarification.

The methodological quality of the included studies was assessed by two authors (Y.M and S.E.T) independently using the Newcastle–Ottawa Scale (NOS). The scale included eight items, and possible scores ranged from zero to nine. Studies with a score of seven or more were considered high quality.

### Statistical analysis

Meta-analyses were conducted for the community processes (OHCA location at home, unwitnessed OHCA, BCPR and AED use), as well as EMS processes of OHCA patients (EMS resuscitation attempted, resuscitation duration, EMS call to arrival time, endotracheal intubation and supraglottic airway, amiodarone and epinephrine, and mechanical CPR).

Data analyses were performed using the *meta 4.18–0* and *metafor 2.4–0* packages with R 3.6.3 (R Foundation for Statistical Computing, Vienna, Austria). Random-effects models were used in conjunction with the Sidik–Jonkman estimator and Mantel–Haenszel method to estimate the pooled effects of COVID-19, as substantial between-study heterogeneity was present. Forest plots displayed individual and pooled odds ratios (OR) and 95% confidence intervals (95% CI) for the binary outcomes: OHCA at home, unwitnessed OHCA, BCPR, AED use, EMS resuscitation attempted, endotracheal intubation and supraglottic airway, amiodarone and epinephrine, and mechanical CPR. For the continuous outcomes (resuscitation duration and EMS call to arrival time), forest plots displayed individual and pooled standardized mean difference (SMD) and 95% CI. Two-tailed statistical significance was set at p-value ≤ 0.05. The *I*^2^ statistic was used to quantify statistical heterogeneity^18^. This statistic indicates whether variation is more likely due to chance or study heterogeneity, with *I*^2^ values of 25%, 50%, and 75% indicating low, moderate, and high heterogeneity respectively. Whenever there was substantial statistical heterogeneity (*I*^2^ > 50%), we evaluated for outliers by performing a set of case deletion diagnostics to identify influential studies and subsequent leave-one-out sensitivity analyses. To account for possible moderators that might contribute to statistical heterogeneity, we performed univariate meta-regression with mixed-effects models and subgroup analyses for the outcome of BCPR. Publication bias was evaluated via visual evaluation of funnel plots and Egger’s regression.

## Results

### Literature retrieval

**Figure 1:**
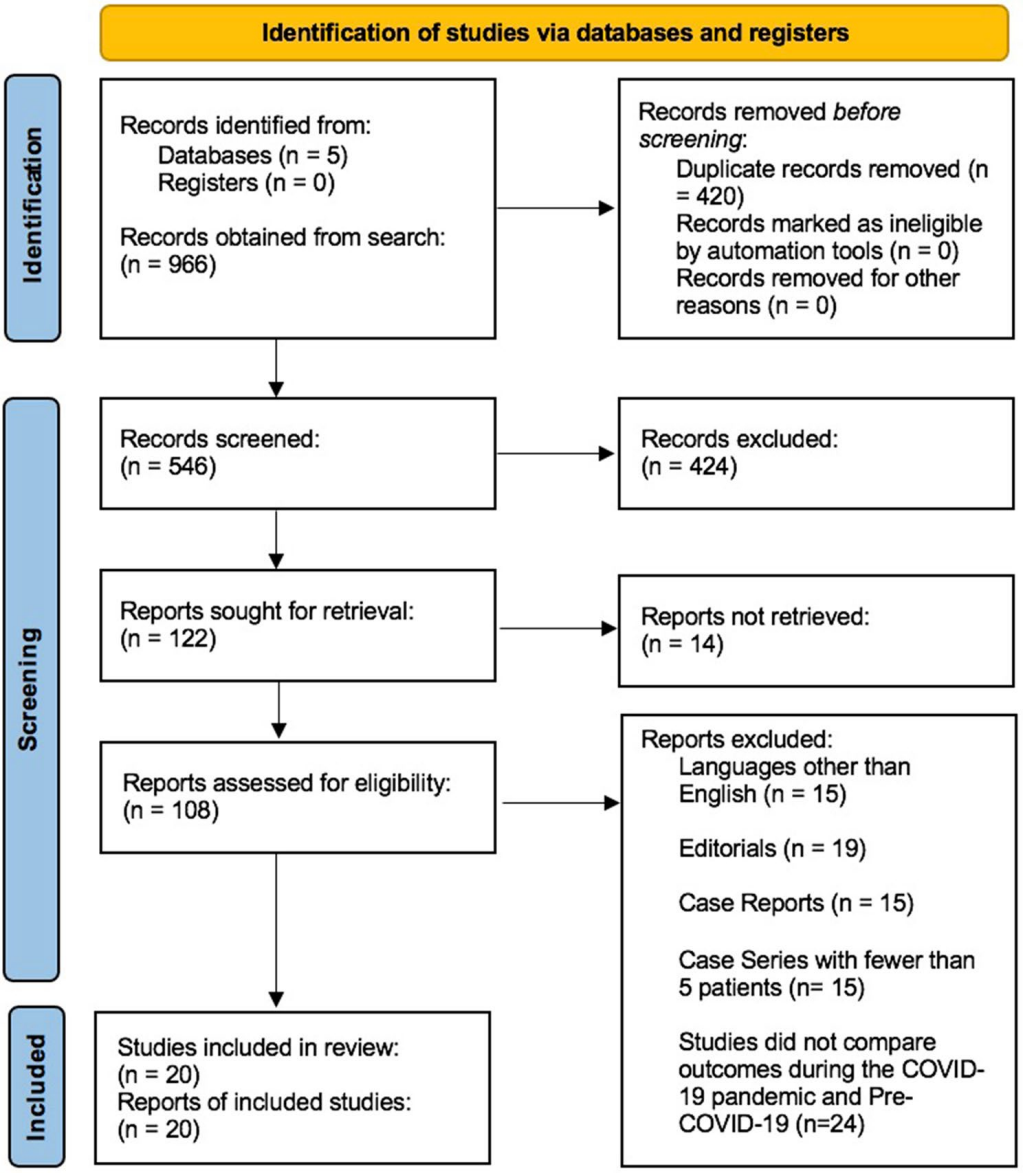
PRISMA-P flowchart for study selection. From: Page et al.^15^.

The database search yielded a total of 966 articles. After removal of duplicates, 546 abstracts were screened and subsequently 122 reports were sought for retrieval, of which 14 articles could not be retrieved. The resultant 108 full-texts were reviewed, and 20 were identified as meeting the selection criteria. The study selection process and reasons for exclusion were illustrated in the PRISMA-P 2020 Flow Diagram (Fig. 1).

### Characteristics of studies and risk of bias

The 20 included studies originated from ten countries (France, Italy, Australia, Korea, United States of America, Spain, Netherlands, United Kingdom, Singapore, and Sweden). All studies included were retrospective cross-sectional study design.

There were a total of 67,815 patients with OHCA across the studies, of which 28,960 patients were evaluated during the COVID-19 pandemic and 38,855 patients were evaluated prior to the COVID-19 pandemic. Study sample sizes ranged from 101 to 19,303 patients. The study characteristics were summarized in Table 1.

**Table 1:** Characteristics of included studies.

Study	Location	Study design^#^	Study population	Time period (i) COVID-19 pandemic (ii) Pre-COVID-19 pandemic	Sample size (i) COVID-19 pandemic (ii) Pre-COVID-19 pandemic	Age (years), Mean (SD) (i) COVID-19 pandemic (ii) Pre-COVID-19 pandemic	Male Gender, N (%) (i) COVID-19 pandemic (ii) Pre-COVID-19 pandemic
Baert et al., 2020^19^	France	Registry-based study	Adult and pediatric cases of presumed medical etiology (EMS-treated NR; Received resuscitation NR)	(i) March 1–April 31, 2020 (ii) March 1–April 31, 2019	(i) 1005 (ii) 1620	(i) 68.0 (17.0) (ii) 69.0 (17.0)	(i) 676/1005 (67.3%) (ii) 1071/1620 (66.1%)
Baldi et al., 2020^6^	Lombardy, Italy	Registry-based study	Adult and pediatric cases regardless of etiology (EMS-treated NR; Received resuscitation NR)	(i) February 21–April 20, 2020 (ii) February 21–April 20, 2019	(i) 490 (ii) 321	(i) 77.0 (14.1) (ii) 77.3 (14.2)	(i) 321/490 (65.5%) (ii) 188/321 (58.6%)
Ball et al., 2020^20^	Victoria, Australia	Registry-based study	Adult cases regardless of etiology; EMS-treated and received resuscitation	(i) March 16–May 12, 2020 (ii) March 16–May 12, 2017–2019	(i) 380 (ii) 1218	(i) 67.7 (19.4) (ii) 65.7 (19.3)	(i) 250/380 (65.8%) (ii) 845/1218 (69.4%)
Cho et al., 2020^10^	Daegu, South Korea	Registry-based study	Adult cases of presumed medical etiology; EMS-treated and received resuscitation	(i) February 17–March 31, 2020 (ii) February 17–March 31, 2018	(i) 171 (ii) 158	(i) 72.0 (13.5) (ii) 72.8 (15.3)	(i) 108/171 (63.2%) (ii) 103/158 (65.2%)
Elmer et al., 2020^29^	Pennsylvania, USA	Registry-based study	Adult cases regardless of etiology; EMS-treated (Received resuscitation NR)	(i) March 1–May 25, 2020 (ii) January–February 2016–2020	(i) 683 (ii) 12,252	(i) 64.0 (19.0) (ii) 63.0 (19.0)	(i) 430/683 (63.0%) (ii) 7700/12,252 (62.8%)
Lai et al., 2020^7^	New York City, USA	Non-registry-based study	Adult cases regardless of etiology; EMS-treated and received resuscitation	(i) March 1–April 25, 2020 (ii) March 1–April 25, 2019	(i) 3989 (ii) 1336	(i) 72.0 (18.0) (ii) 68.0 (19.0)	(i) 2183/3989 (54.7%) (ii) 752/1336 (56.3%)
Marijon et al., 2020^8^	Paris, France	Registry-based study	Adult cases of non-traumatic etiology; EMS-treated (Received resuscitation NR)	(i) March 16–April 26, 2020 (ii) Weeks 12–17, 2012–2019	(i) 521 (ii) 3052	(i) 69.7 (17.0) (ii) 68.5 (18.0)	(i) 334/521 (64.1%) (ii) 1826/3052 (59.8%)
Ortiz et al., 2020^21^	Spain	Registry-based study	Adult and pediatric cases regardless of etiology; EMS-treated (Received resuscitation NR)	(i) February 1–April 30, 2020 (ii) April 1–30, 2017 and February 1–March 31, 2018	(i) 1446 (ii) 1723	(i) 64.4 (16.5) (ii) 65.6 (16.9)	(i) 1028/1446 (71.1%) (ii) 1210/1723 (70.2%)
Paoli et al., 2020^30^	Province of Padua, Italy	Non-registry-based study	Adult and pediatric cases regardless of etiology; EMS-treated (Received resuscitation NR)	(i) March 1–April 30, 2020 (ii) March 1–April 30, 2019	(i) 200 (ii) 206	(i) 79.0 (17.0) (ii) 77.0 (14.0)	(i) NR (ii) NR
Sayre et al., 2020^22^	Seattle and King County, USA	Registry-based study	Adult and pediatric cases regardless of etiology; EMS-treated (Received resuscitation NR)	(i) February 26–April 15, 2020 (ii) January 1–February 25, 2019	(i) 537 (ii) 530	(i) NR (ii) NR	(i) NR (ii) NR
Semeraro et al., 2020^31^	Bologna, Italy	Registry-based study	Adult cases regardless of etiology; EMS-treated and received resuscitation	(i) January 1–June 30, 2020 (ii) January 1–June 30, 2019	(i) 624 (ii) 563	(i) 82.7 (13.4) (ii) 82.7 (13.4)	(i) 318/624 (51.0%) (ii) 284/563 (50.4%)
Chan et al., 2021^23^	27 States and multiple Counties, USA	Registry-based study	Adult cases of non-traumatic etiology; EMS-treated (Received resuscitation NR)	(i) March 16–April 30, 2020 (ii) March 16–April 30, 2019	(i) 9863 (ii) 9440	(i) 62.6 (19.6) (ii) 62.2 (19.2)	(i) 6040/9863 (61.2%) (ii) 5922/9440 (62.7%)
de Koning et al., 2021^32^	Hollands-Midden, The Netherlands	Registry-based study	Adult cases regardless of etiology; EMS-treated (Received resuscitation NR)	(i) March 16–April 27, 2020 (ii) March 16–April 27, 2019	(i) 56 (ii) 45	(i) 70.0 (14.0) (ii) 70.0 (12.0)	(i) 32/56 (57.1%) (ii) 31/45 (68.9%)
Fothergill et al., 2021^11^	London, UK	Registry-based study	Adult and pediatric cases regardless of etiology; EMS-treated (Received resuscitation NR)	(i) March 1–April 30, 2020 (ii) March 1–April 30, 2019	(i) 3122 (ii) 1724	(i) 71.0 (19.0) (ii) 68.0 (20.0)	(i) 1839/3122 (58.9%) (ii) 1069/1724 (62.0%)
Glober et al., 2021^24^	Indiana (Marion County), USA	Registry-based study	Adult cases of non-traumatic etiology; EMS-treated (Received resuscitation NR)	(i) January 1–June 30, 2020 (ii) January 1–June 30, 2019	(i) 1034 (ii) 884	(i) 59.7 (18.5) (ii) 61.5 (18.1)	(i) 622/1034 (60.2%) (ii) 544/884 (61.5%)
Lim et al., 2021^13^	Singapore	Registry-based study	Adult cases regardless of etiology; EMS-treated (Received resuscitation NR)	(i) January 1–May 31, 2020 (ii) January 1–May 31, 2018–2019	(i) 1400 (ii) 1280	(i) 72.3 (17.8) (ii) 71.3 (17.1)	(i) 882/1400 (63.0%) (ii) 818/1280 (63.9%)
Mathew et al., 2021^25^	Detroit, USA	Registry-based study	Adult cases of non-traumatic etiology; EMS-treated and received resuscitation	(i) March 10–April 30, 2020 (ii) March 10–April 30, 2019	(i) 291 (ii) 180	(i) 64.5 (18.1) (ii) 58.5 (19.8)	(i) 165/291 (56.7%) (ii) 93/180 (51.7%)
Nickles et al., 2021^26^	Detroit (Macomb, Oakland, and Wayne Counties), USA	Registry-based study	Adult and pediatric cases of non-traumatic etiology; EMS-treated (Received resuscitation NR)	(i) January 1–May 31, 2020 (ii) January 1–May 31, 2019	(i) 1854 (ii) 1162	(i) NR (ii) NR	(i) 1083/1854 (58.4%) (ii) 662/1162 (57.0%)
Sultanian et al., 2021^27^	Sweden	Registry-based study	Adult and pediatric cases regardless of etiology; EMS-treated and received resuscitation	(i) March 16–July 20, 2020 (ii) January 1–March 16, 2020	(i) 1016 (ii) 930	(i) 69.6 (17.8) (ii) 70.8 (16.6)	(i) 697/1016 (68.6%) (ii) 604/930 (64.9%)
Uy-Evanado et al., 2021^28^	Oregon (Multnomah County) and California (Ventura County), USA	Registry-based study	Adult and pediatric cases regardless of etiology; EMS-treated and received resuscitation	(i) March 1–May 31, 2020 (ii) March 1–May 31, 2019	(i) 278 (ii) 231	(i) 64.9 (18.3) (ii) 69.1 (17.4)	(i) 174/278 (62.6%) (ii) 137/231 (59.3%)

*EMS* emergency medical services, *UK* United Kingdom, *USA* United States of America, *NR* not reported, *COVID-19* Coronavirus disease 2019, *SD* standard deviation, *N* number.

Study designs for all included studies were multicentered and retrospective in nature.

All studies achieved a score from seven to nine on the Newcastle–Ottawa Scale, signifying high quality and low risk of bias for selection (Supplementary Table 1).

#### Community processes

**Figure 2:**
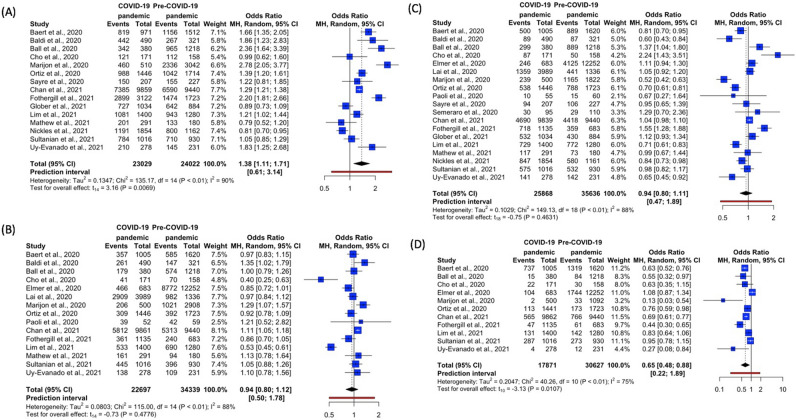
Forest plots for community processes—(**A**) OHCA at home (**B**) Unwitnessed OHCA (**C**) BCPR (**D**) AED Use. *AED *automated external defibrillator, *BCPR bystander* cardiopulmonary resuscitation, *OHCA* out-of-hospital cardiac arrest. R Core Team (2021). R: A language and environment for statistical computing. R Foundation for Statistical Computing, Vienna, Austria. URL https://www.R-project.org/.

Community processes-related outcomes analyzed in this study included OHCA at home, unwitnessed OHCA, BCPR, and AED use. A summary of community processes of care was shown in Table 2, while Fig. 2 depicted the forest plots of the various community processes.

**Table 2:** Summary of community processes of care.

Study	Time period	OHCA at home, N (%)	Unwitnessed OHCA, N (%)	BCPR, N (%)	AED use, N (%)
Baert et al., 2020^a^^19^	COVID-19 pandemic	819/971 (84.3%)	357/1005 (35.5%)	500/1005 (49.8%)	737/1005 (73.3%)
	Pre-COVID-19 pandemic	1156/1512 (76.5%)	585/1620 (36.1%)	889/1620 (54.9%)	1319/1620 (81.4%)
Baldi et al., 2020^6^	COVID-19 pandemic	442/490 (90.2%)	261/490 (53.3%)	89/490 (18.2%)	NR
	Pre-COVID-19 pandemic	267/321 (83.2%)	147/321 (45.8%)	87/321 (27.1%)	NR
Ball et al., 2020^20^	COVID-19 pandemic	342/380 (90.0%)	179/380 (47.1%)	299/380 (78.7%)	15/380 (3.9%)
	Pre-COVID-19 pandemic	965/1218 (79.2%)	574/1218 (47.1%)	889/1218 (73.0%)	84/1218 (6.9%)
Cho et al., 2020^10^	COVID-19 pandemic	121/171 (70.8%)	41/171 (24.0%)	87/171 (50.9%)	22/171 (12.9%)
	Pre-COVID-19 pandemic	112/158 (70.9%)	70/158 (44.3%)	50/158 (31.6%)	30/158 (19.0%)
Elmer et al., 2020^29^	COVID-19 pandemic	NR	466/683 (68.2%)	246/683 (36.0%)	104/683 (15.2%)
	Pre-COVID-19 pandemic	NR	8772/12,252 (71.6%)	4125/12,252 (33.7%)	1744/12,252 (14.2%)
Lai et al., 2020^7^	COVID-19 pandemic	NR	2909/3989 (72.9%)	1359/3989 (34.1%)	NR
	Pre-COVID-19 pandemic	NR	982/1336 (73.5%)	441/1336 (33.0%)	NR
Marijon et al., 2020^a^^8^	COVID-19 pandemic	460/510 (90.2%)	206/500 (41.2%)	239/500 (47.8%)	2/500 (0.4%)
	Pre-COVID-19 pandemic	2336/3042 (76.8%)	1021/2908 (35.1%)	1165/1822 (63.9%)	33/1092 (3.0%)
Ortiz et al., 2020^a^^21^	COVID-19 pandemic	988/1446 (68.3%)	309/1446 (21.4%)	538/1446 (37.2%)	113/1441 (7.8%)
	Pre-COVID-19 pandemic	1042/1714 (60.8%)	392/1723 (22.8%)	788/1723 (45.7%)	173/1723 (10.0%)
Paoli et al., 2020^a^^30^	COVID-19 pandemic	NR	39/52 (75.0%)	10/55 (18.2%)	NR
	Pre-COVID-19 pandemic	NR	42/59 (71.2%)	15/60 (25.0%)	NR
Sayre et al., 2020^22^	COVID-19 pandemic	150/207 (72.5%)	NR	94/207 (45.4%)	NR
	Pre-COVID-19 pandemic	155/227 (68.3%)	NR	106/227 (46.7%)	NR
Semeraro et al., 2020^31^	COVID-19 pandemic	NR	NR	30/95 (31.6%)	NR
	Pre-COVID-19 pandemic	NR	NR	29/110^b^ (26.4%)	NR
Chan et al., 2021^a^^23^	COVID-19 pandemic	7385/9859 (74.9%)	5812/9861 (58.9%)	4690/9839 (47.7%)	565/9862 (5.7%)
	Pre-COVID-19 pandemic	6590/9440 (69.8%)	5313/9440 (56.3%)	4418/9440 (46.8%)	766/9440 (8.1%)
de Koning et al., 2021^32^	COVID-19 pandemic	NR	NR	NR	NR
	Pre-COVID-19 pandemic	NR	NR	NR	NR
Fothergill et al., 2021^a^^11^	COVID-19 pandemic	2899/3122 (92.9%)	361/1135 (31.8%)	718/1135 (63.3%)	47/1135 (4.1%)
	Pre-COVID-19 pandemic	1474/1723 (85.5%)	240/683^b^ (35.1%)	359/683^b^ (52.6%)	61/683^b^ (8.9%)
Glober et al., 2021^24^	COVID-19 pandemic	727/1034 (70.3%)	NR	532/1034 (51.5%)	NR
	Pre-COVID-19 pandemic	642/884 (72.6%)	NR	430/884 (48.6%)	NR
Lim et al., 2021^13^	COVID-19 pandemic	1081/1400 (77.2%)	533/1400 (38.1%)	729/1400 (52.1%)	131/1400 (9.4%)
	Pre-COVID-19 pandemic	943/1280 (73.7%)	690/1280 (53.9%)	772/1280 (60.3%)	142/1280 (11.1%)
Mathew et al., 2021^25^	COVID-19 pandemic	201/291 (69.1%)	161/291 (55.3%)	117/291 (40.2%)	NR
	Pre-COVID-19 pandemic	133/180 (73.9%)	94/180 (52.2%)	73/180 (40.6%)	NR
Nickles et al., 2021^a^^26^	COVID-19 pandemic	1191/1854 (64.2%)	NR	847/1854 (45.7%)	NR
	Pre-COVID-19 pandemic	800/1162 (68.8%)	NR	580/1161 (50.0%)	NR
Sultanian et al., 2021^27^	COVID-19 pandemic	784/1016 (77.2%)	445/1016 (43.8%)	575/1016 (56.6%)	287/1016 (28.2%)
	Pre-COVID-19 pandemic	710/930 (76.3%)	396/930 (42.6%)	532/930 (57.2%)	273/930 (29.4%)
Uy-Evanado et al., 2021^28^	COVID-19 pandemic	210/278 (75.5%)	138/278 (49.6%)	141/278 (50.7%)	4/278 (1.4%)
	Pre-COVID-19 pandemic	145/231 (62.8%)	109/231 (47.2%)	142/231 (61.5%)	12/231 (5.2%)

*OHCA* out-of-hospital cardiac arrest, *BCPR* bystander cardiopulmonary resuscitation, *AED* automatic external defibrillator, *NR* not reported, *N* number. ^a^Difference in denominators is due to incomplete reporting of outcomes for certain patients. ^b^Among those in whom resuscitation was attempted by the Emergency Medical Services.

##### OHCA location at home

Fifteen studies accounted for the outcome of OHCA at home^6,8,10,11,13,19–28^. Amongst these, Nickles et al. reported the lowest percentage (64.2%) in the COVID-19 pandemic, while Ortiz et al. reported the lowest percentage (60.5%) of patients with OHCA at home prior to the COVID-19 pandemic^21,26^. In contrast, Fothergill et al. reported the highest percentage of patients with OHCA at home in both periods during and before the COVID-19 pandemic (92.9%, 85.5% respectively)^11^. With the exception of four studies^10,24–26^, which showed a lower percentage of patients with OHCA at home during as compared to before the pandemic, a trend was observed where the percentage of patients with OHCA at home was higher during the COVID-19 pandemic as compared to Pre-COVID-19 pandemic (Table 2).

Meta-analysis showed that the odds of patients undergoing OHCA at home was significantly higher during the pandemic as compared to before the pandemic (OR 1.38, 95% CI 1.11–1.71, p = 0.0069, *I*^2^ = 90%) (Fig. 2A).

##### Unwitnessed OHCA

Fifteen studies accounted for the number of unwitnessed OHCA events^6,8,10,13,19–21,23,25,27–30^. Ortiz et al. reported the lowest percentage of unwitnessed OHCA cases during (21.4%) and before the pandemic (22.8%)^21^. Meanwhile, Paoli et al. reported the highest percentage in the COVID-19 pandemic (75%) and Lai et al. reported the highest percentage for Pre-COVID-19 pandemic (73.5%) (Table 2)^7,30^. Meta-analysis showed that there was no difference in unwitnessed OHCA events during and before the pandemic (OR 0.94, 95% CI 0.80–1.12, p = 0.4776, *I*^2^ = 88%) (Fig. 2B).

##### BCPR

Nineteen studies accounted for BCPR rates^6–8,10,11,13,19–31^. In the COVID-19 pandemic, Paoli et al. and Baldi et al. both reported the lowest percentage for BCPR among patients (18.2%) while Ball et al. reported the highest percentage (78.7%) (Table 2)^6,20,30^. In Pre-COVID-19 pandemic, Paoli et al. reported the lowest percentage for BCPR among patients (25%) while Ball et al. reported the highest percentage (73%)^20,30^. Meta-analysis showed that there was no difference in BCPR during and before the pandemic (OR 0.94, 95% CI 0.80–1.11, p = 0.4631, *I*^2^ = 88%) (Fig. 2C).

##### AED use

Eleven studies accounted for AED use^8,10,11,13,19–21,23,27–29^. The percentage of the population with AED use ranged from 0.4 to 81.4% across intervals during and before the COVID-19 pandemic. Apart from Elmer et al.^29^, a trend of lower percentage of population with AED use was observed during the COVID-19 pandemic as compared to prior to the pandemic. Across intervals during and before the COVID-19 pandemic, almost all studies reported a percentage AED use of 29.4% or less. Only Baert et al. reported relatively higher percentages during the COVID-19 pandemic and prior to the pandemic (73.3% and 81.4% respectively) (Table 2)^19^. Meta-analysis showed that the odds of OHCA patients using AED was significantly lower during the pandemic as compared to prior to the pandemic (OR 0.65, 95% CI 0.48–0.88, p = 0.0107, *I*^2^ = 75%) (Fig. 2D).

#### EMS processes

**Figure 3:**
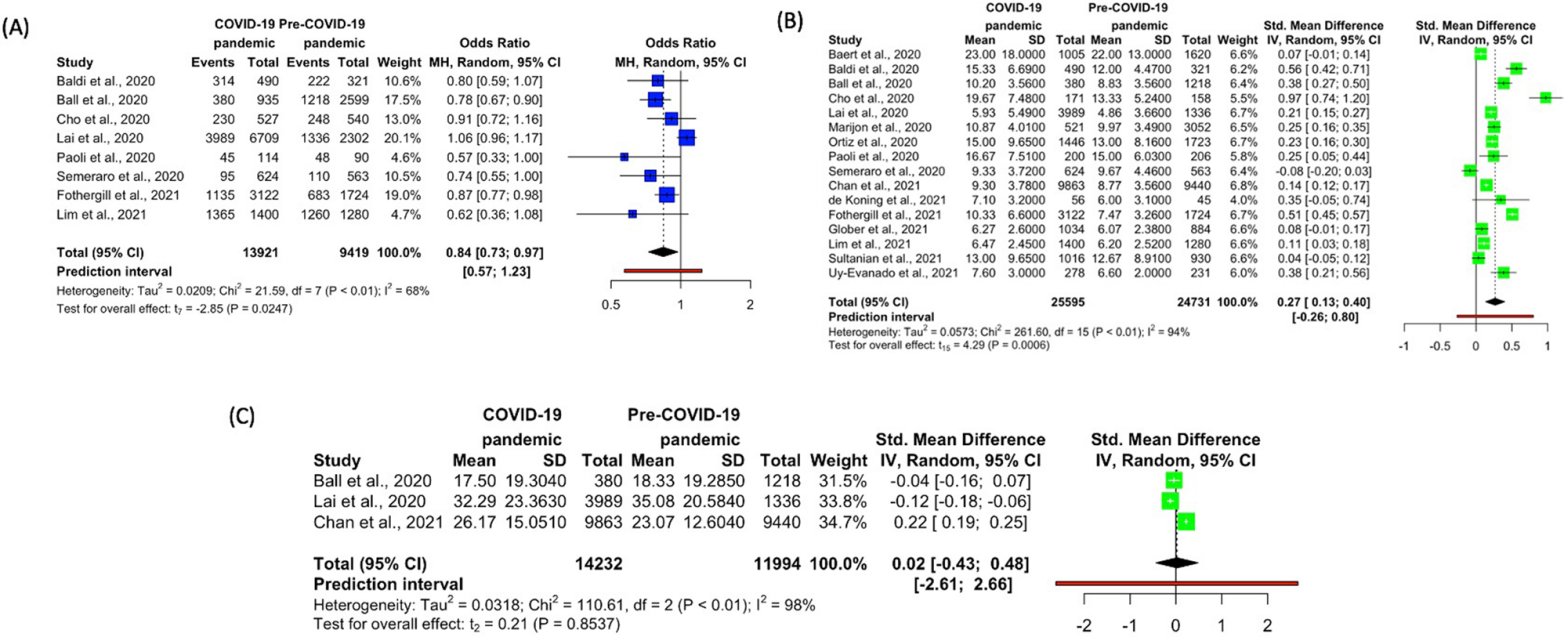
Forest plots for EMS processes—(**A**) EMS resuscitation attempted (**B**) EMS call to arrival time (**C**) Resuscitation duration. *EMS* emergency medical services. R Core Team (2021). R: A language and environment for statistical computing. R Foundation for Statistical Computing, Vienna, Austria. URL https://www.R-project.org/.

**Figure 4:**
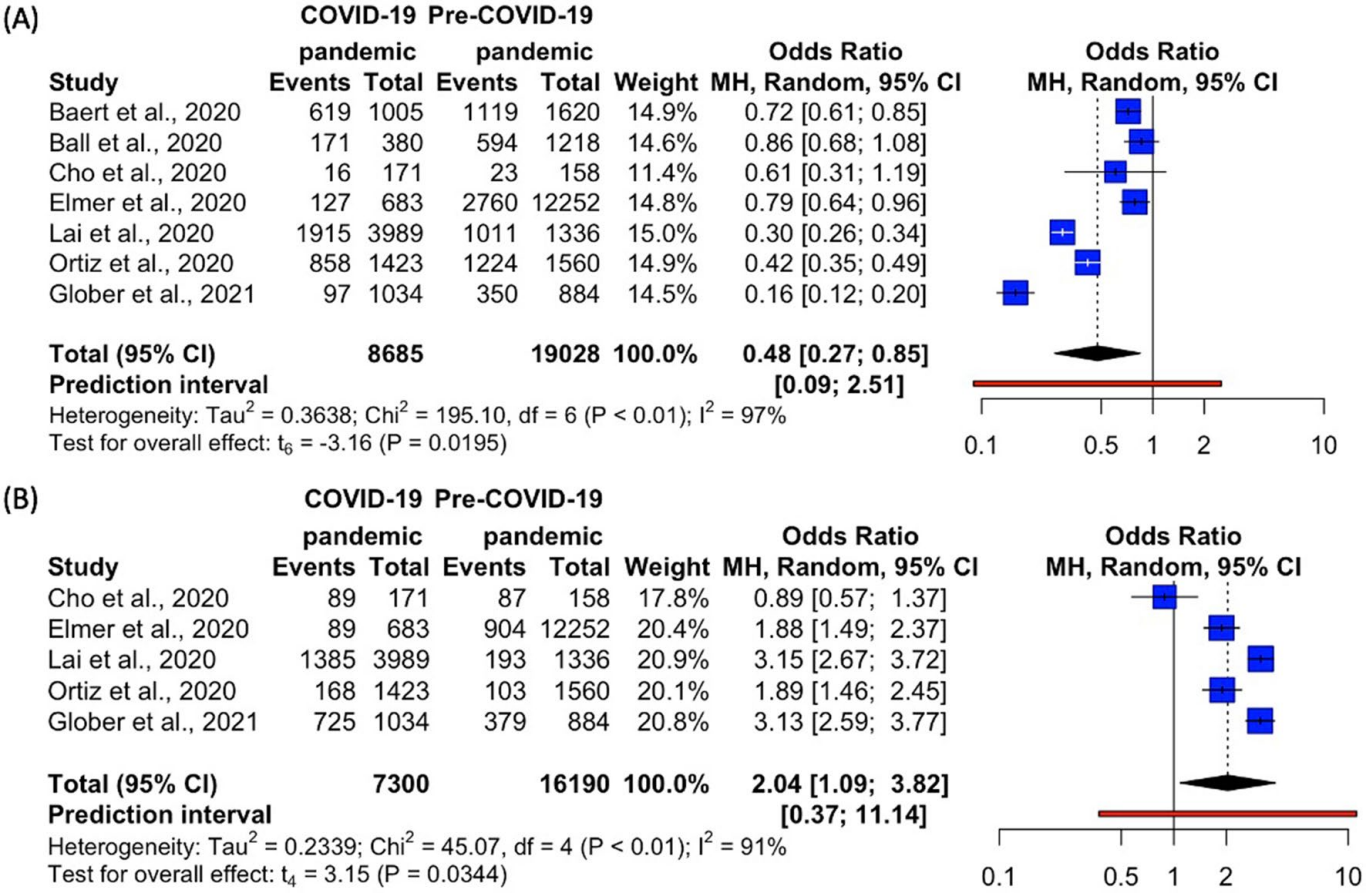
Forest plots for EMS processes—(**A**) Endotracheal Intubation (**B**) Supraglottic Airway. *EMS*, emergency medical services. R Core Team (2021). R: A language and environment for statistical computing. R Foundation for Statistical Computing, Vienna, Austria. URL https://www.R-project.org/.

**Figure 5:**
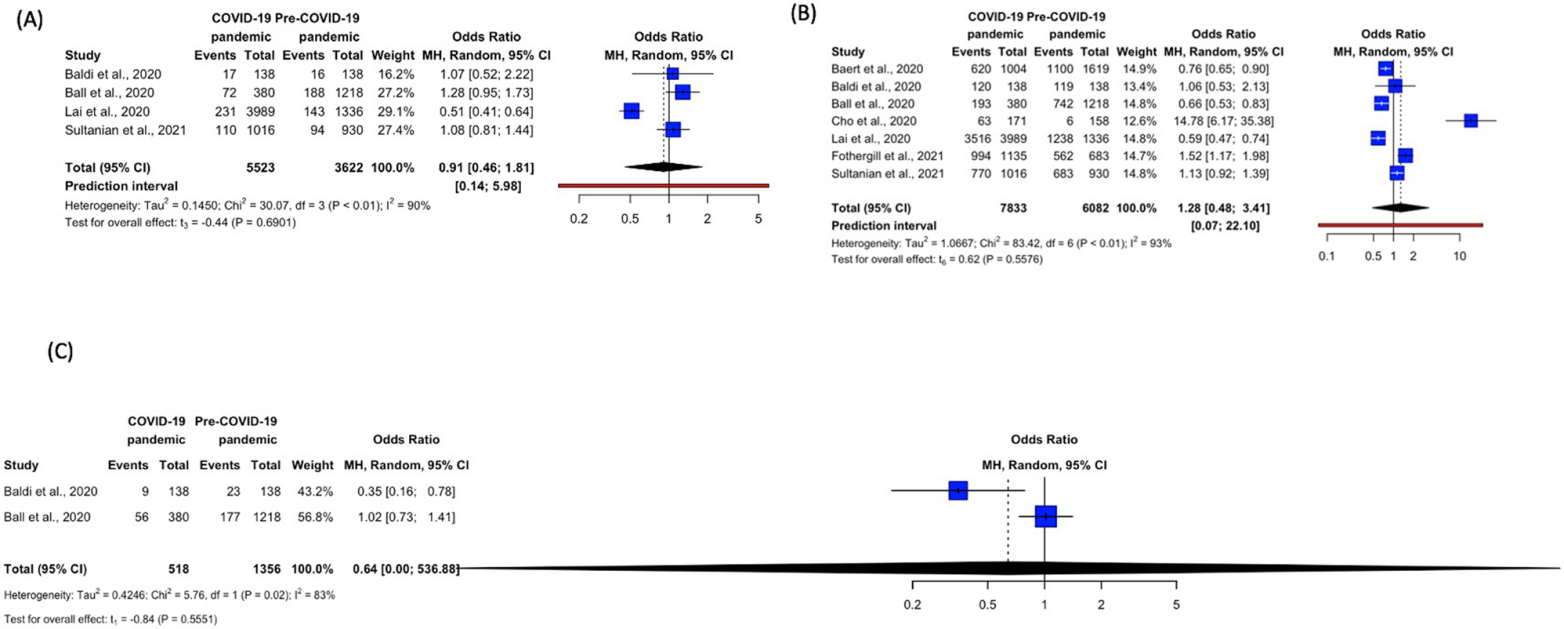
Forest plots for EMS processes—(**A**) Amiodarone (**B**) Epinephrine (**C**) Mechanical CPR. *CPR *cardiopulmonary resuscitation, *EMS* emergency medical services. R Core Team (2021). R: A language and environment for statistical computing. R Foundation for Statistical Computing, Vienna, Austria. URL https://www.R-project.org/.

EMS processes-related outcomes analyzed in this study included EMS resuscitation attempted, resuscitation duration, EMS call to arrival time, endotracheal intubation and supraglottic airway, amiodarone and epinephrine, and use of mechanical CPR. A summary of EMS processes of care was shown in Table 3, while Figs. 3, 4 and 5 depicted the forest plots of the various EMS processes.

**Table 3:** Summary of emergency medical services processes of care.

Study	Time period	EMS resuscitation attempted, N (%)	Resuscitation duration (min), Mean (SD)	EMS call to arrival time (min), mean (SD)	Supraglottic airway, N (%)	Endotracheal intubation, N (%)	Mechanical CPR, N (%)	Amiodarone, N (%)	Epinephrine, N (%)
Baert et al., 2020^a^^19^	COVID-19 pandemic	NR	NR	23.0 (18.0)	NR	619/1005 (61.6%)	NR	NR	620/1004 (61.8%)
	Pre-COVID-19 pandemic	NR	NR	22.0 (13.0)	NR	1119/1620 (69.1%)	NR	NR	1100/1619 (67.9%)
Baldi et al., 2020^a^^6^	COVID-19 pandemic	314/490 (64.1%)	NR	15.3 (6.7)	NR	NR	9/138 (6.5%)	17/138 (12.3%)	120/138 (87.0%)
	Pre-COVID-19 pandemic	222/321 (69.2%)	NR	12.0 (4.5)	NR	NR	23/138 (16.7%)	16/138 (11.6%)	119/138 (86.2%)
Ball et al., 2020^a^^20^	COVID-19 pandemic	380/935 (40.6%)	17.5 (19.3)	10.2 (3.6)	NR	171/380 (45.0%)	56/380 (14.7%)	72/380 (18.9%)	193/380 (50.8%)
	Pre-COVID-19 pandemic	1218/2599 (46.9%)	18.3 (19.3)	8.8 (3.6)	NR	594/1218 (48.8%)	177/1218 (14.5%)	188/1218 (15.4%)	742/1218 (60.9%)
Cho et al., 2020^a^^10^	COVID-19 pandemic	230/527 (43.6%)	NR	19.7 (7.5)	89/171 (52.0%)	16/171 (9.4%)	NR	NR	63/171 (36.8%)
	Pre-COVID-19 pandemic	248/540 (45.9%)	NR	13.3 (5.2)	87/158 (55.1%)	23/158 (14.6%)	NR	NR	6/158 (3.8%)
Elmer et al., 2020^29^	COVID-19 pandemic	NR	NR	NR	89/683 (13.0%)	127/683 (18.6%)	NR	NR	NR
	Pre-COVID-19 pandemic	NR	NR	NR	904/12,252 (7.4%)	2760/12,252 (22.5%)	NR	NR	NR
Lai et al., 2020^a^^7^	COVID-19 pandemic	3989/6709 (59.5%)	32.3 (23.4)	5.9 (5.5)	1385/3989 (34.7%)	1915/3989 (48.0%)	NR	231/3989 (5.8%)	3516/3989 (88.1%)
	Pre-COVID-19 pandemic	1336/2302 (58.0%)	35.1 (20.6)	4.9 (3.7)	193/1336 (14.4%)	1011/1336 (75.7%)	NR	143/1336 (10.7%)	1238/1336 (92.7%)
Marijon et al., 2020^8^	COVID-19 pandemic	NR	NR	10.9 (4.0)	NR	NR	NR	NR	NR
	Pre-COVID-19 pandemic	NR	NR	10.0 (3.5)	NR	NR	NR	NR	NR
Ortiz et al., 2020^21^	COVID-19 pandemic	NR	NR	15.0 (9.7)	168/1423 (11.8%)	858/1423 (60.3%)	NR	NR	NR
	Pre-COVID-19 pandemic	NR	NR	13.0 (8.2)	103/1560 (6.6%)	1224/1560 (78.5%)	NR	NR	NR
Paoli et al., 2020^30^	COVID-19 pandemic	45/114 (39.5%)	NR	16.7 (7.5)	NR	NR	NR	NR	NR
	Pre-COVID-19 pandemic	48/90 (53.3%)	NR	15.0 (6.0)	NR	NR	NR	NR	NR
Sayre et al., 2020^22^	COVID-19 pandemic	NR	NR	NR	NR	NR	NR	NR	NR
	Pre-COVID-19 pandemic	NR	NR	NR	NR	NR	NR	NR	NR
Semeraro et al., 2020^31^	COVID-19 pandemic	95/624 (15.2%)	NR	9.3 (3.7)	NR	NR	NR	NR	NR
	Pre-COVID-19 pandemic	110/563 (19.5%)	NR	9.7 (4.5)	NR	NR	NR	NR	NR
Chan et al., 2021^23^	COVID-19 pandemic	NR	26.2 (15.1)	9.3 (3.8)	NR	NR	NR	NR	NR
	Pre-COVID-19 pandemic	NR	23.1 (12.6)	8.8 (3.6)	NR	NR	NR	NR	NR
de Koning et al., 2021^32^	COVID-19 pandemic	NR	NR	7.1 (3.2)	NR	NR	NR	NR	NR
	Pre-COVID-19 pandemic	NR	NR	6.0 (3.1)	NR	NR	NR	NR	NR
Fothergill et al., 2021^a^^11^	COVID-19 pandemic	1135/3122 (36.4%)	NR	10.3 (6.6)	NR	NR	NR	NR	994/1135 (87.6%)
	Pre-COVID-19 pandemic	683/1724 (39.6%)	NR	7.5 (3.3)	NR	NR	NR	NR	562/683^b^ (82.3%)
Glober et al., 2021^24^	COVID-19 pandemic	NR	NR	6.3 (2.6)	725/1034 (70.1%)	97/1034 (9.4%)	NR	NR	NR
	Pre-COVID-19 pandemic	NR	NR	6.1 (2.4)	379/884 (42.9%)	350/884 (39.6%)	NR	NR	NR
Lim et al., 2021^13^	COVID-19 pandemic	1365/1400 (97.5%)	NR	6.5 (2.5)	NR	NR	NR	NR	NR
	Pre-COVID-19 pandemic	1260/1280 (98.4%)	NR	6.2 (2.5)	NR	NR	NR	NR	NR
Mathew et al., 2021^25^	COVID-19 pandemic	NR	NR	NR	NR	NR	NR	NR	NR
	Pre-COVID-19 pandemic	NR	NR	NR	NR	NR	NR	NR	NR
Nickles et al., 2021^26^	COVID-19 pandemic	NR	NR	NR	NR	NR	NR	NR	NR
	Pre-COVID-19 pandemic	NR	NR	NR	NR	NR	NR	NR	NR
Sultanian et al., 2021^27^	COVID-19 pandemic	NR	NR	13.0 (9.7)	NR	NR	NR	110/1016 (10.8%)	770/1016 (75.8%)
	Pre-COVID-19 pandemic	NR	NR	12.7 (8.9)	NR	NR	NR	94/930 (10.1%)	683/930 (73.4%)
Uy-Evanado et al., 2021^28^	COVID-19 pandemic	NR	NR	7.6 (3.0)	NR	NR	NR	NR	NR
	Pre-COVID-19 pandemic	NR	NR	6.6 (2.0)	NR	NR	NR	NR	NR

*EMS* emergency medical services, *OHCA* out-of-hospital cardiac arrest, *CPR* cardiopulmonary resuscitation, *ALS* advanced life support, *NR* not reported, *N* number, *SD* standard deviation. ^a^Difference in denominators is due to incomplete reporting of outcomes for certain patients. ^b^Among those in whom resuscitation was attempted by the Emergency Medical Services.

##### EMS resuscitation attempted

Eight studies accounted for the outcome of EMS resuscitation attempted^6,7,10,11,13,20,30,31^. Apart from Lai et al.^7^, all other studies reported a lower percentage of population with EMS resuscitation attempted in the COVID-19 pandemic as compared to Pre-COVID-19 pandemic (Table 3).

Meta-analysis showed that the odds of EMS resuscitation attempted on OHCA patients was significantly lower during the pandemic as compared to before the pandemic (OR 0.84, 95% CI 0.73–0.97, p = 0.0247, *I*^2^ = 68%) (Fig. 3A).

##### EMS call to arrival time

Sixteen studies accounted for the outcome of EMS call to arrival time^6–8,10,11,13,19–21,23,24,27,28,30–32^. Mean duration of EMS call to arrival time ranged from 4.9 to 23 min across time periods during the COVID-19 pandemic and before the pandemic. Apart from Semeraro et al.^31^, a trend of longer mean duration of EMS call to arrival time was observed during the pandemic as compared to prior to the pandemic (Table 3). Meta-analysis showed that there was a significant difference in EMS call to arrival time between time periods in the COVID-19 pandemic and Pre-COVID-19 pandemic (SMD 0.27, 95% CI 0.13–0.40, p = 0.0006, *I*^2^ = 94%) (Fig. 3B).

##### Resuscitation duration

Only three studies accounted for resuscitation duration^7,20,23^. Apart from Chan et al.^23^, all other studies reported a shorter mean duration of resuscitation in the COVID-19 pandemic as compared to Pre-COVID-19 pandemic (Table 3). Meta-analysis showed that there was no difference in resuscitation duration between time periods in the COVID-19 pandemic and Pre-COVID-19 pandemic (SMD 0.02, 95% CI − 0.43–0.48, p = 0.8537, *I*^2^ = 98%) (Fig. 3C).

##### Endotracheal intubation and supraglottic airway

Seven studies accounted for the use of endotracheal intubation^7,10,19–21,24,29^, while five studies accounted for the use of supraglottic airway^7,10,21,24,29^. All relevant studies reported a lower percentage of endotracheal intubation use in the COVID-19 pandemic as compared to Pre-COVID-19 pandemic. In contrast, almost all studies (except Cho et al.^10^) reported a higher percentage of supraglottic airway use in the COVID-19 pandemic as compared to Pre-COVID-19 pandemic (Table 3). Meta-analysis showed that the odds of using endotracheal intubation on OHCA patients was significantly lower in the COVID-19 pandemic as compared to Pre-COVID-19 pandemic (OR 0.48, 95% CI 0.27–0.85, p = 0.0195, *I*^2^ = 97%) (Fig. 4A). Meanwhile, the odds of using supraglottic airways on OHCA patients was significantly higher in the COVID-19 pandemic as compared to Pre-COVID-19 pandemic (OR 2.04, 95% CI 1.09–3.82, p = 0.0344, *I*^2^ = 91%) (Fig. 4B).

##### Amiodarone and epinephrine

Four studies accounted for the use of amiodarone^6,7,20,27^, while seven studies accounted for the use of epinephrine^6,7,10,11,19,20,27^. Apart from Lai et al.^7^, all other studies reported a higher percentage of amiodarone use in the COVID-19 pandemic relative to Pre-COVID-19 pandemic. The trend was not as obvious in epinephrine usage; four studies^6,10,11,27^ reported a higher percentage of epinephrine use during the pandemic as compared to before the pandemic, while three studies reported an inverse occurrence (Table 3). Meta-analysis showed that there was no difference in OHCA patients receiving amiodarone in the COVID-19 pandemic and Pre-COVID-19 pandemic (OR 0.91, 95% CI 0.46–1.81, p = 0.6901, *I*^2^ = 90%) (Fig. 5A). Similarly, there was no difference in OHCA patients receiving epinephrine in the COVID-19 pandemic and Pre-COVID-19 pandemic (OR 1.28, 95% CI 0.48–3.41, p = 0.5576, *I*^2^ = 93%) (Fig. 5B).

##### Mechanical CPR

Only two studies accounted for the use of mechanical CPR^6,20^. Baldi et al. reported a lower percentage of mechanical CPR use during the COVID-19 pandemic as compared to before the pandemic while Ball et al. reported the converse (Table 3)^6,20^. Meta-analysis showed that there was no difference in the use of mechanical CPR for OHCA patients in the COVID-19 pandemic and Pre-COVID-19 pandemic (OR 0.64, 95% CI 0.0008–536.8771, p = 0.5551, *I*^2^ = 83%) (Fig. 5C).

### Sensitivity analyses

#### Leave-one-out analyses

After visual inspection of forest plots for potential, sensitivity analyses were performed using influential diagnostic plots and Baujat plots. Applying this approach on each outcome of community and EMS processes, none of the estimates were substantially changed in direction or statistical significance. However, the magnitude of effect was increased in six outcomes: 18.5% for EMS call to arrival time, 600% for resuscitation duration, 18.75% for endotracheal intubation, 20.6% for supraglottic airway, 27.5% for amiodarone and 30.5% for epinephrine. The revised estimates on sensitivity analyses were: OHCA at home (OR 1.44 [95% CI 1.16–1.79, p = 0.0031, I^2^ = 87%] after excluding Nickles et al.), unwitnessed OHCA (OR 0.99 [95% CI 0.86–1.15, p = 0.9214, I^2^ = 70%] after excluding Lim et al.), BCPR (OR 0.98 [95% CI 0.83–1.14, p = 0.7582, I^2^ = 85%] after excluding Marijon et al.), AED use (OR 0.61 [95% CI 0.45–0.84, p = 0.0064, I^2^ = 66%] after excluding Elmer et al.), EMS resuscitation attempted (OR 0.80 [95% CI 0.71–0.90, p = 0.0034, I^2^ = 0%] after excluding Lai et al.), EMS call to arrival time (SMD 0.22 [95% CI 0.12–0.32, p = 0.0003, I^2^ = 94%] after excluding Cho et al.), resuscitation duration (SMD − 0.10 [95% CI − 0.57–0.38, p = 0.2355, I^2^ = 30%] after excluding Chan et al.), endotracheal intubation (OR 0.57 [95% CI 0.36–0.89, p = 0.0236, I^2^ = 96%] after excluding Glober et al.), supraglottic airway (OR 2.46 [95% CI 1.54–3.92, p = 0.0087, I^2^ = 86%] after excluding Cho et al.), amiodarone (OR 1.16 [95% CI 0.90–1.51, p = 0.1278, I^2^ = 0%] after excluding Lai et al.), and epinephrine (OR 0.89 [95% CI 0.60–1.31, p = 0.4643, I^2^ = 88%] after excluding Cho et al.) (Supplementary Figs. 2–34). Ultimately, the final outcomes presented in this study were not based on adjustment with sensitivity analyses as none of the estimates were substantially changed in direction or statistical significance, showing that the sensitivity analyses did not greatly influence the findings of our review and thus, no articles were determined to be excluded.

#### Subgroup analyses: BCPR

In order to account for possible moderators that might contribute to statistical heterogeneity, subgroup analyses were conducted for the outcome of BCPR which had the highest number of studies. The subgroup analyses were based on categorical variables, namely, publication year and study location. None of the permutations yielded a statistically significant difference. Statistical heterogeneity remained high in all subgroup analyses. We did not conduct subgroup analyses for all other outcomes due to the inadequate data (Supplementary Table 3).

#### Meta-regression: BCPR

Meta-regression with a mixed-effects model was performed for the outcome of BCPR to examine if the observed heterogeneity could be contributed by possible moderators such as sample size, mean age, proportion of males, proportion of patients with OHCA at residential location, GDP per country, GDP per state and population density of study region (km^2^). Univariate meta-regression did not reveal any statistically significant moderators. Meta-regression analyses were only conducted for the outcome of BCPR as it had the highest number of studies. We did not conduct meta-regression for other outcomes due to the inadequate data (Supplementary Table 4).

### Publication bias

A funnel plot was generated based on the outcome BCPR which had the highest number of studies, revealing no asymmetry, hence suggesting the absence of publication bias (Supplementary Fig. 1). This was congruent with a non-significant Egger’s regression test (p = 0.62).

## Discussion

In this study, we elucidated several salient findings. First, there was a significant increase in OHCA occurring at home and a significant decrease in bystander AED use during the COVID-19 pandemic. Second, there was no difference in BCPR rates during the pandemic as compared to before. Third, the pandemic was associated with changes to EMS processes including a significant decrease in attempted EMS resuscitation, a significant increase in EMS call to arrival times, a significant decrease in endotracheal intubation, and a significant increase in supraglottic airway use.

The increase in OHCA at home is consistent with stay-at-home and social distancing measures worldwide during the COVID-19 pandemic^33^. As more people are enforced to stay or work from home^34^, OHCA at home rates have consequently increased. Importantly, this finding also highlights that witness rates for OHCA incidence are similar during the pandemic as compared to before. This is necessary to consider for future prehospital interventions as subsequent measures should be targeted at the accessibility of services and devices and less at the detection of OHCA in the chain of survival^35^. This finding is supported by a statistically significant decrease in bystander AED use during the COVID-19 pandemic, which could be attributed to an increase of OHCA occurring at home^36,37^. The lack of AED availability in homes may principally explain this observation, along with inaccessibility of AEDs installed in public buildings due to the sudden closure of non-essential businesses and services by government policies^36^. Accordingly, new perspectives for current AED guidelines may be warranted as OHCA at home rates have increased during the pandemic, highlighting the shortcomings of current AED policies. More strategic placements of AEDs should be carried out to maximize access from individual homes, with particular emphasis on rural areas where AED accessibility is relatively more limited^36,38^. Given the potentially long-lasting lifestyle changes from the COVID-19 pandemic, the rising rates of OHCA at home should be urgently addressed.

Interestingly, we found no statistical difference in BCPR during the pandemic as compared to before. This is in contrast to previous literature, which suggested that observed decreases in BCPR resulted from fear of COVID-19 disease transmission from patient to bystanders or hesitancy among family members towards performing CPR for OHCA at home due to psychological and emotional reasons^8,13,39^. This result could be explained by an attitude-behavior gap whereby family members at home perform CPR despite such hesitancy^40^. Moreover, it could also be attributed to certain recommendations that CPR is not an aerosol-generating procedure^41–43^, thus, decreasing the likelihood for COVID-19 to be transmitted. A recent study with swine models validates such findings, where the authors found that chest compressions alone did not cause significant aerosol generation in the swine model^44^. However, our findings should be interpreted with caution as an analysis on BCPR rates for home versus non-home arrests could not be performed due to paucity of data. Furthermore, this result should be interpreted in light of significant statistical heterogeneity unexplained by the sociodemographic, economic status, and geographical location of the sample population in each study. Although no differences were found in BCPR before and after the COVID-19 pandemic, this does not mean that campaigns or interventions encouraging CPR during the pandemic should cease, which should instead be carefully tailored to each country’s unique response to the pandemic at the societal and individual levels^45,46^.

The decrease in EMS resuscitation attempts was significant, contrary to previous studies^22,47^. The COVID-19 pandemic directly led to a severe strain on ambulance resources, changes in EMS workflows, and sicker OHCA patients, possibly leading to fewer patients qualifying for EMS resuscitation in an attempt to redirect scarce resources and maximize lives saved when crisis standards of care are enforced^20,48^. Additionally, protocol changes could have exacerbated the decrease in EMS resuscitation attempts during the pandemic. For example, in Detroit, EMS protocols were amended to include the termination of resuscitation in suspected COVID-19 cases after ten minutes of resuscitation without return of spontaneous circulation^25^. The scarcity of resources is also consistent with the increase in EMS call to arrival times, which could be attributed to PPE requirements during the pandemic and increased ambulance travel distance for OHCA patients at home^10^. Cho et al. reported an emphasis on high-level PPE, consistent with an exaggerated increase in EMS call to arrival time and did not modify the significance of the effect size when excluded during leave-one-out analysis^10^. Although our study found a relatively smaller magnitude of SMD estimate for EMS call to arrival times, there remained a statistically significant increase in EMS call to arrival times during the pandemic. This has added importance in a time-critical medical emergency such as OHCA, where every second counts. Interestingly, the increase in EMS call to arrival times occurred despite reported road traffic reduction during the pandemic^3^. This suggests that while the lighter road traffic may partially improve EMS call to arrival times during the pandemic, it did not completely offset the delay from PPE donning and COVID-19 related strain on ambulance resources. More studies are required to report the time between EMS departure and arrival time in order to arrive at more definitive conclusions. Finally, a decrease in endotracheal intubation was accompanied by an increase in supraglottic airway use, likely reflecting the perceived risks of COVID-19 transmission in endotracheal intubation^49^. Certain protocol revisions could have also contributed to the increase in intubation use, as these guidelines recommended the use of supraglottic airways over endotracheal intubation^24^. While the overload of healthcare systems is to be expected during a pandemic, this should not come at the cost of worsening outcomes for non-COVID illnesses including OHCA. More needs to be done to find a balance in resource allocation when saving lives affected by COVID-19 or other non-COVID life-threatening diseases. Better preparation and predefined protocols are needed for emergency care systems to operate under resource-scarce crisis situations.

These findings hold implications for future pre-hospital interventions. A growing body of evidence demonstrates significant changes to OHCA characteristics during the COVID-19 pandemic which impact public health and urgently need to be addressed^13,19^. Efforts to manage the effects of the pandemic, which may be the chief priority for public health institutions, should not come at the cost of worsening outcomes for non-COVID illnesses including OHCA. Future pre-hospital OHCA measures should also be targeted at the accessibility of services and devices and less at the detection of OHCA in the chain of survival. Systematic placement of AEDs should be carried out to maximize access from individual homes, with particular emphasis on rural areas where AED accessibility is relatively more limited^36,50^. Although no differences were found in BCPR before and after the COVID-19 pandemic, educational campaigns or interventions encouraging CPR during the pandemic should continue to be championed for and carefully tailored to each country. This would improve maintenance of personal safety on an individual level and increase empowerment of rescuers on a societal level^38^. The negative changes to EMS processes associated with the pandemic are worrying and suggest that better preparation and predefined protocols are needed for emergency care systems to operate under resource-scarce crisis situations, including the stockpiling and effective use of PPE^51^. A clear transition from non-crisis to crisis resource allocation coupled with clear public health messaging will likely be beneficial. Additionally, a centralized public EMS system may improve coordination between different stakeholders during the COVID-19 pandemic and reduce OHCA mortality rates^52,53^. However, more investigation is needed in this field. Further research is also needed to investigate measures for minimizing COVID-19 transmission during resuscitation, such as supraglottic airway use and mechanical CPR, as well as the barriers to implementing them during the pandemic.

The results of this study were robust to sensitivity analyses and incorporated data from several large OHCA registries from various countries. No publication bias was detected on visual inspection and statistical analysis. However, the findings of this study should be interpreted in the context of known limitations. All included studies were observational cross-sectional studies comparing the COVID-19 period to a historical control. Hence, results were vulnerable to confounding and should be interpreted carefully. Moderate to high statistical heterogeneity was encountered during analyses. Heterogeneity in the definition of the COVID-19 pandemic and Pre-COVID-19 pandemic may have led to varying estimates of effect size, depending on local epidemiology. Moreover, the included studies had varying time periods for the COVID-19 pandemic. Each study was unable to reflect an equal severity of the pandemic, which may have contributed to the heterogeneity encountered. Differences in study characteristics such as demographics, surveillance, and data collection processes also likely further accounted for observed heterogeneity. All included studies originated from first-world countries that comprised populations with higher income and higher socio-economic status. Hence, study results may be limited in terms of generalizability despite providing key insights into OHCA during the COVID-19 pandemic.

However, this study is, to our knowledge, one of the first few systematic reviews and meta-analyses to examine the impact of the COVID-19 pandemic on community and EMS care processes. It represents a global body of literature that may inform future prehospital interventions and guide the interpretation of changes in OHCA characteristics during the COVID-19 pandemic.

## Conclusion

BCPR rates remained unchanged before and during the COVID-19 pandemic, while outcomes of OHCA in the home and bystander AED increased. Ambulance processes remained largely unchanged, although EMS resuscitation attempts decreased and call to arrival times increased slightly. These findings may inform future interventions, particularly to consider interventions to increase BCPR and improve the pre-hospital chain of survival for future implementation.

## Supplementary Information

Supplementary Information.

## Data Availability

The data presented in this study are available in Supplementary Materials—Appendix I, Supplemental Figures, Supplemental Tables and Supplemental Data.
