# Supplementary material for: Variation in community and ambulance care processes for out-of-hospital cardiac arrest during the COVID-19 pandemic: a systematic review and meta-analysis
Source: Sci Rep. 2022 Jan 17;12:800. doi: 10.1038/s41598-021-04749-9 (PMC8764072; doi:10.1038/s41598-021-04749-9)
Supplement: Supplementary file 1 — Supplementary Information. [file 41598_2021_4749_MOESM1_ESM.docx]

**SUPPLEMENTAL MATERIAL**

**VARIATION IN COMMUNITY AND AMBULANCE CARE PROCESSES FOR OUT-OF-HOSPITAL CARDIAC ARREST DURING THE COVID-19 PANDEMIC: A SYSTEMATIC REVIEW AND META-ANALYSIS**

**ss**

1. **Appendix I – Expanded Methods**
2. **Supplemental Tables**
3. **Supplemental Figures**
4. **Supplemental Data**
5. **Appendix I – Expanded Methods**

**Appendix I. Full Search Phrases Used for the Five Respective Databases.**

| **PubMed** | | | | **165 articles** |
| --- | --- | --- | --- | --- |
| ((sudden cardiac arrest[Title/Abstract]) OR (sudden cardiac death[Title/Abstract]) OR (out of hospital cardiac arrest[Title/Abstract]) OR (out-of-hospital cardiac arrest[Title/Abstract]) OR (cardiac arrest[Title/Abstract]) OR (OHCA[Title/Abstract]) OR (OOHCA[Title/Abstract]) OR (“heart arrest”[Mesh])) AND ((covid-19[Title/Abstract]) OR (coronavirus[Title/Abstract]) OR (SARS-CoV-2[Title/Abstract])) | | | | |
|  | | | | |
| **EMBASE** | | | | **345 articles** |
| Heart arrest concept | | | | |
| 1 | ‘cardiac arrest’:ti,ab,kw | | | |
| 2 | ‘sudden cardiac arrest’:ti,ab,kw | | | |
| 3 | ‘sudden cardiac death’:ti,ab,kw | | | |
| 4 | ‘out of hospital cardiac arrest’:ti,ab,kw | | | |
| 5 | ‘out-of-hospital cardiac arrest’:ti,ab,kw | | | |
| 6 | OOHCA:ti,ab,kw | | | |
| 7 | OHCA:ti,ab,kw | | | |
| 8 | ‘heart arrest’/exp | | | |
| 9 | #1 OR #2 OR #3 OR #4 OR #5 OR #6 OR #7 OR #8 | | | |
| COVID-19 concept | | | | |
| 10 | covid-19:ti,ab,kw | | | |
| 11 | coronavirus:ti,ab,kw | | | |
| 12 | SARS-CoV-2:ti,ab,kw | | | |
| 13 | #10 OR #11 OR #12 | | | |
| Combined | | | | |
| 14 | #9 AND #13 | | | |
| **Web of Science** |  | | **43 articles** | |
| Heart arrest concept | | | | |
| 1 | TI=(sudden cardiac death OR sudden cardiac arrest OR OOHCA OR OHCA OR out of hospital cardiac arrest OR out-of-hospital cardiac arrest OR cardiac arrest) | | | |
| 2 | AB=(sudden cardiac death OR sudden cardiac arrest OR OOHCA OR OHCA OR out of hospital cardiac arrest OR out-of-hospital cardiac arrest OR cardiac arrest) | | | |
| 3 | #1 AND #2 | | | |
| COVID-19 concept | | | | |
| 4 | TI=(coronavirus OR covid-19 OR sars-cov-2) | | | |
| 5 | AB=(coronavirus OR covid-19 OR sars-cov-2) | | | |
| 6 | #4 AND #5 | | | |
| Combined | | | | |
| 7 | #3 AND #6 | | | |
| **SCOPUS** | | **408 articles** | | |
| Heart arrest concept | | | | |
| 1 | TITLE-ABS-KEY ( ( "OOHCA" OR "OHCA" OR "out of hospital cardiac arrest" OR "out-of-hospital cardiac arrest" OR "sudden cardiac arrest" OR "sudden cardiac death" OR "cardiac arrest" ) ) | | | |
| COVID-19 concept | | | | |
| 2 | TITLE-ABS-KEY ( ( "covid-19"  OR  "coronavirus"  OR  "SARS-CoV-2" ) ) | | | |
| Combined | | | | |
| 3 | #1 AND #2 | | | |
| **Cochrane Controlled Register of Trials (CENTRAL)** | | | **5 articles** | |
| Heart arrest concept | | | | |
| 1 | (cardiac arrest):ti,ab,kw | | | |
| 2 | (sudden cardiac arrest):ti,ab,kw | | | |
| 3 | (sudden cardiac death):ti,ab,kw | | | |
| 4 | (out of hospital cardiac arrest):ti,ab,kw | | | |
| 5 | (out-of-hospital cardiac arrest):ti,ab,kw | | | |
| 6 | (OOHCA):ti,ab,kw | | | |
| 7 | (OHCA):ti,ab,kw | | | |
| 8 | MeSH descriptor: [Heart arrest] explode all trees | | | |
| 9 | #1 OR #2 OR #3 OR #4 OR #5 OR #6 OR #7 OR #8 | | | |
| COVID-19 concept | | | | |
| 10 | (covid-19):ti,ab,kw | | | |
| 11 | (coronavirus):ti,ab,kw | | | |
| 12 | (SARS-CoV-2):ti,ab,kw | | | |
| 13 | #10 OR #11 OR #12 | | | |
| Combined | | | | |
| 14 | #9 AND #13 | | | |

Date searched: May 3, 2021

Total articles: 966

After endnote deduplication: 546

1. **Supplemental Tables**

**Supplemental Table 1. The Newcastle-Ottawa Scale (NOS) for risk of bias assessment of studies included in the meta-analysis**

| **Study** | **Selection** | | | | | | | | **Comparability** | | **Outcomes** | | | | | | **Total** | |
| --- | --- | --- | --- | --- | --- | --- | --- | --- | --- | --- | --- | --- | --- | --- | --- | --- | --- | --- |
|  | **Representativeness of exposed cohort** | | **Selection of nonexposed cohort** | | **Ascertainment of exposure** | | **Outcome not present at the start of the study** | | **Comparability on the basis of the design or analysis** | | **Assessment of outcomes** | | **Length of follow-up** | | **Adequacy of follow-up** | |  | |
|  | **YM** | **SET** | **YM** | **SET** | **YM** | **SET** | **YM** | **SET** | **YM** | **SET** | **YM** | **SET** | **YM** | **SET** | **YM** | **SET** | **YM** | **SET** |
| Baert et al., 2020 [19] | * | * | * | * | * | * | * | * | ** | ** | * | * | * | * |  |  | ******** | ******** |
| Baldi et al., 2020 [6] | * | * | * | * | * | * | * | * | ** | ** | * | * | * | * | * | * | ********* | ********* |
| Ball et al., 2020 [20] | * | * | * | * | * | * | * | * | * | * | * |  | * | * | * | * | ******** | ******** |
| Cho et al., 2020 [10] | * | * | * | * | * | * | * |  | ** | * | * | * |  | * |  | * | ******* | ******** |
| Elmer et al., 2020 [29] | * | * | * | * | * | * | * | * | ** | ** | * | * | * |  | * | * | ********* | ******** |
| Lai et al., 2020 [7] | * | * | * | * | * | * | * | * | * | ** | * | * | * | * | * | * | ******** | ********* |
| Marijon et al., 2020 [8] | * | * | * | * | * | * | * | * | ** | ** | * | * | * | * | * | * | ********* | ********* |
| Ortiz et al., 2020 [21] | * | * | * | * | * | * | * |  | ** | * | * | * | * | * | * | * | ********* | ******* |
| Paoli et al., 2020 [30] | * | * | * | * | * | * | * | * | ** | ** | * | * | * | * | * |  | ********* | ******** |
| Sayre et al., 2020 [22] | * |  | * | * | * | * | * | * | * | ** | * | * | * | * | * | * | ******** | ******** |
| Semeraro et al., 2020 [31] | * | * | * | * | * | * | * | * | ** | ** | * | * |  | * |  |  | ******* | ******** |
| Chan et al., 2021 [23] | * | * | * | * | * | * | * | * | * | * | * | * | * |  | * | * | ******** | ******* |
| de Koning et al., 2021 [32] | * | * | * | * | * | * | * |  | ** | ** | * | * | * | * |  |  | ******** | ******* |
| Fothergill et al., 2021 [11] | * | * | * | * | * | * | * | * | ** | ** | * | * | * | * | * | * | ********* | ********* |
| Glober et al., 2021 [24] | * |  | * | * | * |  | * | * | ** | ** | * | * | * | * | * | * | ********* | ******* |
| Lim et al., 2021 [13] | * | * | * | * | * | * | * | * | * | ** | * | * | * | * | * | * | ******** | ********* |
| Mathew et al., 2021 [25] | * | * | * | * | * | * | * | * | ** | * | * | * | * | * | * | * | ********* | ******** |
| Nickles et al., 2021 [26] | * | * | * | * | * | * | * | * | ** | ** | * | * |  | * |  |  | ******* | ******** |
| Sultanian et al., 2021 [27] | * | * | * | * | * | * | * | * | * | ** | * | * | * | * | * | * | ******** | ********* |
| Uy-Evanado et al., 2021 [28] | * | * | * | * | * | * | * | * | ** | * | * |  | * | * | * | * | ********* | ******* |

**Supplemental Table 2. Summary of Overall Findings**

| **OHCA Processes of Care** | **Parameters** | **Number of Studies** | **Pooled OR / SMD (95% CI)** | **P value** | **I^2^ Statistic** |
| --- | --- | --- | --- | --- | --- |
| Community Processes | OHCA at Home | 15 | 1.38 (1.11, 1.71) | 0.0069 | 90% |
|  | Unwitnessed OHCA | 15 | 0.94 (0.80, 1.12) | 0.4776 | 88% |
|  | BCPR | 19 | 0.94 (0.80, 1.11) | 0.4631 | 88% |
|  | AED Use | 11 | 0.65 (0.48, 0.88) | 0.0107 | 75% |
| EMS Processes | EMS Resuscitation Attempted | 8 | 0.84 (0.73, 0.97) | 0.0247 | 68% |
|  | EMS Call to Arrival Time | 16 | 0.27 (0.13, 0.40) | 0.0006 | 94% |
|  | Resuscitation Duration | 3 | 0.02 (-0.43, 0.48) | 0.8537 | 98% |
|  | Endotracheal Intubation | 7 | 0.48 (0.27, 0.85) | 0.0195 | 97% |
|  | Supraglottic Airway | 5 | 2.04 (1.09, 3.82) | 0.0344 | 91% |
|  | Amiodarone | 4 | 0.91 (0.46, 1.81) | 0.6901 | 90% |
|  | Epinephrine | 7 | 1.28 (0.48, 3.41) | 0.5576 | 93% |
|  | Mechanical CPR | 2 | 0.64 (0.0008, 536.8771) | 0.5551 | 83% |

*EMS*, emergency medical services; *OHCA*, out-of-hospital cardiac arrest; *BCPR*, bystander cardiopulmonary resuscitation; *AED*, automatic external defibrillator; *OR*, odds ratio; *CI*, confidence interval; *SMD*, standardized mean difference

**Supplemental Table 3. Subgroup Analysis**

| **Variables** | **OR** | **95% CI** | **I*^2^*** | ***P subgroup*** |
| --- | --- | --- | --- | --- |
| **Publication Year** |  | | | 0.8510 |
| 2020 | 0.9332 | 0.7052 – 1.2350 | 0.884 |  |
| 2021 | 0.9613 | 0.7676 – 1.2038 | 0.867 |  |
| **Location** |  | | | 0.2485 |
| France | 0.6513 | 0.0361 – 11.7360 | 0.919 |  |
| Italy | 0.7783 | 0.2718 – 2.2287 | 0.580 |  |
| Others | 1.1013 | 0.6918 – 1.7530 | 0.934 |  |
| USA | 0.9639 | 0.8319 – 1.1169 | 0.551 |  |

*OR*, odds ratio; *CI*, confidence interval; *USA*, United States of America

**Supplemental Table 4. Meta-Regression Analysis**

|  | **Univariate Analysis** | | | |
| --- | --- | --- | --- | --- |
| **Variables** | **Coeff** | **SE** | **95% CI** | **P value** |
| Sample Size | 0.0000 | 0.0000 | -0.0000 – 0.0000 | 0.6258 |
| Mean Age (Years) | -0.0041 | 0.0172 | -0.0409 – 0.0326 | 0.8129 |
| Male Gender (%) | -0.7562 | 1.8797 | -4.7626 – 3.2503 | 0.6932 |
| OHCA at Home | 0.5664 | 1.3780 | -2.4105 – 3.5433 | 0.6877 |
| Country Gross Domestic Product (per trillion, USD) | 0.0019 | 0.0084 | -0.0157 – 0.0196 | 0.8184 |
| State Gross Domestic Product (per billion, USD) | 0.0001 | 0.0001 | -0.0002 – 0.0004 | 0.4543 |
| Population Density (per km^2^) | -0.0000 | 0.0000 | -0.0000 – 0.0000 | 0.7034 |

*Coeff*, coefficient; *SE*, standard error; *CI*, confidence interval; *USD*, United States dollar

1. **Supplemental Figures**

**Supplemental Figure 1. Funnel Plot for Publication Bias based on Bystander Cardiopulmonary Resuscitation**

**
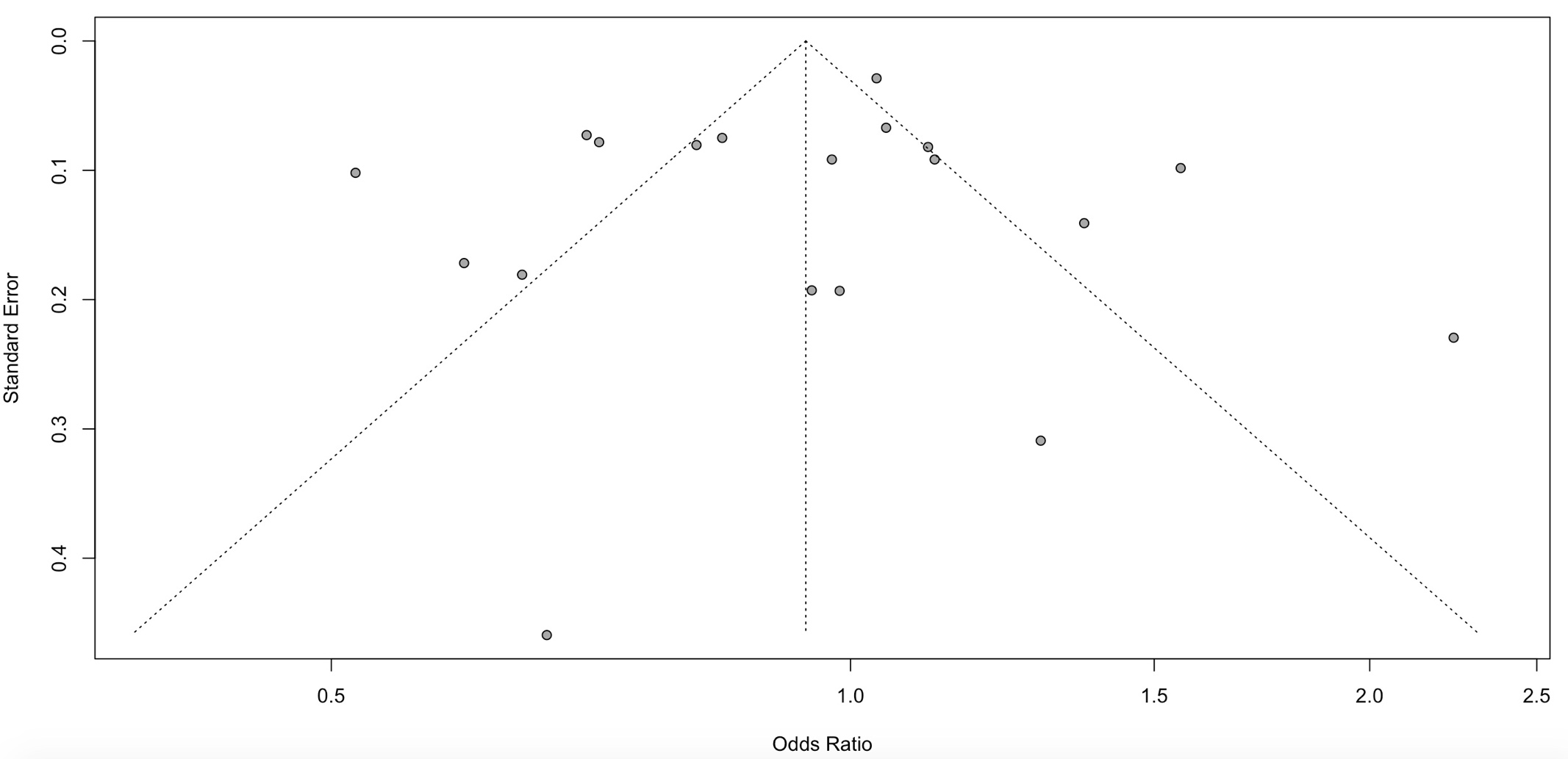
**

R Core Team (2021). R: A language and environment for statistical computing. R Foundation for Statistical Computing, Vienna, Austria. URL https://www.R-project.org/.

**Supplemental Figure 2. Influential Diagnostic Plot for Out-of-Hospital Cardiac Arrest At Home**


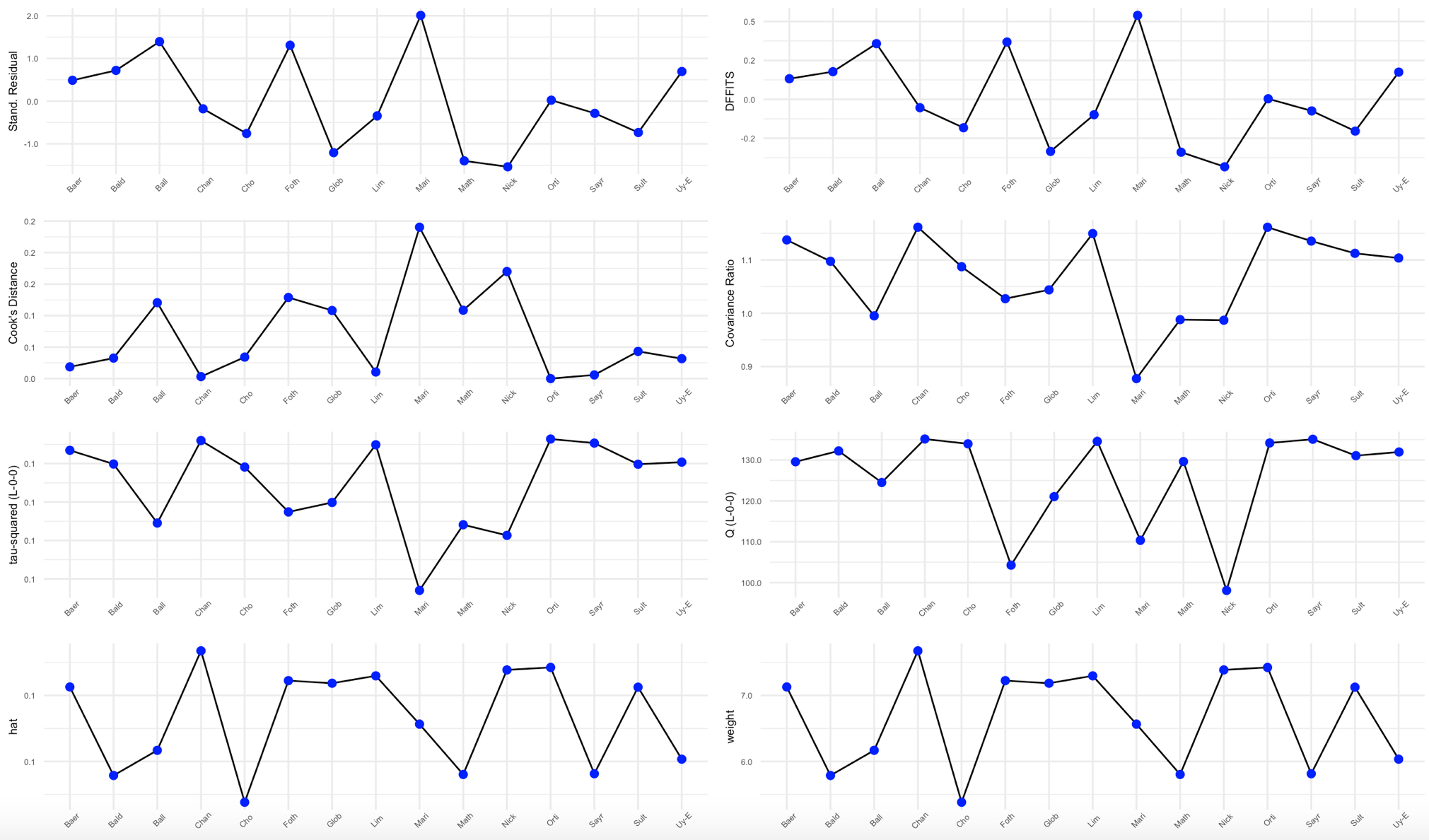


R Core Team (2021). R: A language and environment for statistical computing. R Foundation for Statistical Computing, Vienna, Austria. URL https://www.R-project.org/.

**Supplemental Figure 3. Baujat Plot for Out-of-Hospital Cardiac Arrest At Home**


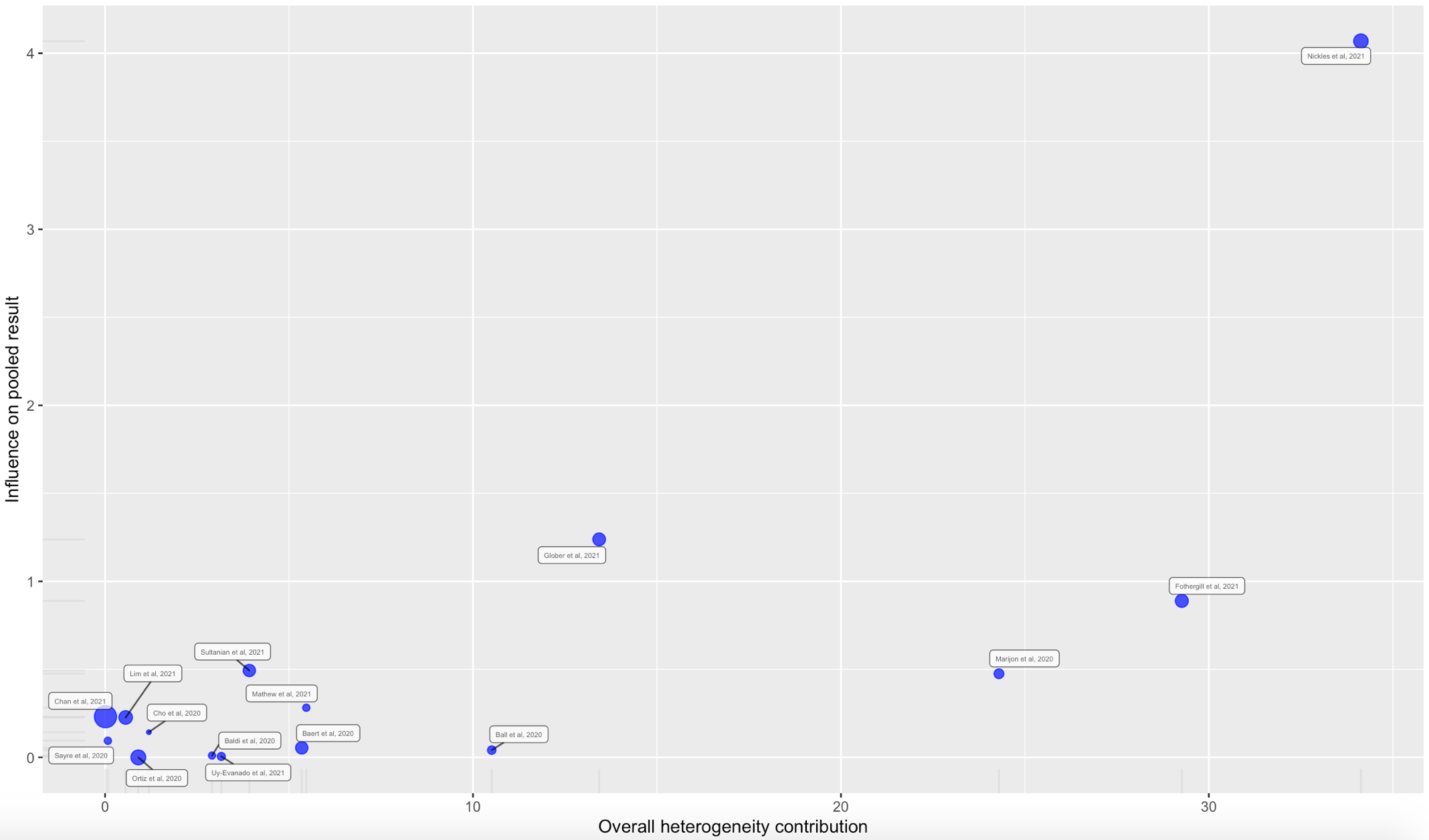


R Core Team (2021). R: A language and environment for statistical computing. R Foundation for Statistical Computing, Vienna, Austria. URL https://www.R-project.org/.

**Supplemental Figure 4. Leave-One-Out Analysis for Out-of-Hospital Cardiac Arrest At Home**


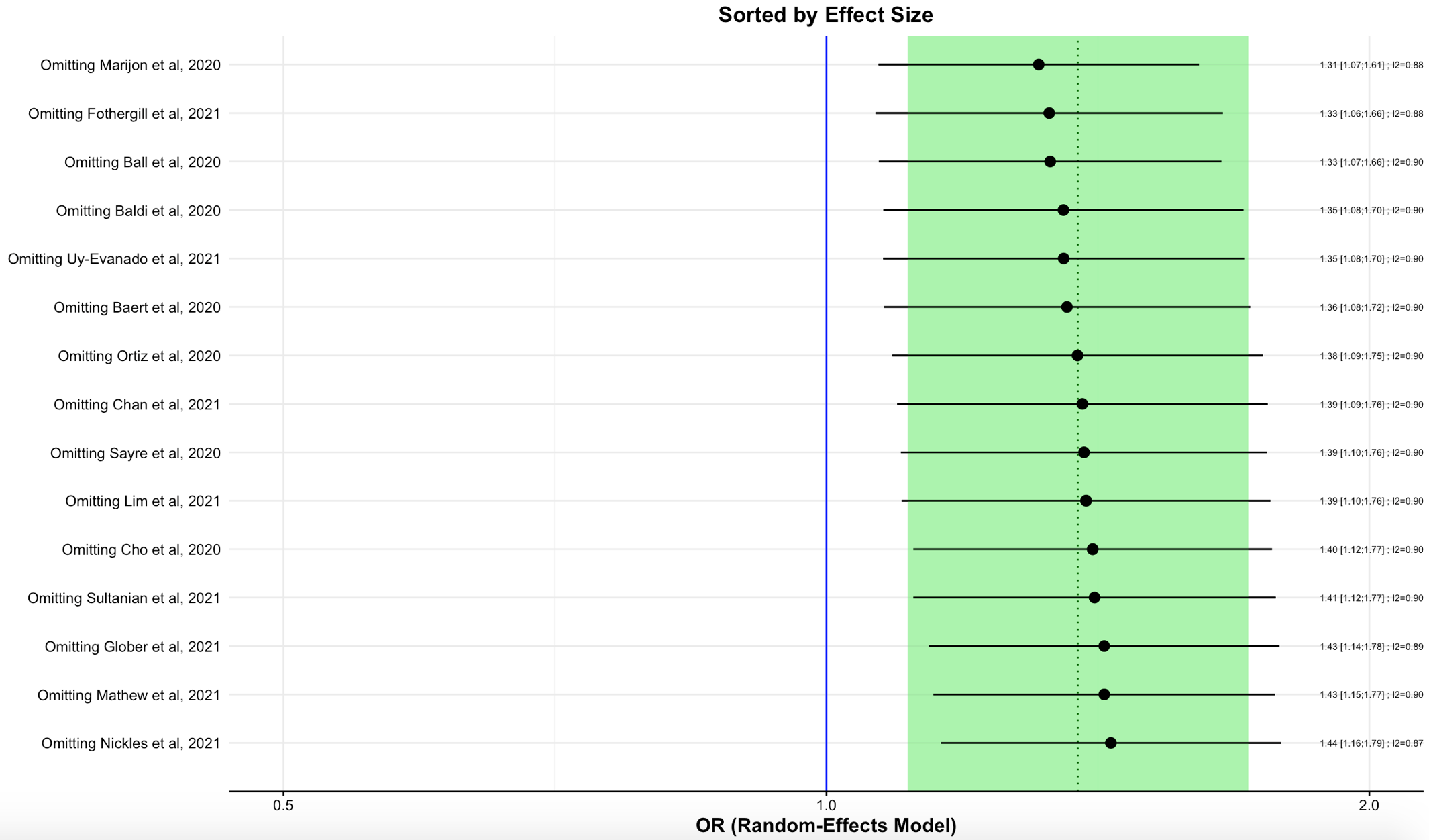


R Core Team (2021). R: A language and environment for statistical computing. R Foundation for Statistical Computing, Vienna, Austria. URL https://www.R-project.org/.

**Supplemental Figure 5. Influential Diagnostic Plot for Unwitnessed Out-of-Hospital Cardiac Arrest**


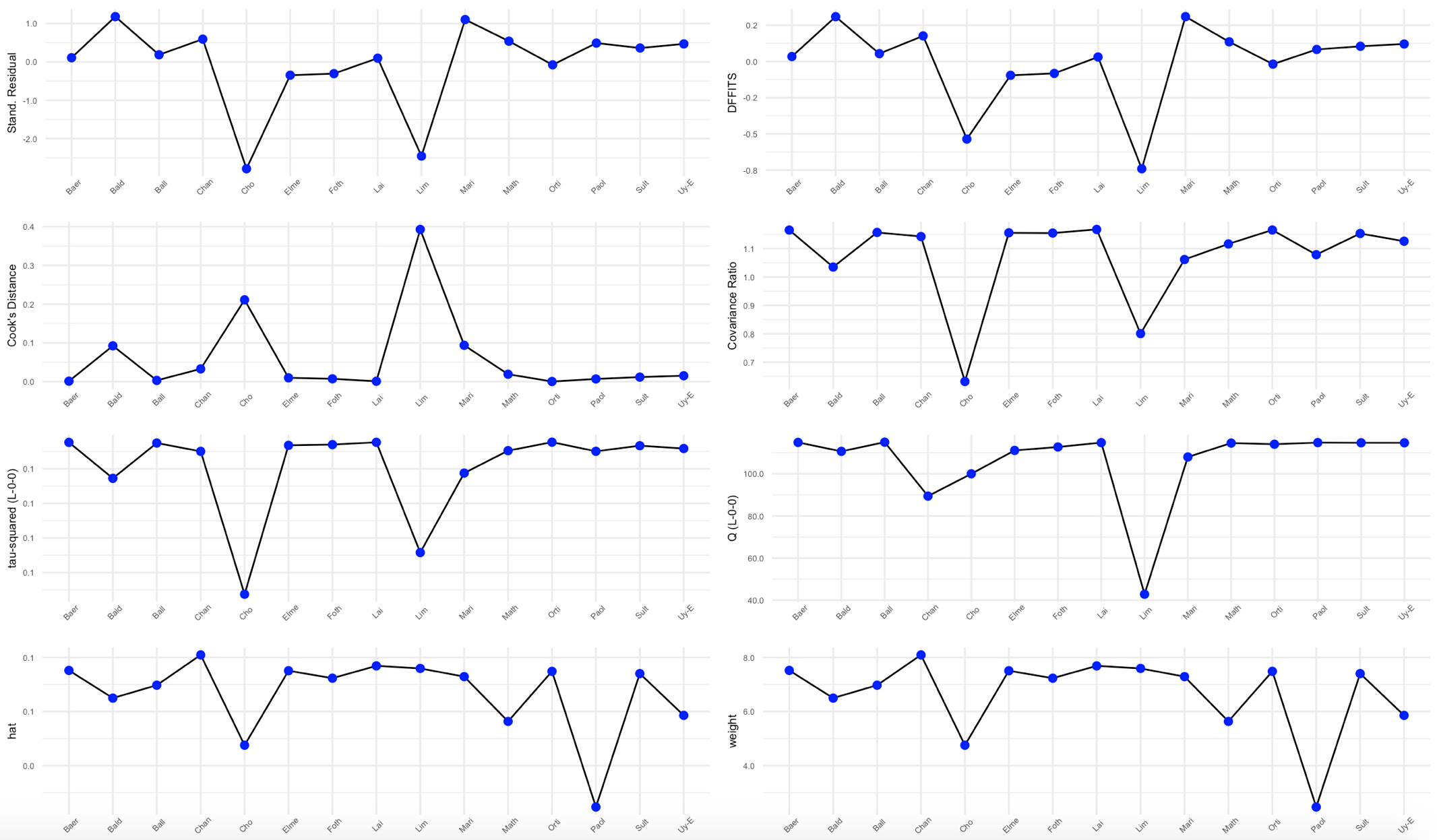


R Core Team (2021). R: A language and environment for statistical computing. R Foundation for Statistical Computing, Vienna, Austria. URL https://www.R-project.org/.

**Supplemental Figure 6. Baujat Plot for Unwitnessed Out-of-Hospital Cardiac Arrest**


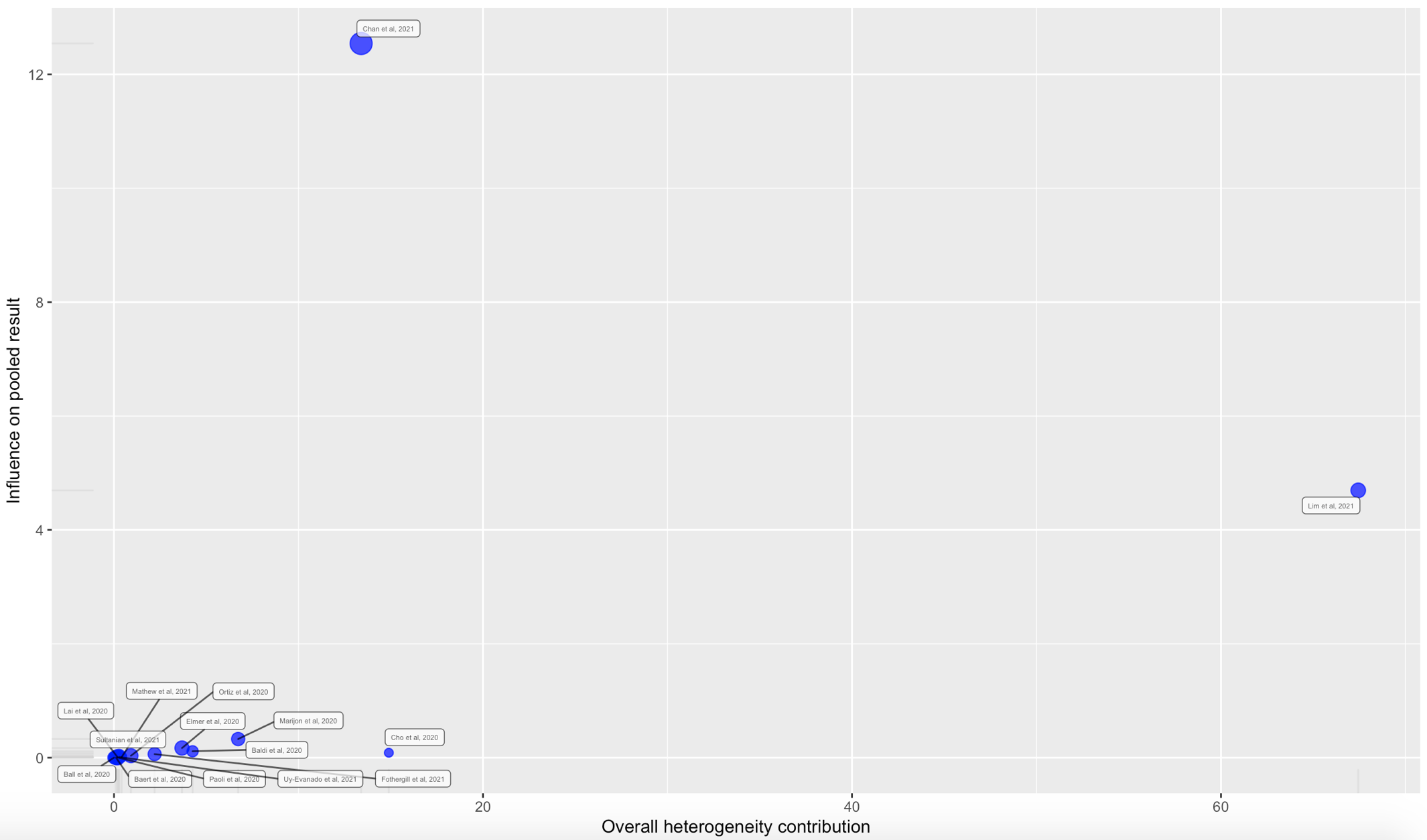


R Core Team (2021). R: A language and environment for statistical computing. R Foundation for Statistical Computing, Vienna, Austria. URL https://www.R-project.org/.

**Supplemental Figure 7. Leave-One-Out Analysis for Unwitnessed Out-of-Hospital Cardiac Arrest**


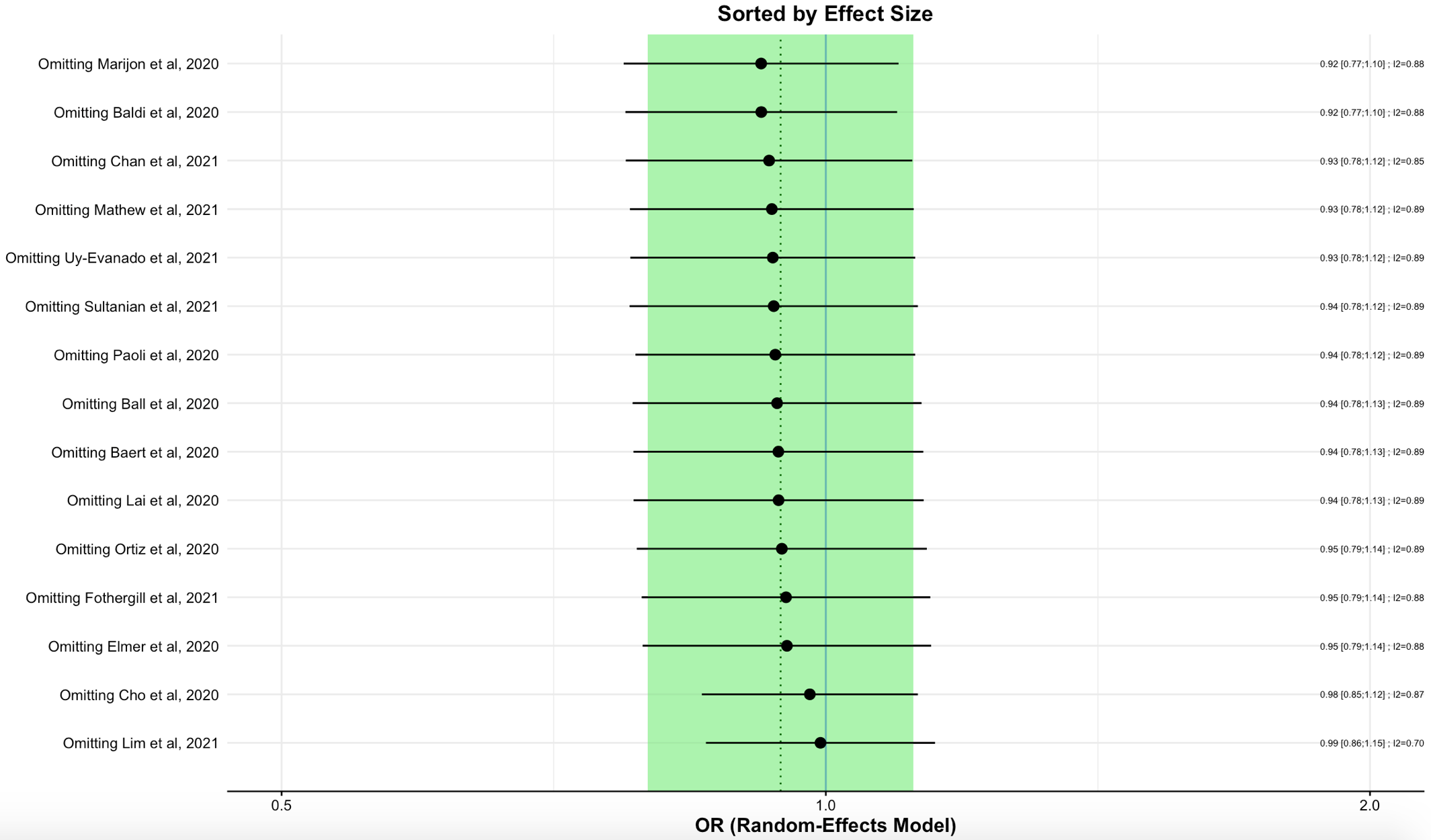


R Core Team (2021). R: A language and environment for statistical computing. R Foundation for Statistical Computing, Vienna, Austria. URL https://www.R-project.org/.

**Supplemental Figure 8. Influential Diagnostic Plot for Bystander Cardiopulmonary Resuscitation**


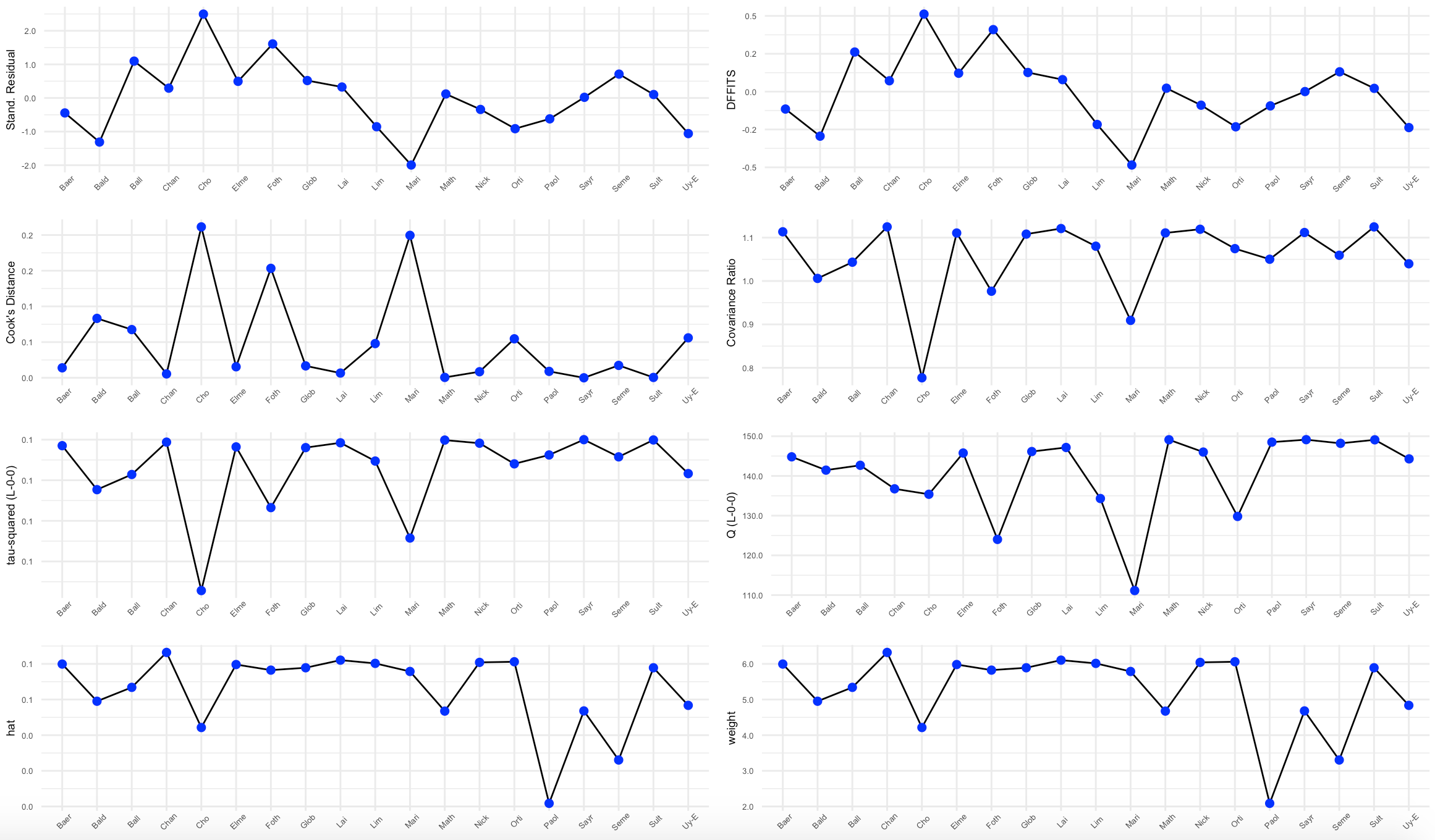


R Core Team (2021). R: A language and environment for statistical computing. R Foundation for Statistical Computing, Vienna, Austria. URL https://www.R-project.org/.

**Supplemental Figure 9. Baujat Plot for Bystander Cardiopulmonary Resuscitation**


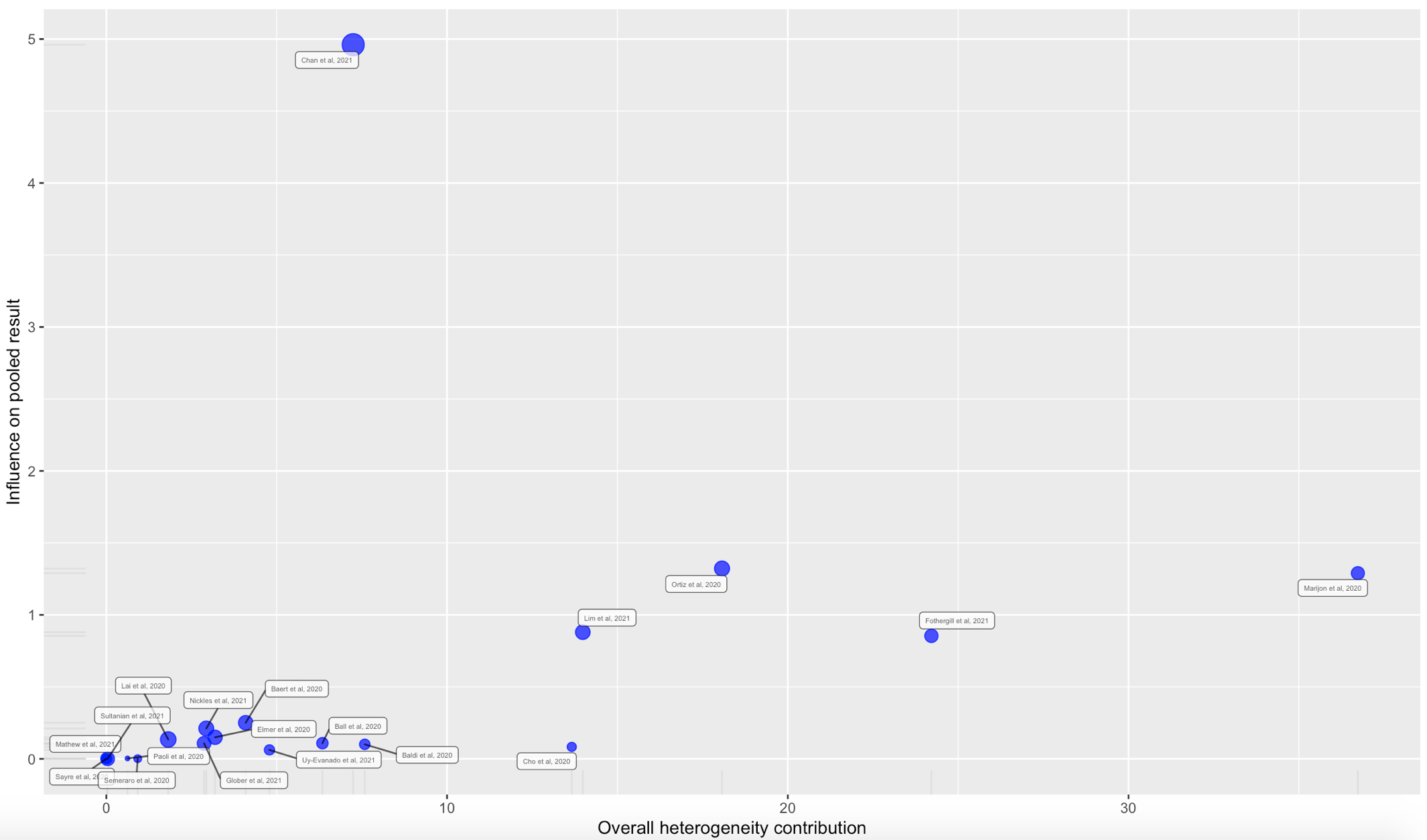


R Core Team (2021). R: A language and environment for statistical computing. R Foundation for Statistical Computing, Vienna, Austria. URL https://www.R-project.org/.

**Supplemental Figure 10. Leave-One-Out Analysis for Bystander Cardiopulmonary Resuscitation**


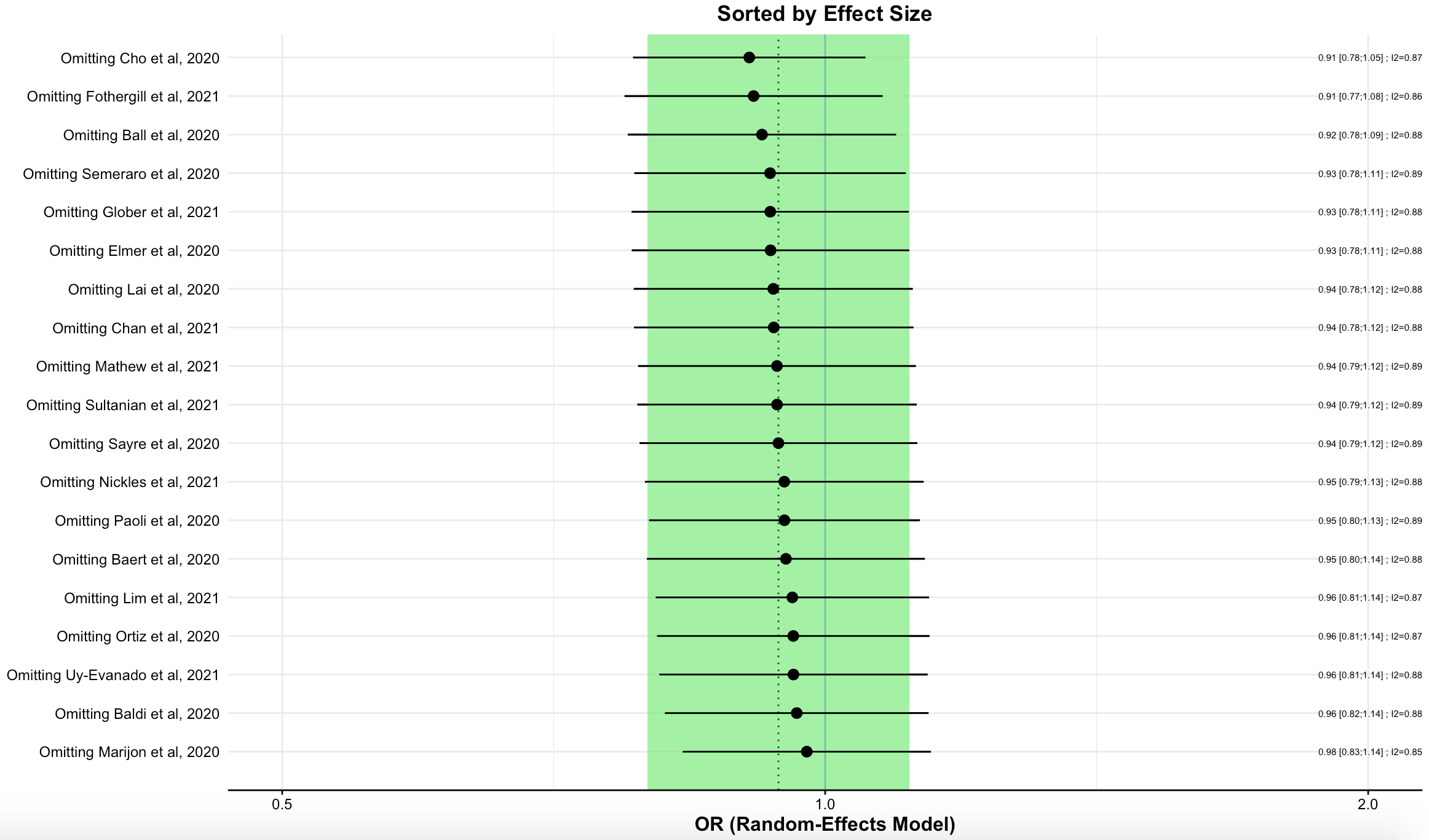


R Core Team (2021). R: A language and environment for statistical computing. R Foundation for Statistical Computing, Vienna, Austria. URL https://www.R-project.org/.

**Supplemental Figure 11. Influential Diagnostic Plot for Automatic External Defibrillator Use**

**
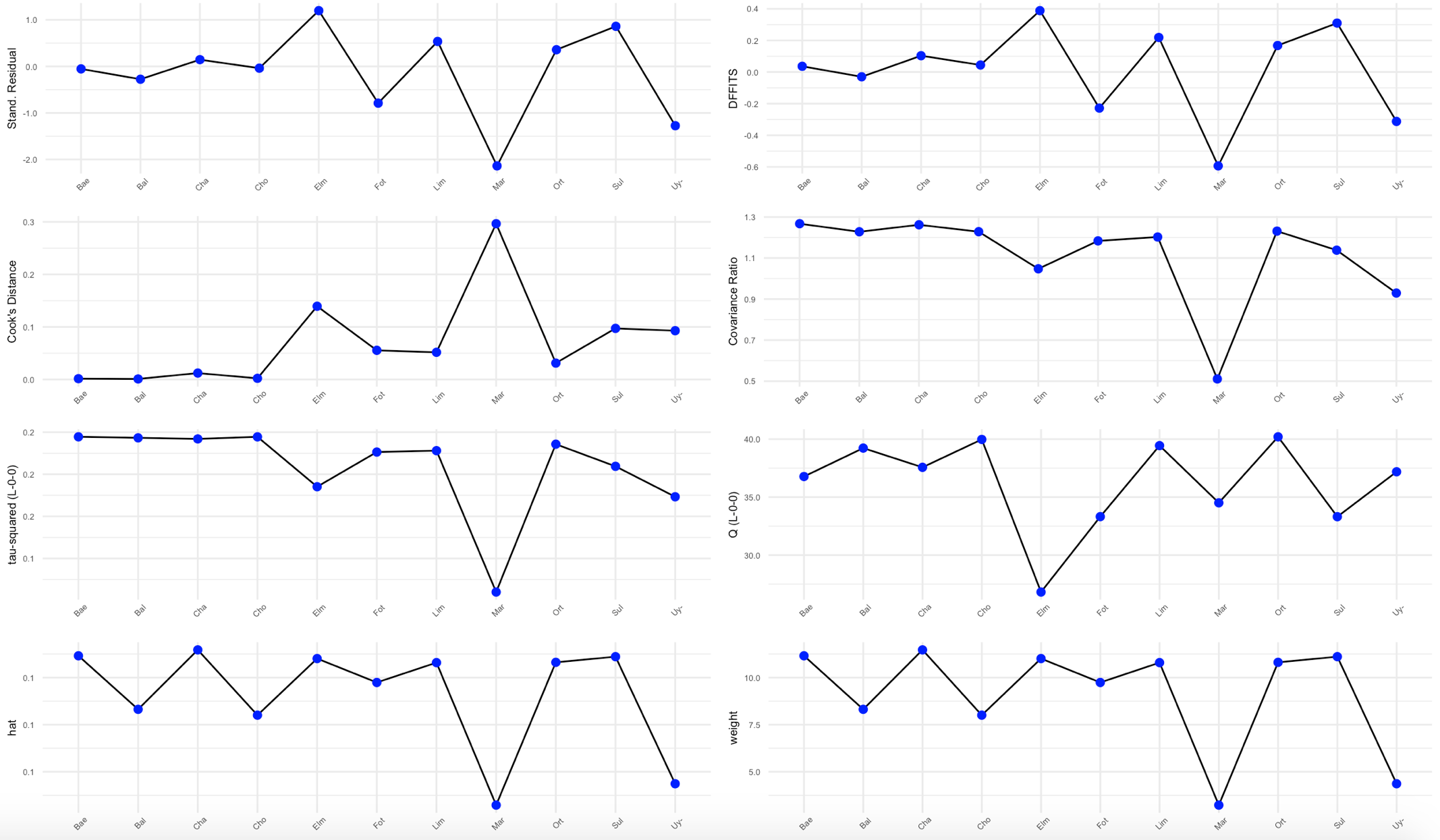
**

R Core Team (2021). R: A language and environment for statistical computing. R Foundation for Statistical Computing, Vienna, Austria. URL https://www.R-project.org/.

**Supplemental Figure 12. Baujat Plot for Automatic External Defibrillator Use**


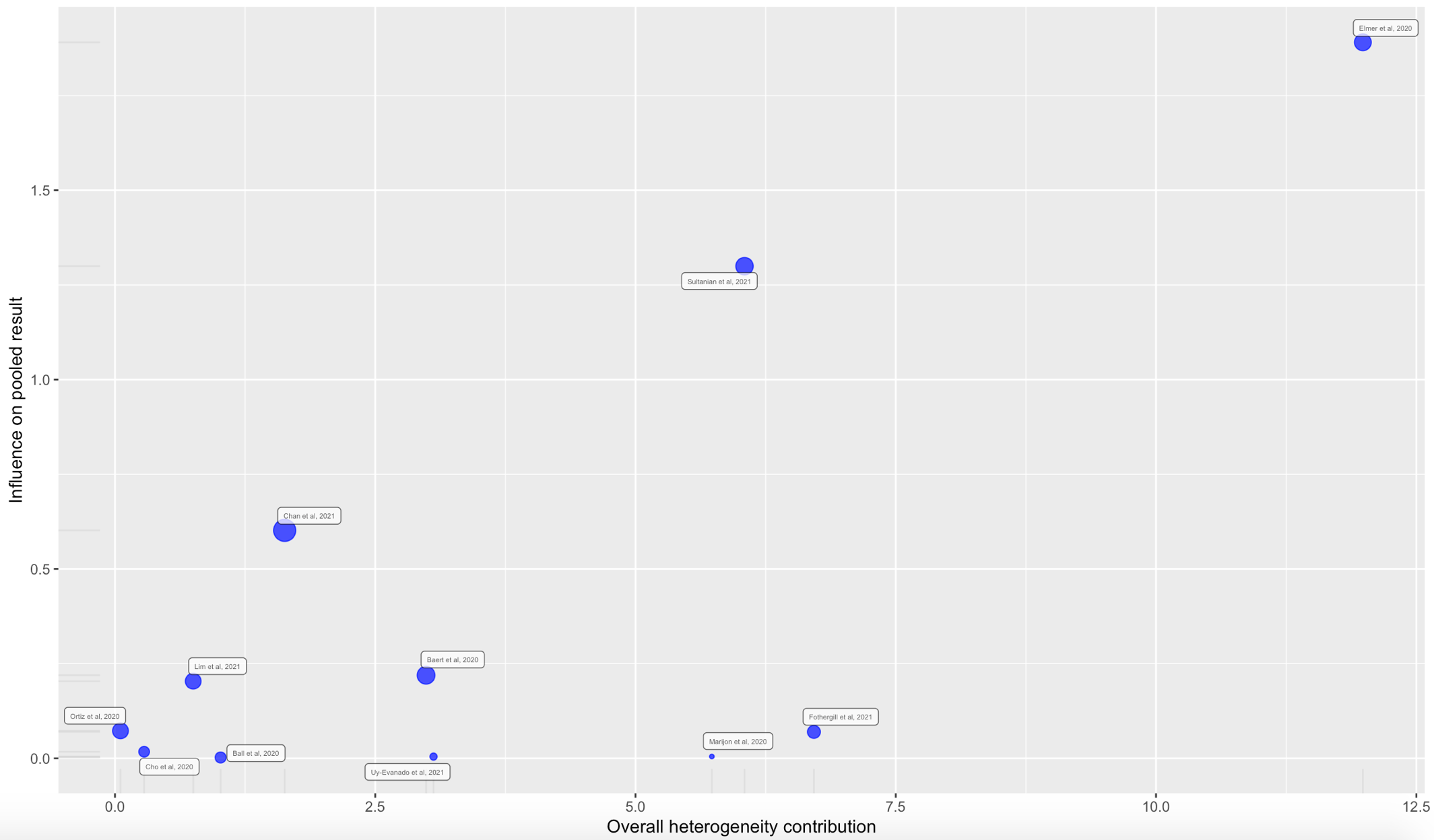


R Core Team (2021). R: A language and environment for statistical computing. R Foundation for Statistical Computing, Vienna, Austria. URL https://www.R-project.org/.

**Supplemental Figure 13. Leave-One-Out Analysis for Automatic External Defibrillator Use**


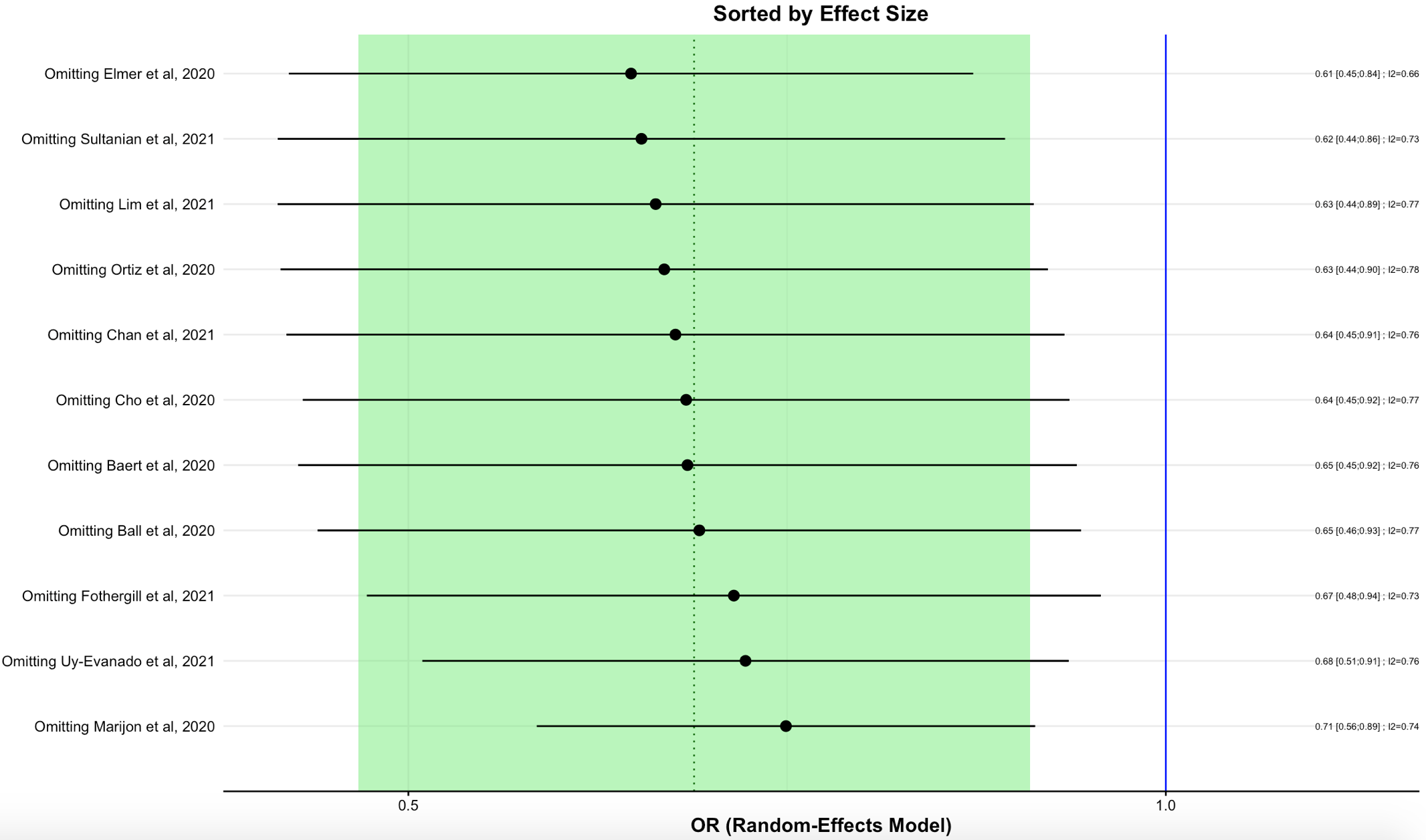


R Core Team (2021). R: A language and environment for statistical computing. R Foundation for Statistical Computing, Vienna, Austria. URL https://www.R-project.org/.

**Supplemental Figure 14. Influential Diagnostic Plot for Emergency Medical Services Resuscitation Attempted**


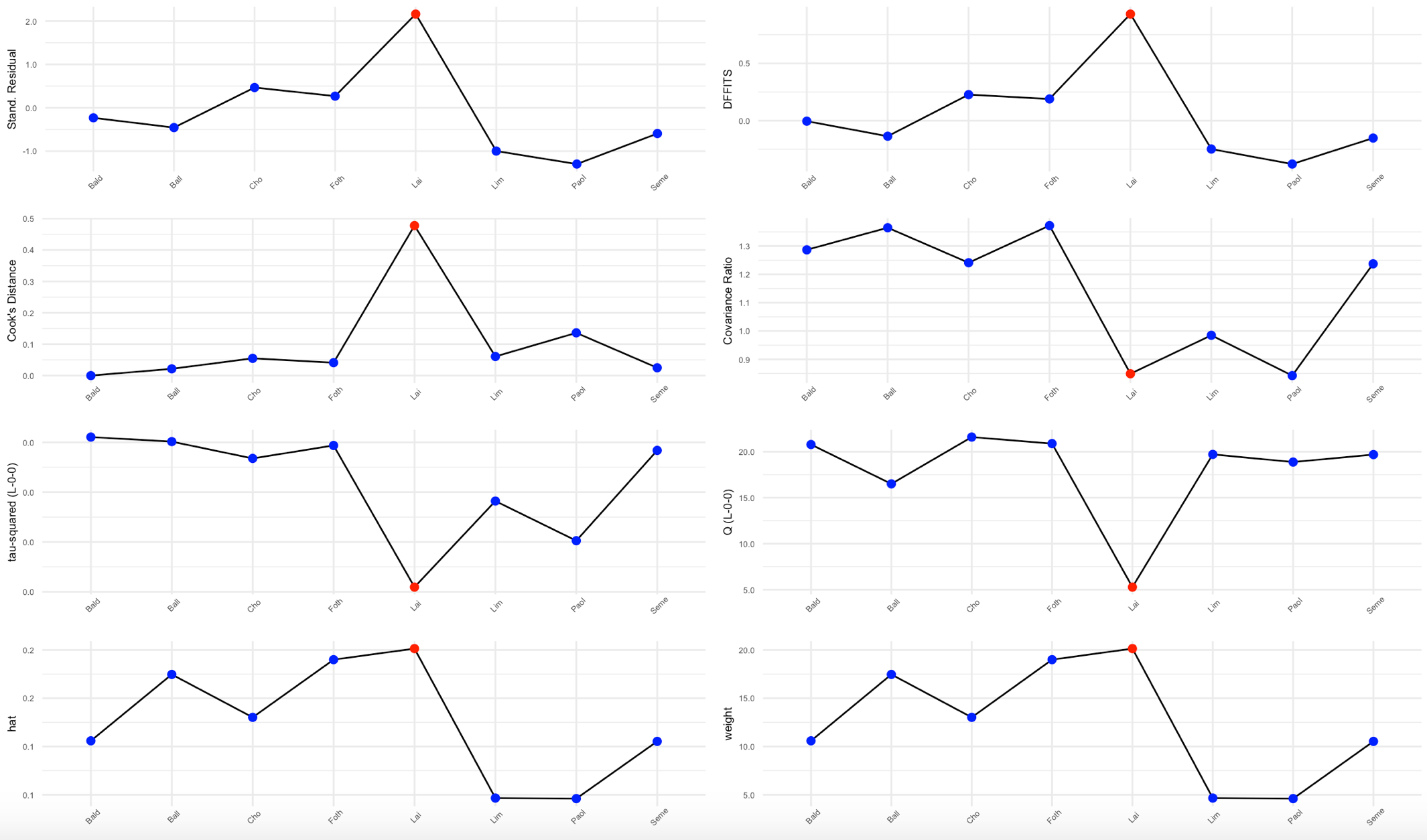


R Core Team (2021). R: A language and environment for statistical computing. R Foundation for Statistical Computing, Vienna, Austria. URL https://www.R-project.org/.

**Supplemental Figure 15. Baujat Plot for Emergency Medical Services Resuscitation Attempted**


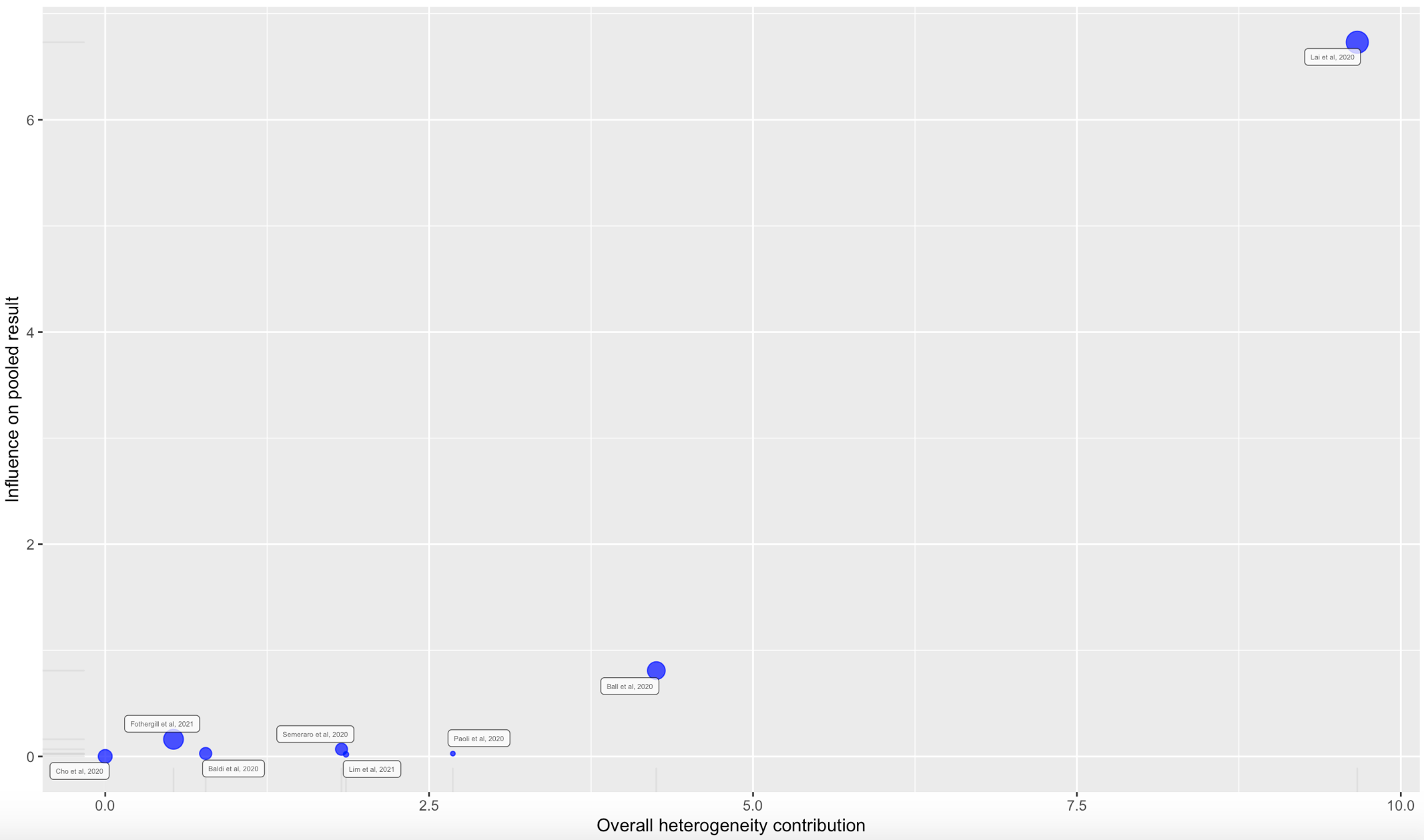


R Core Team (2021). R: A language and environment for statistical computing. R Foundation for Statistical Computing, Vienna, Austria. URL https://www.R-project.org/.

**Supplemental Figure 16. Leave-One-Out Analysis for Emergency Medical Services Resuscitation Attempted**


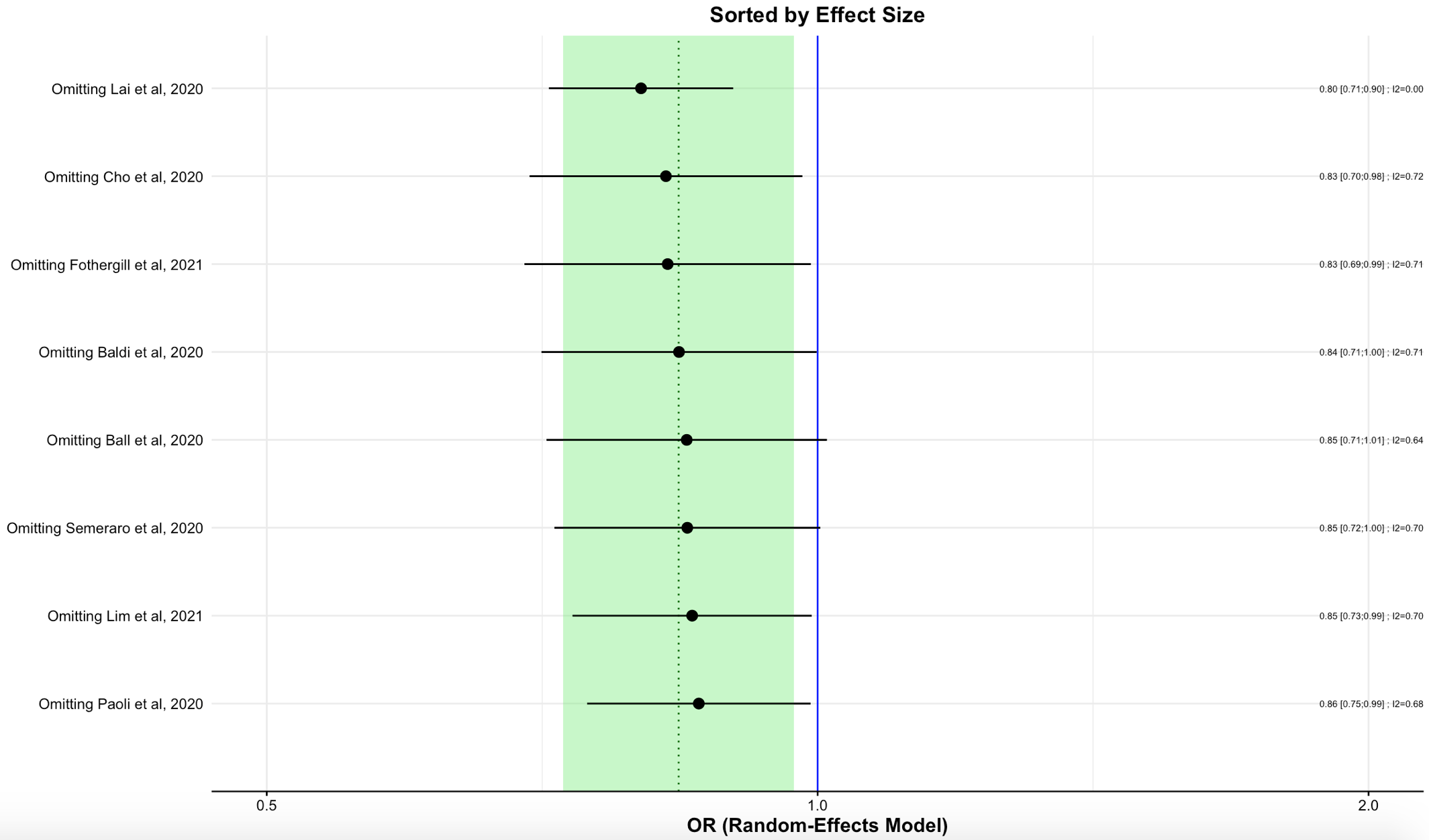


R Core Team (2021). R: A language and environment for statistical computing. R Foundation for Statistical Computing, Vienna, Austria. URL https://www.R-project.org/.

**Supplemental Figure 17. Influential Diagnostic Plot for Emergency Medical Services Call to Arrival Time**


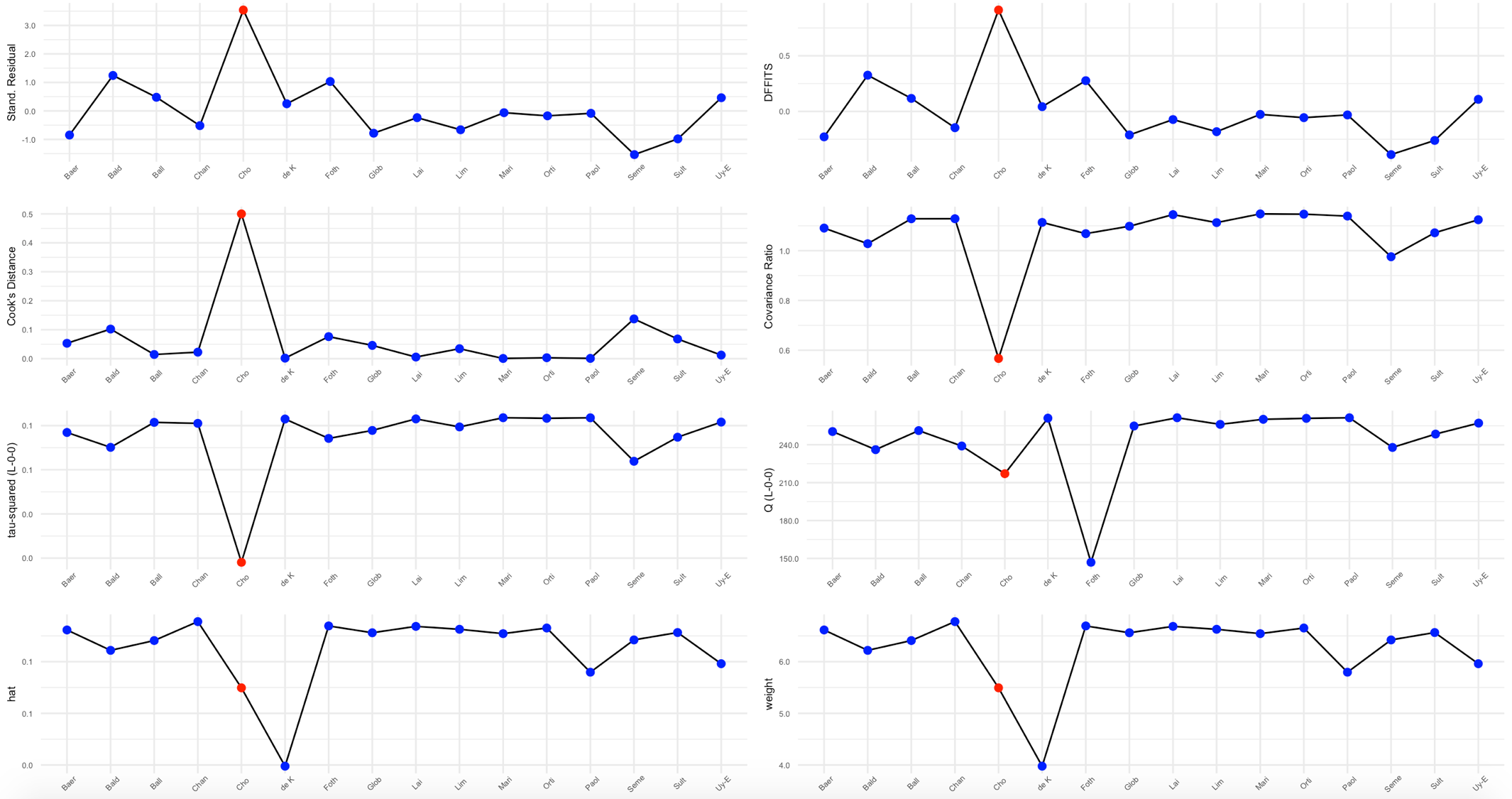


R Core Team (2021). R: A language and environment for statistical computing. R Foundation for Statistical Computing, Vienna, Austria. URL https://www.R-project.org/.

**Supplemental Figure 18. Baujat Plot for Emergency Medical Services Call to Arrival Time**


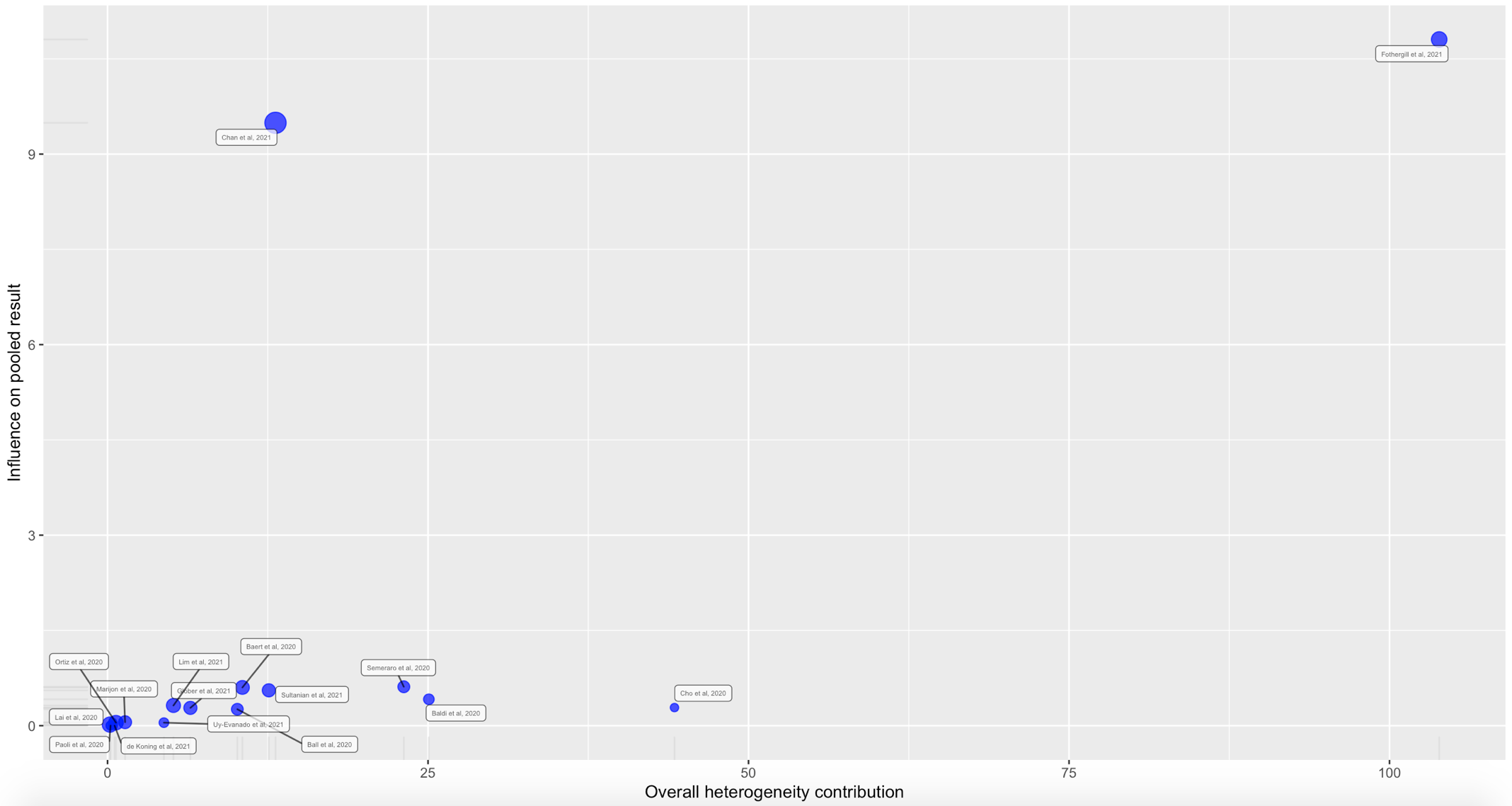


R Core Team (2021). R: A language and environment for statistical computing. R Foundation for Statistical Computing, Vienna, Austria. URL https://www.R-project.org/.

**Supplemental Figure 19. Leave-One-Out Analysis for Emergency Medical Services Call to Arrival Time**


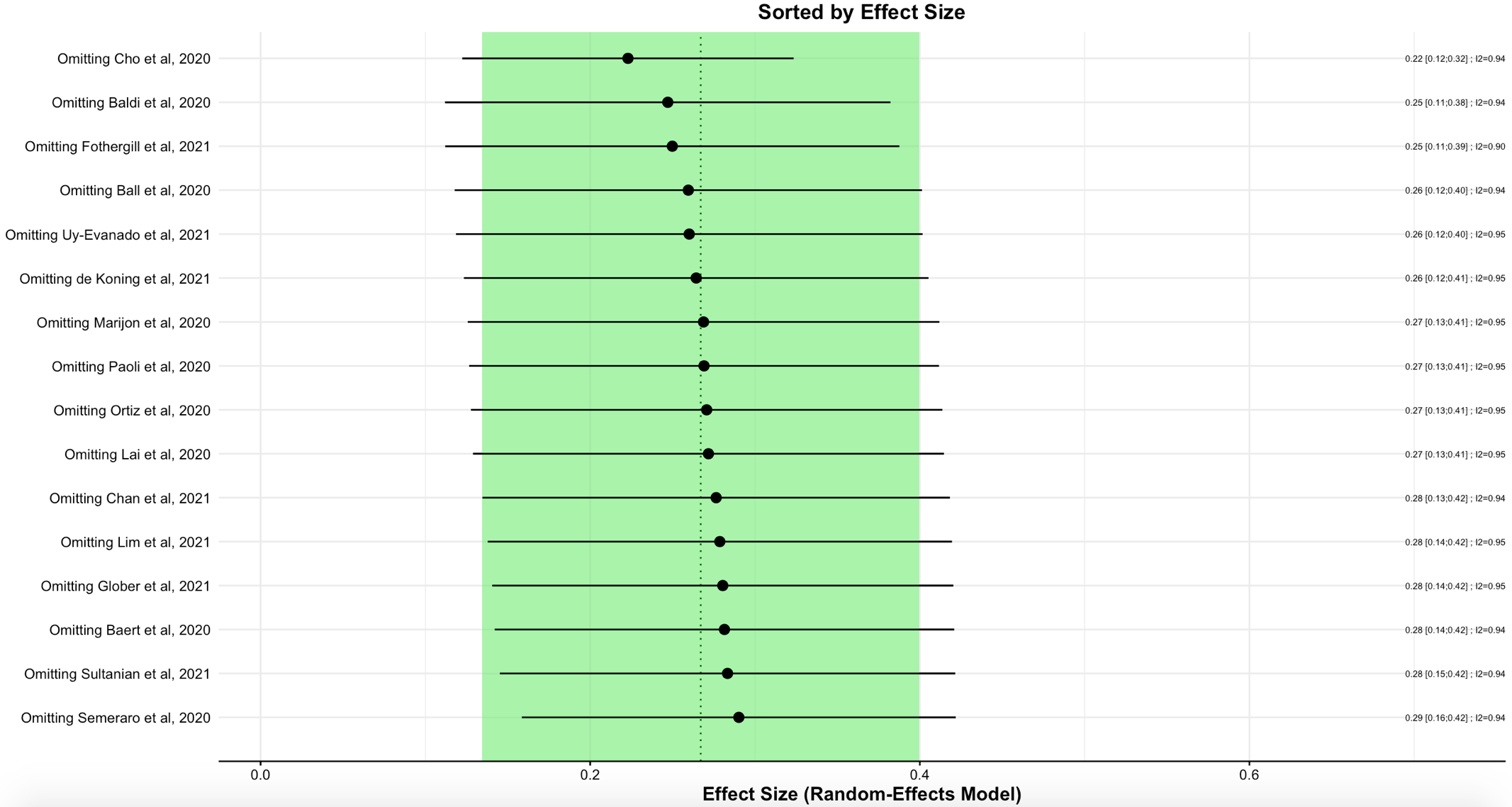


R Core Team (2021). R: A language and environment for statistical computing. R Foundation for Statistical Computing, Vienna, Austria. URL https://www.R-project.org/.

**Supplemental Figure 20. Influential Diagnostic Plot for Resuscitation Duration**

**
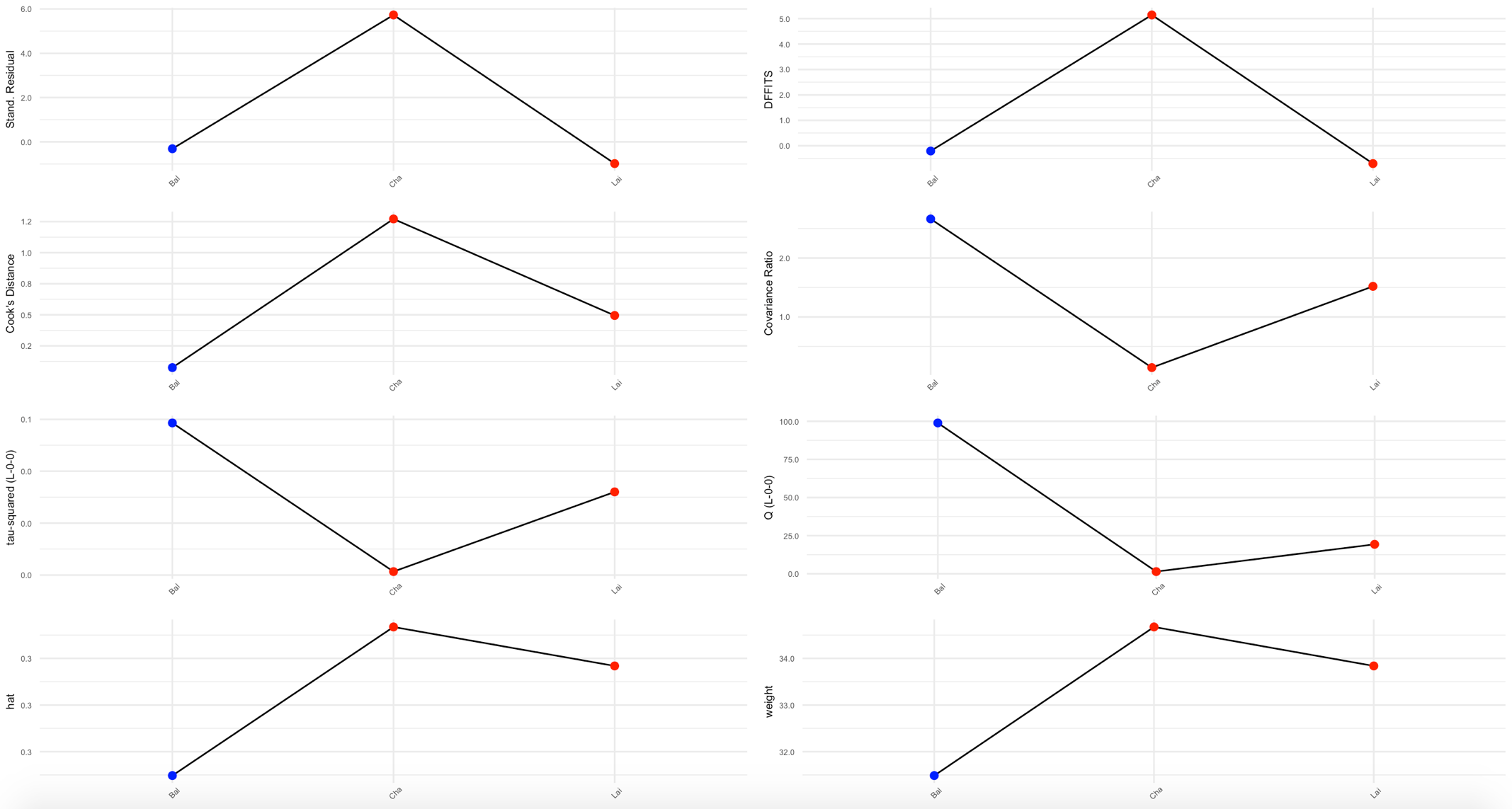
**

R Core Team (2021). R: A language and environment for statistical computing. R Foundation for Statistical Computing, Vienna, Austria. URL https://www.R-project.org/.

**Supplemental Figure 21. Baujat Plot for Resuscitation Duration**


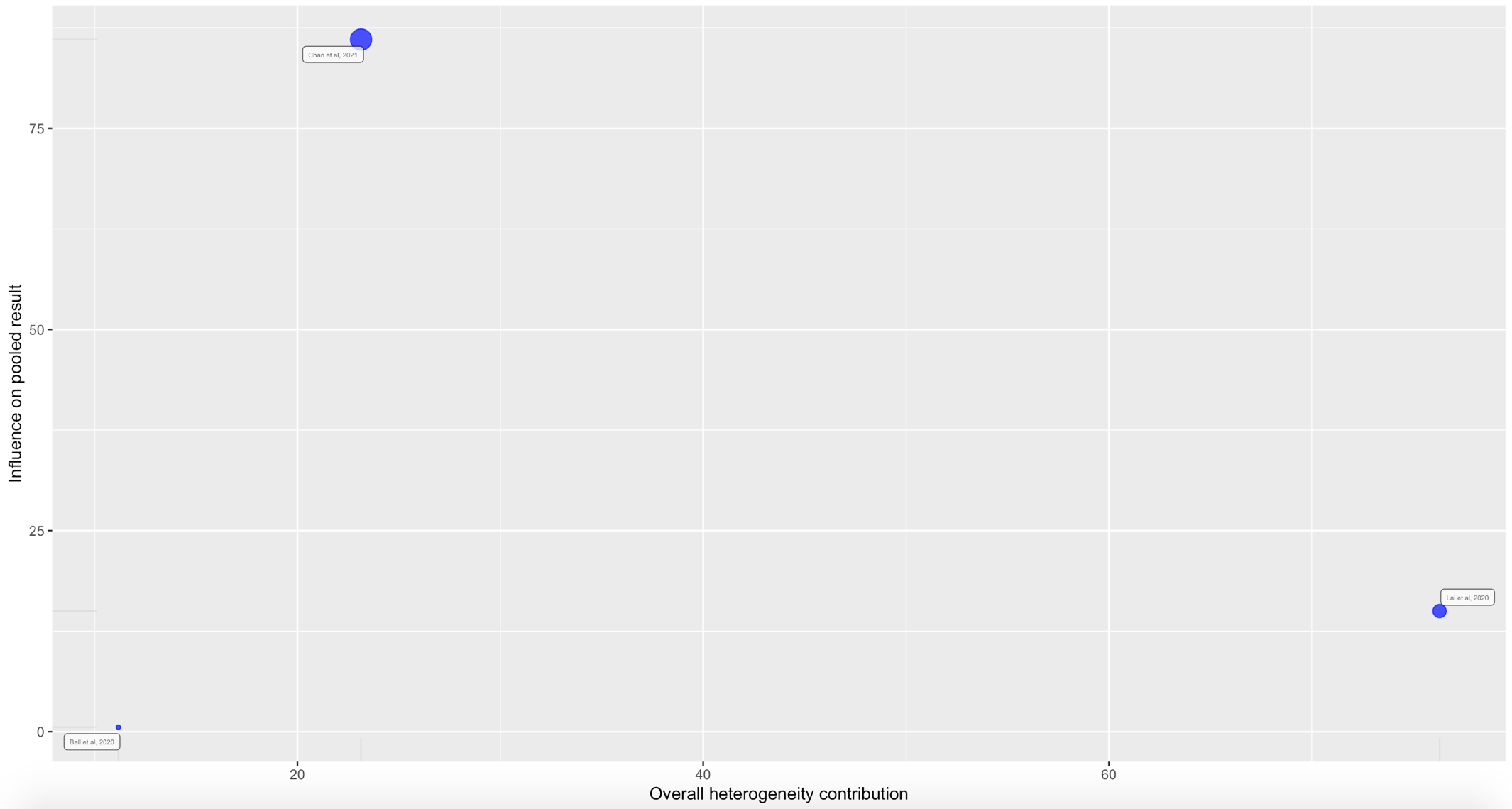


R Core Team (2021). R: A language and environment for statistical computing. R Foundation for Statistical Computing, Vienna, Austria. URL https://www.R-project.org/.

**Supplemental Figure 22. Leave-One-Out Analysis for Resuscitation Duration**


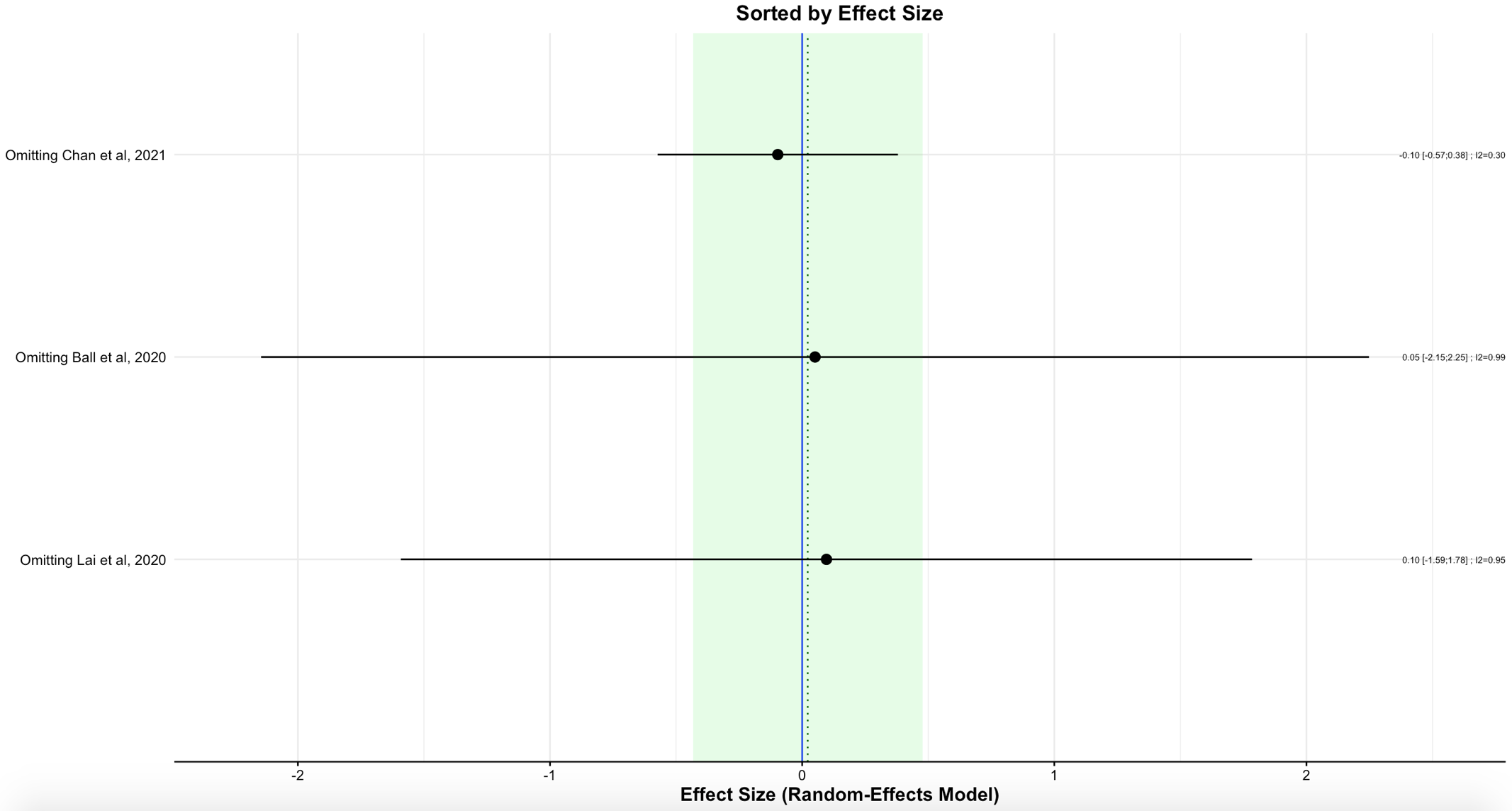


R Core Team (2021). R: A language and environment for statistical computing. R Foundation for Statistical Computing, Vienna, Austria. URL https://www.R-project.org/.

**Supplemental Figure 23. Influential Diagnostic Plot for Endotracheal Intubation**


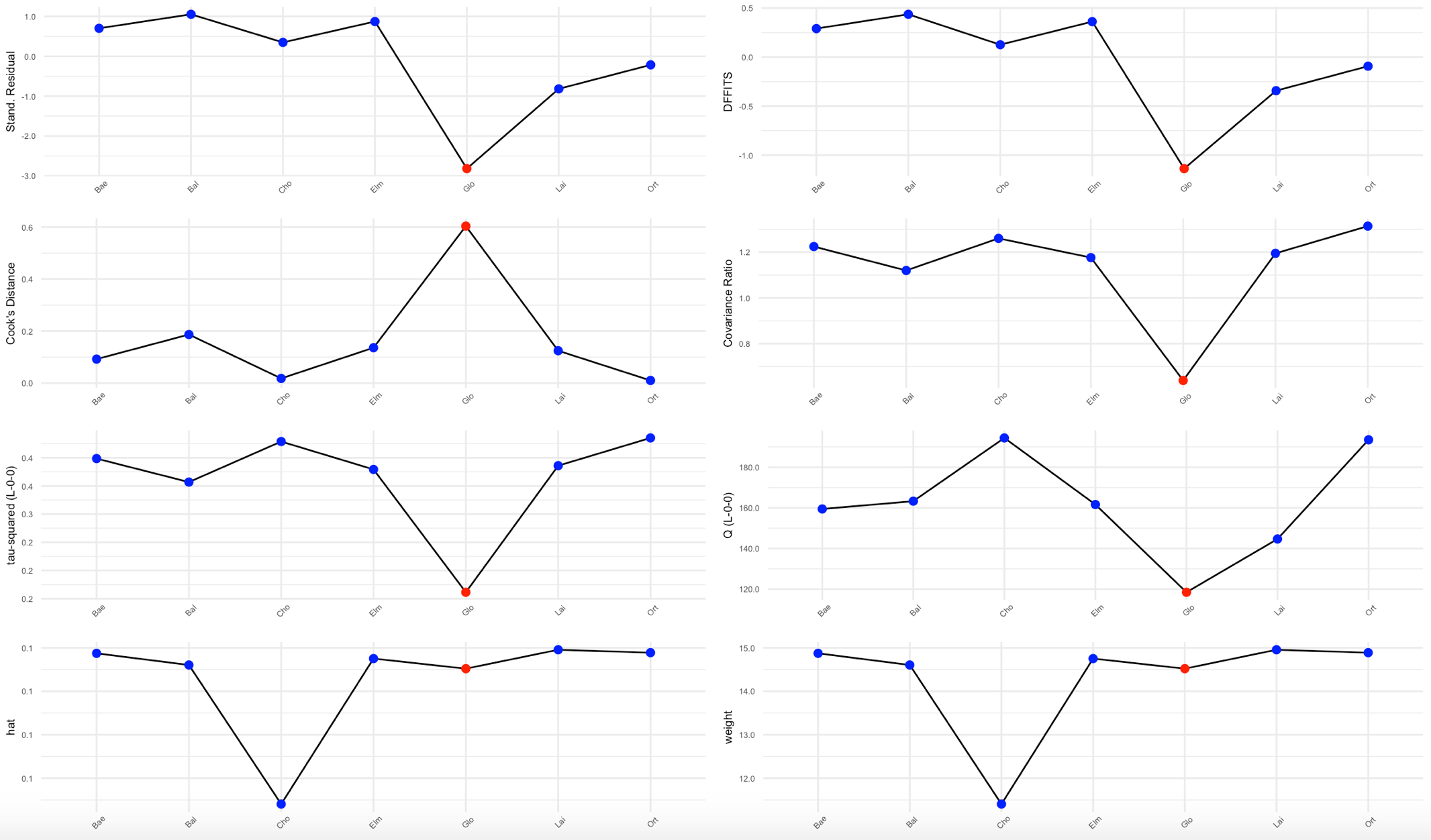


R Core Team (2021). R: A language and environment for statistical computing. R Foundation for Statistical Computing, Vienna, Austria. URL https://www.R-project.org/.

**Supplemental Figure 24. Baujat Plot for Endotracheal Intubation**


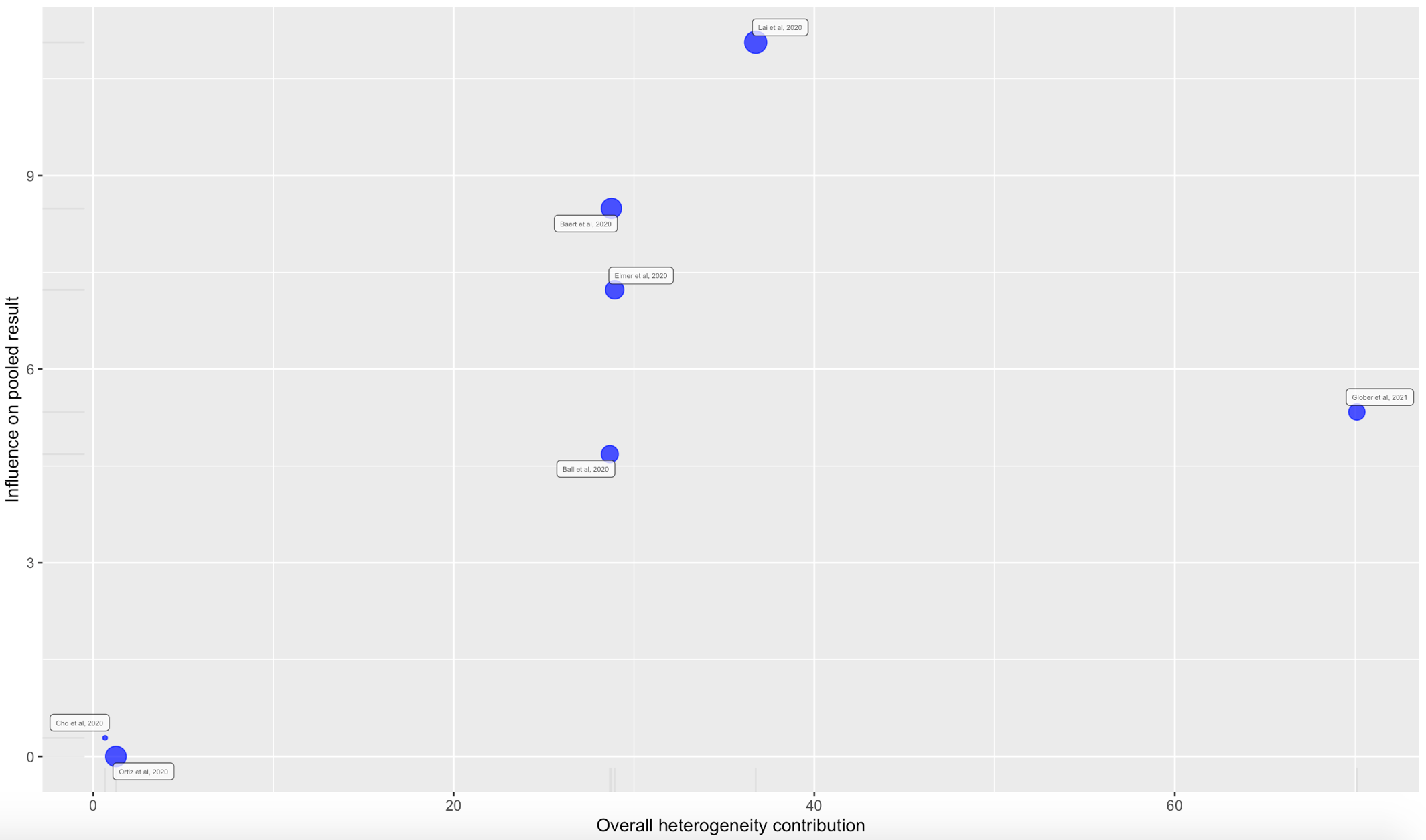


R Core Team (2021). R: A language and environment for statistical computing. R Foundation for Statistical Computing, Vienna, Austria. URL https://www.R-project.org/.

**Supplemental Figure 25. Leave-One-Out Analysis for Endotracheal Intubation**


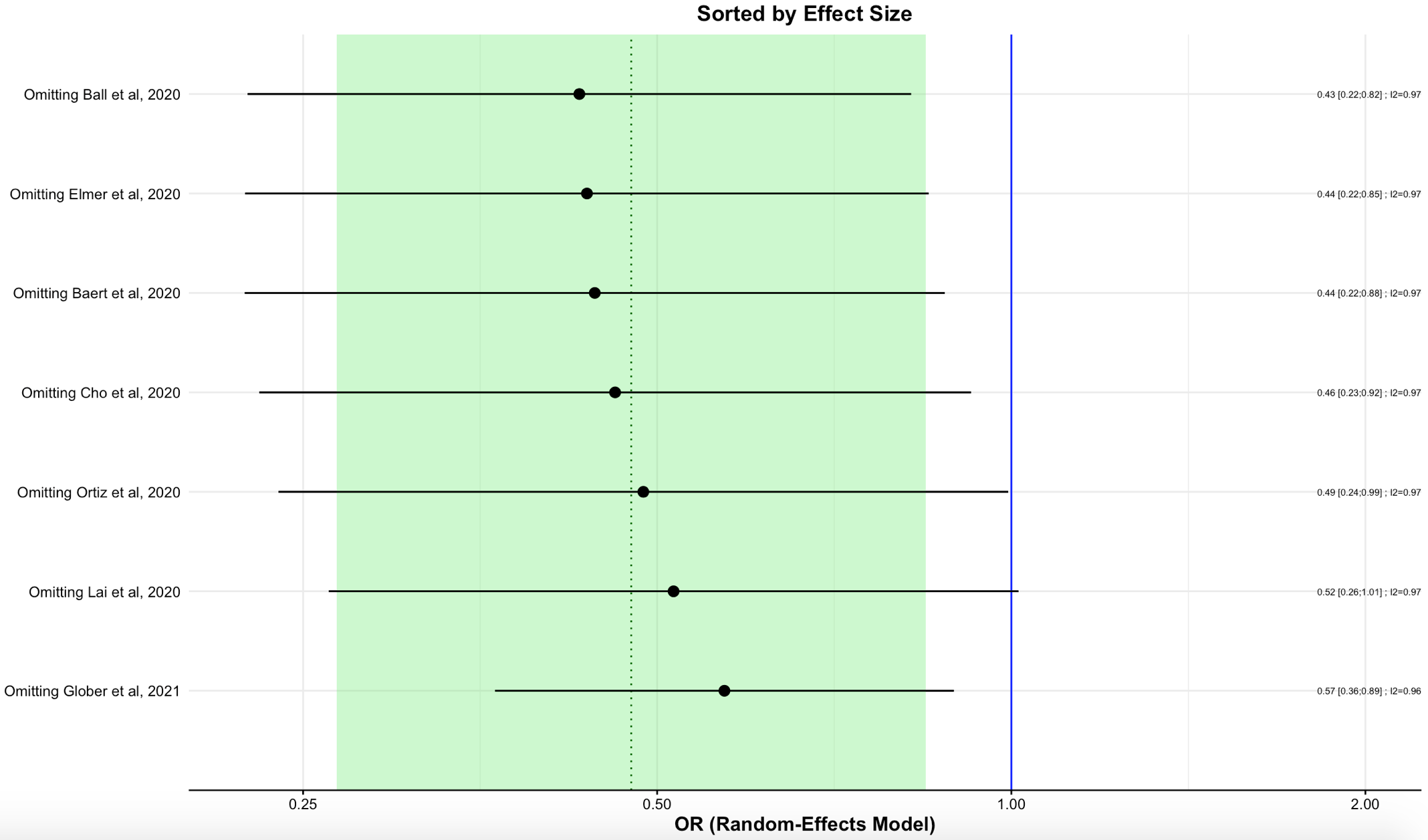


R Core Team (2021). R: A language and environment for statistical computing. R Foundation for Statistical Computing, Vienna, Austria. URL https://www.R-project.org/.

**Supplemental Figure 26. Influential Diagnostic Plot for Supraglottic Airway**


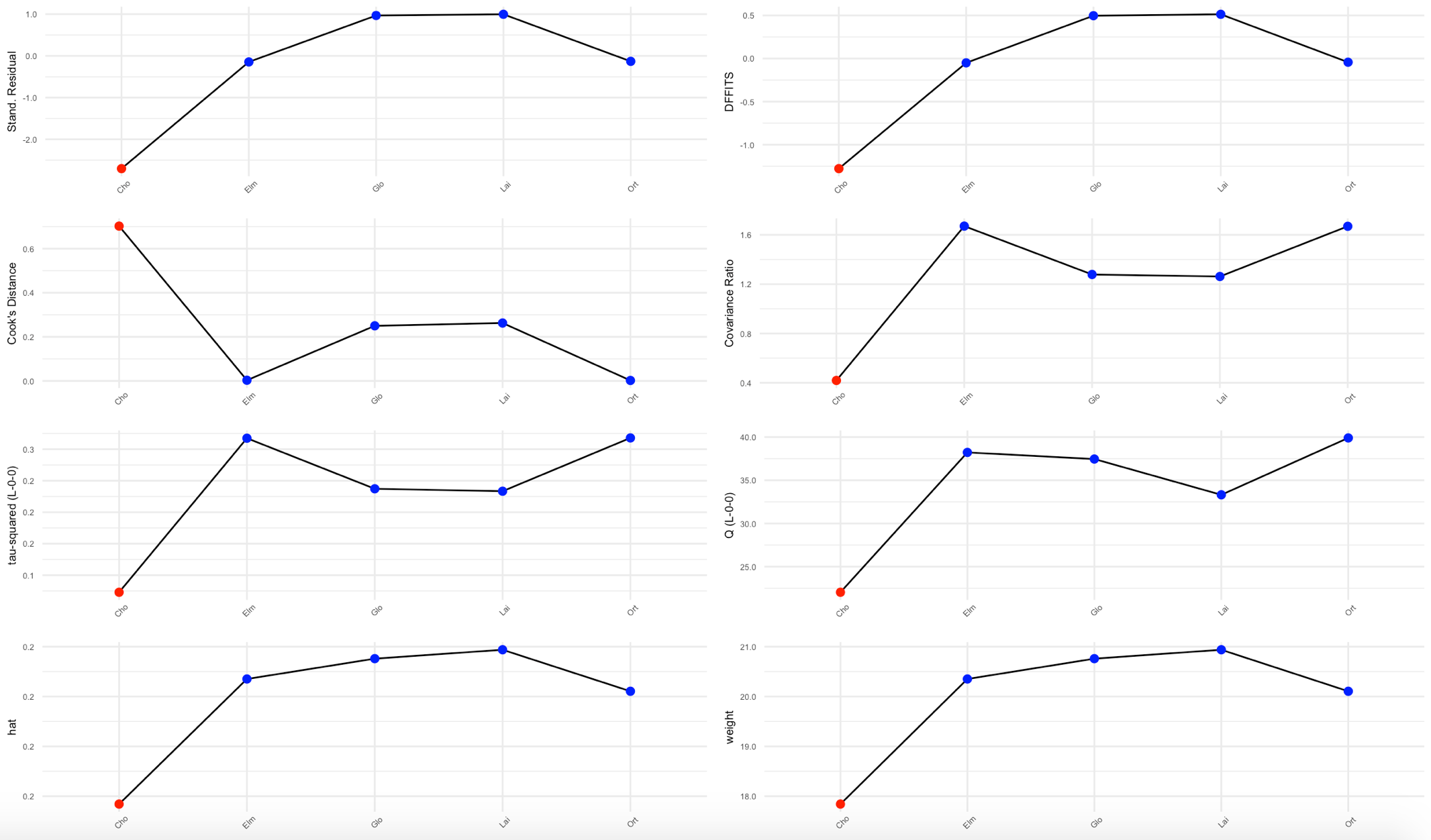


R Core Team (2021). R: A language and environment for statistical computing. R Foundation for Statistical Computing, Vienna, Austria. URL https://www.R-project.org/.

**Supplemental Figure 27. Baujat Plot for Supraglottic Airway**

**
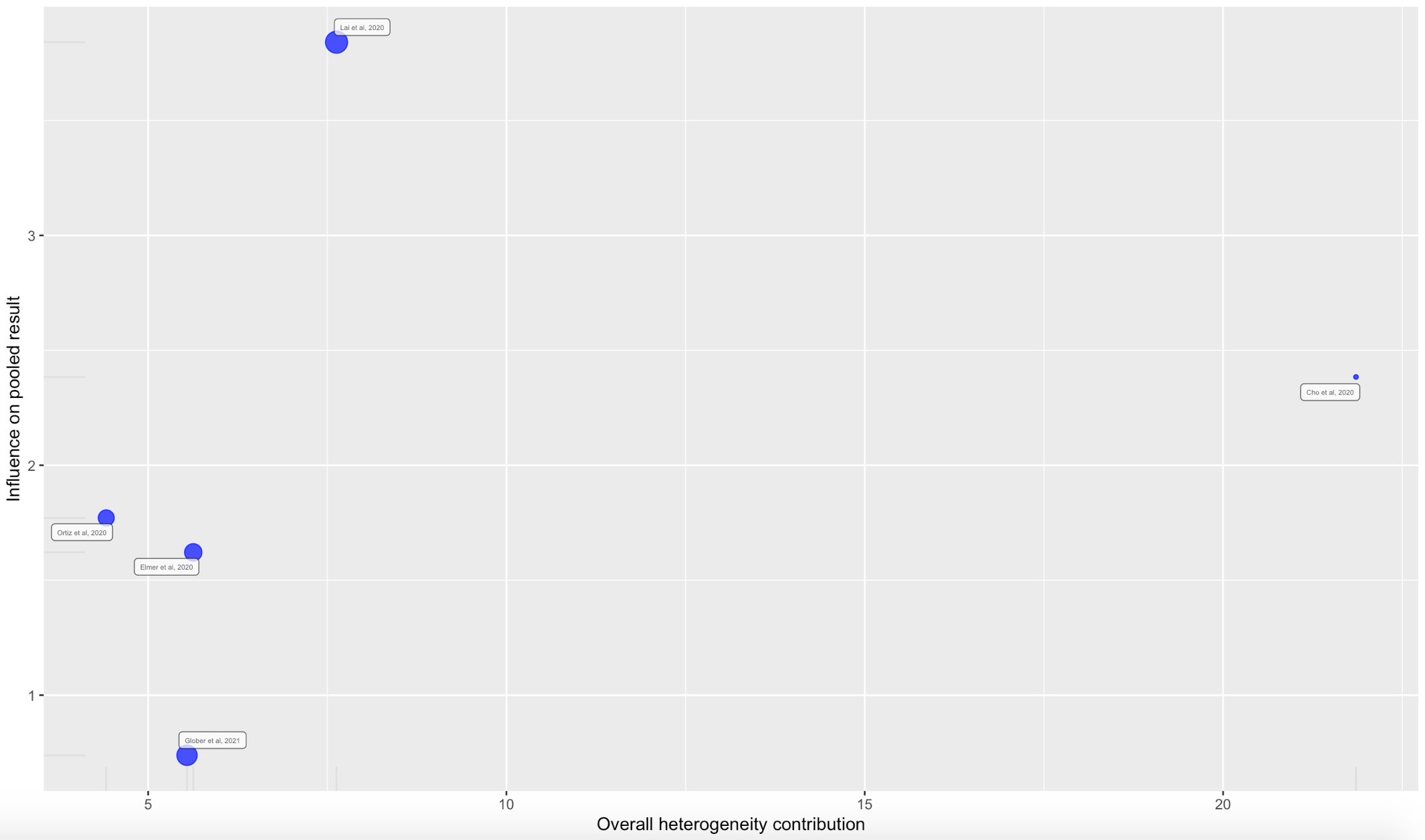
**

R Core Team (2021). R: A language and environment for statistical computing. R Foundation for Statistical Computing, Vienna, Austria. URL https://www.R-project.org/.

**Supplemental Figure 28. Leave-One-Out Analysis for Supraglottic Airway**


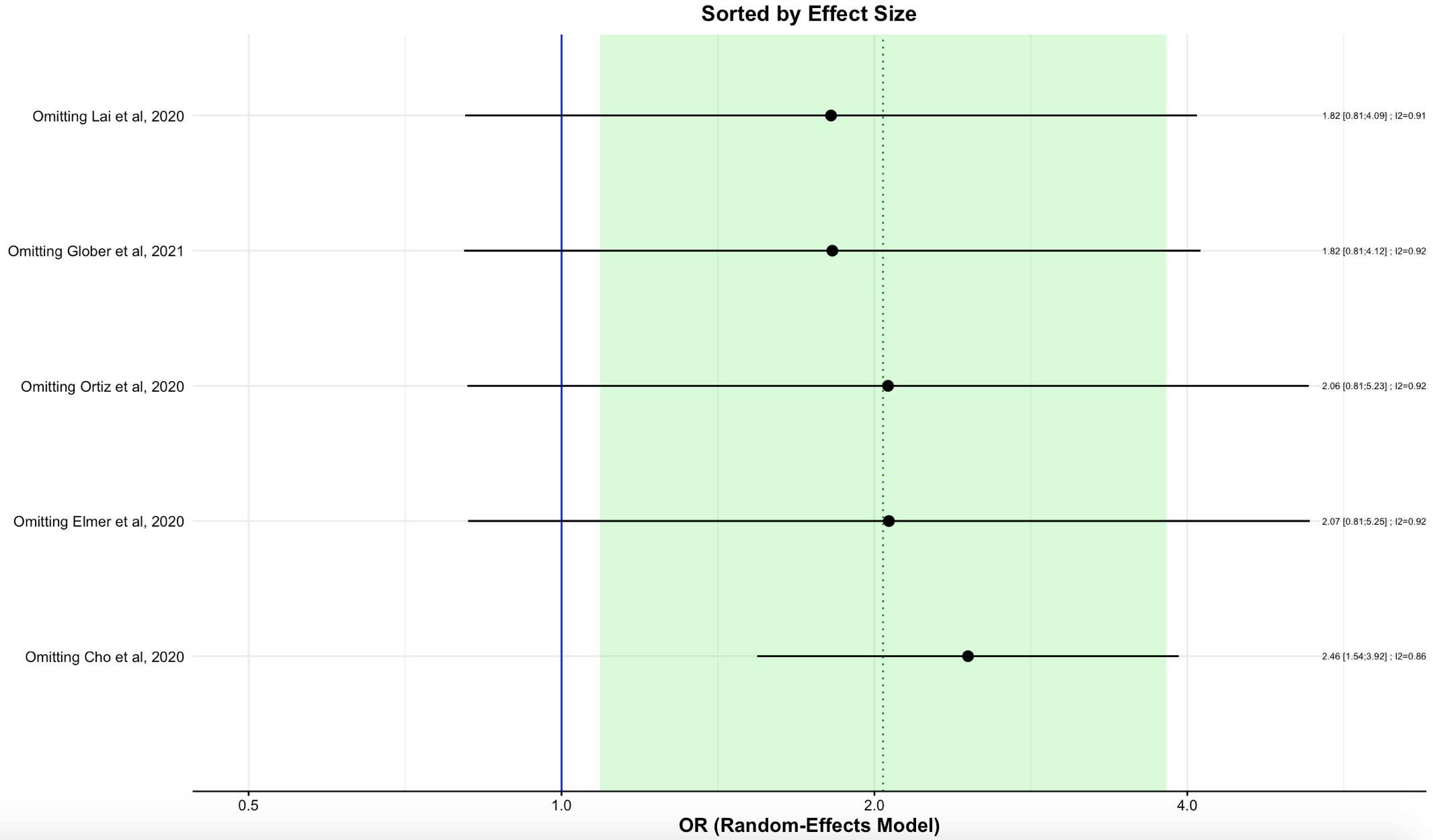


R Core Team (2021). R: A language and environment for statistical computing. R Foundation for Statistical Computing, Vienna, Austria. URL https://www.R-project.org/.

**Supplemental Figure 29. Influential Diagnostic Plot for Amiodarone**


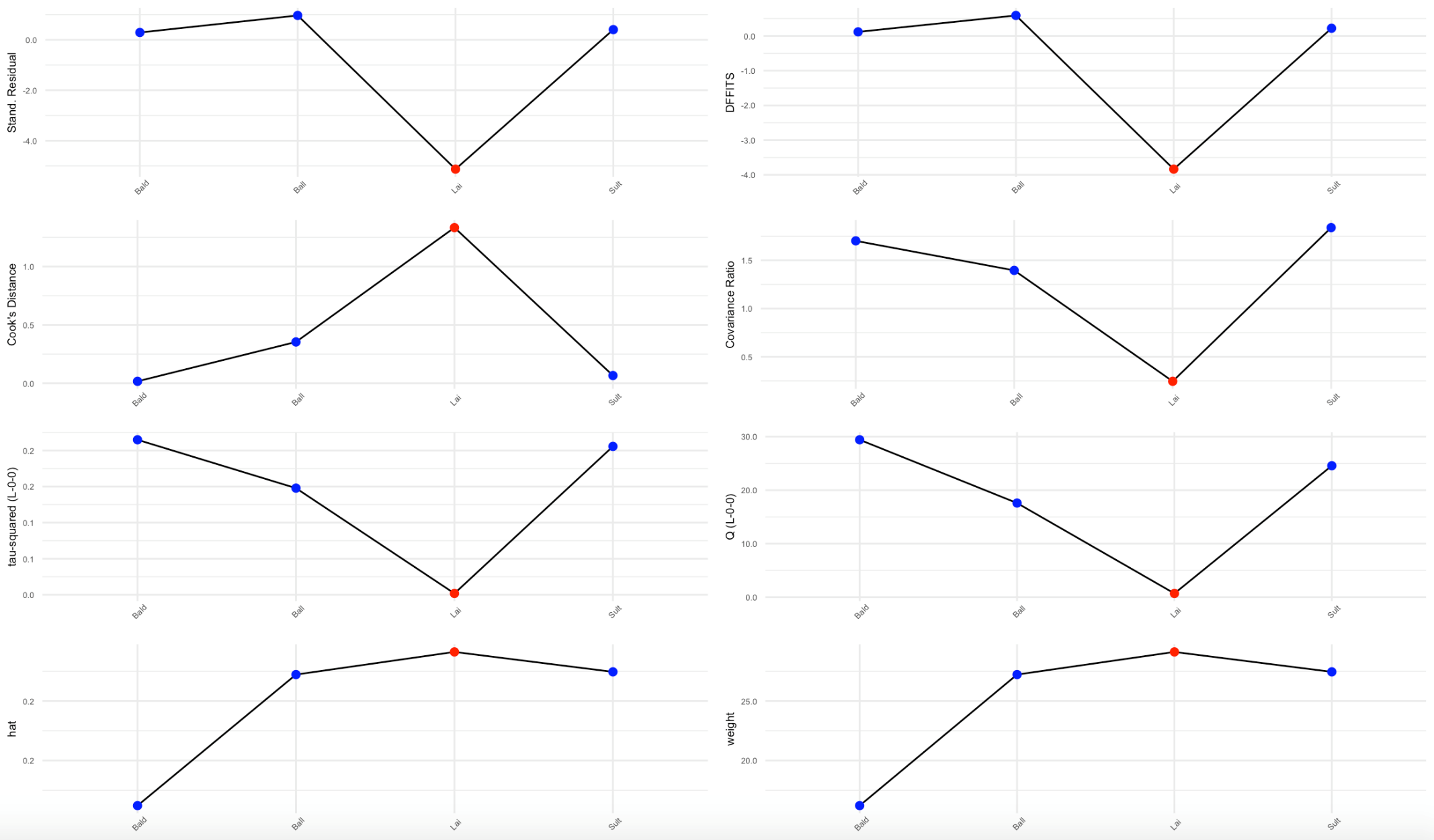


R Core Team (2021). R: A language and environment for statistical computing. R Foundation for Statistical Computing, Vienna, Austria. URL https://www.R-project.org/.

**Supplemental Figure 30. Baujat Plot for Amiodarone**


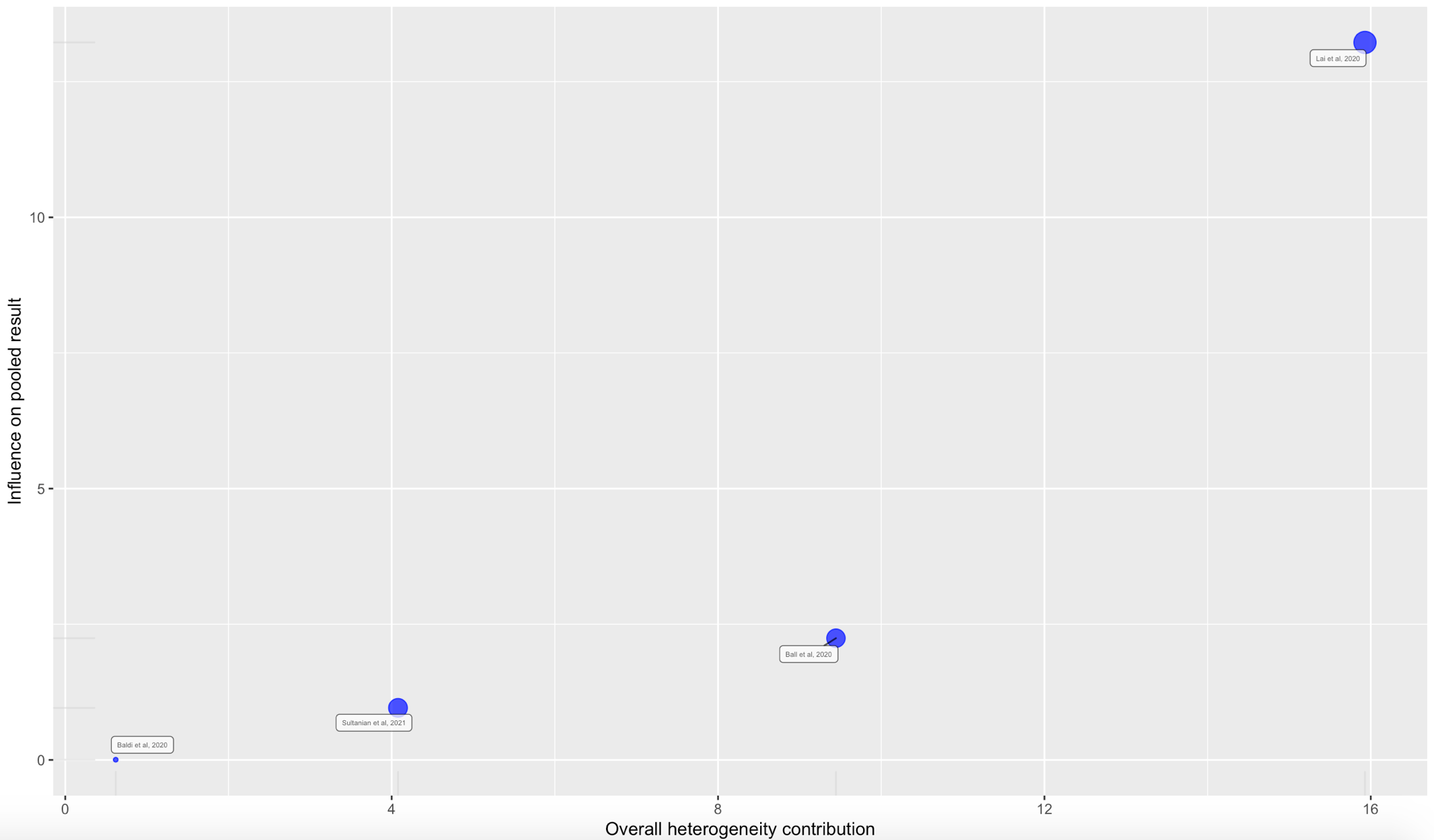


R Core Team (2021). R: A language and environment for statistical computing. R Foundation for Statistical Computing, Vienna, Austria. URL https://www.R-project.org/.

**Supplemental Figure 31. Leave-One-Out Analysis for Amiodarone**


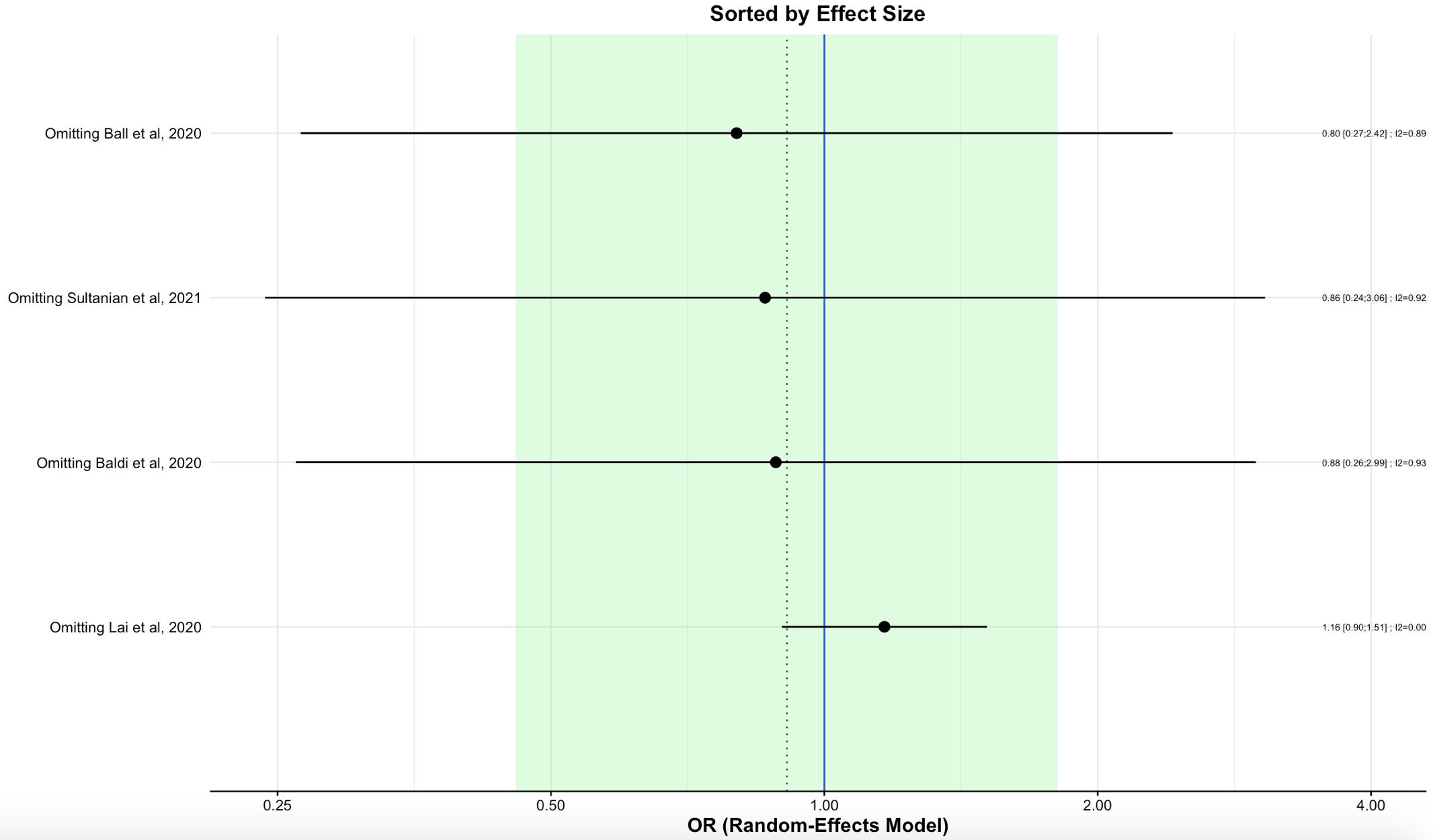


R Core Team (2021). R: A language and environment for statistical computing. R Foundation for Statistical Computing, Vienna, Austria. URL https://www.R-project.org/.

**Supplemental Figure 32. Influential Diagnostic Plot for Epinephrine**


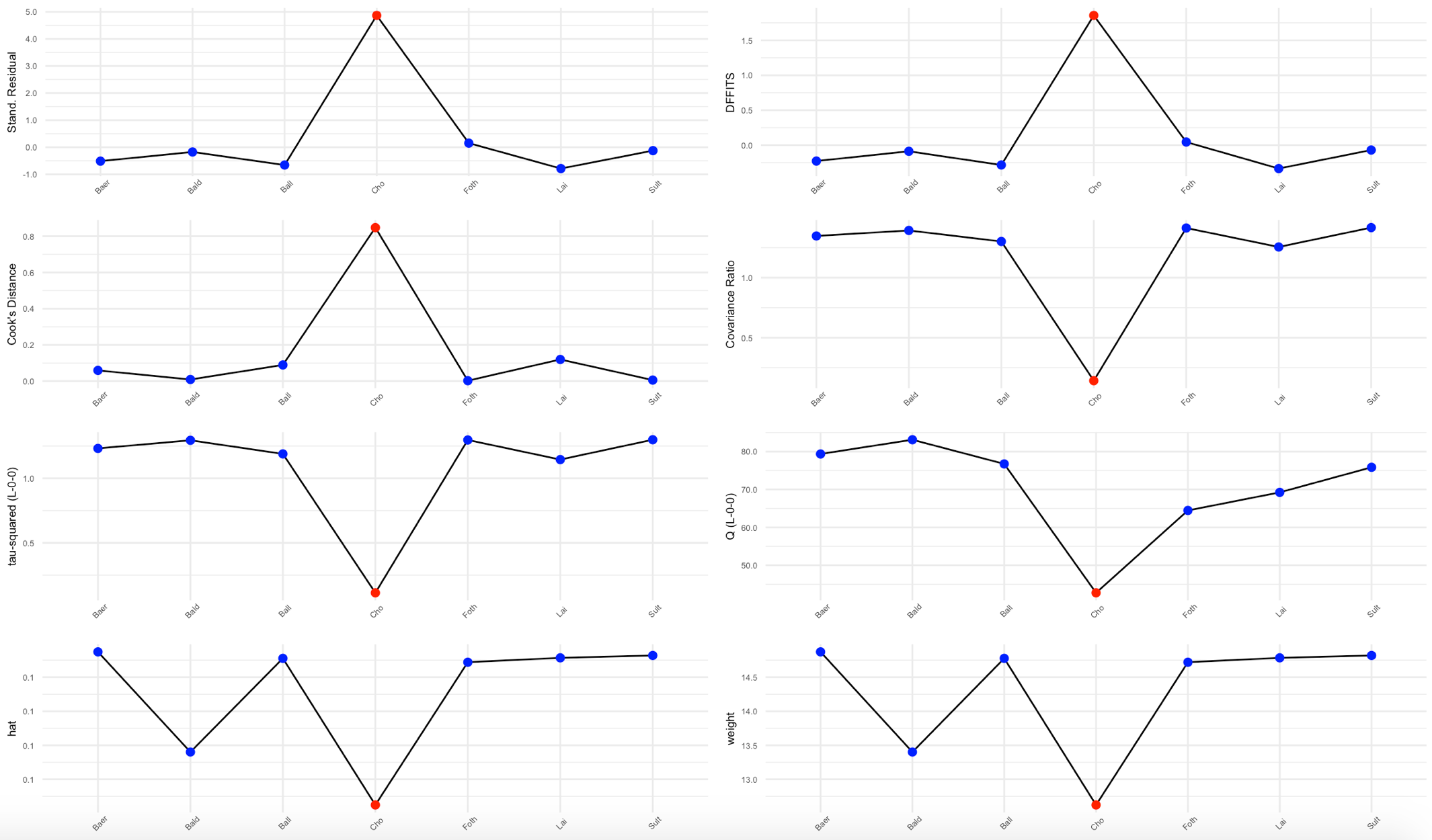


R Core Team (2021). R: A language and environment for statistical computing. R Foundation for Statistical Computing, Vienna, Austria. URL https://www.R-project.org/.

**Supplemental Figure 33. Baujat Plot for Epinephrine**


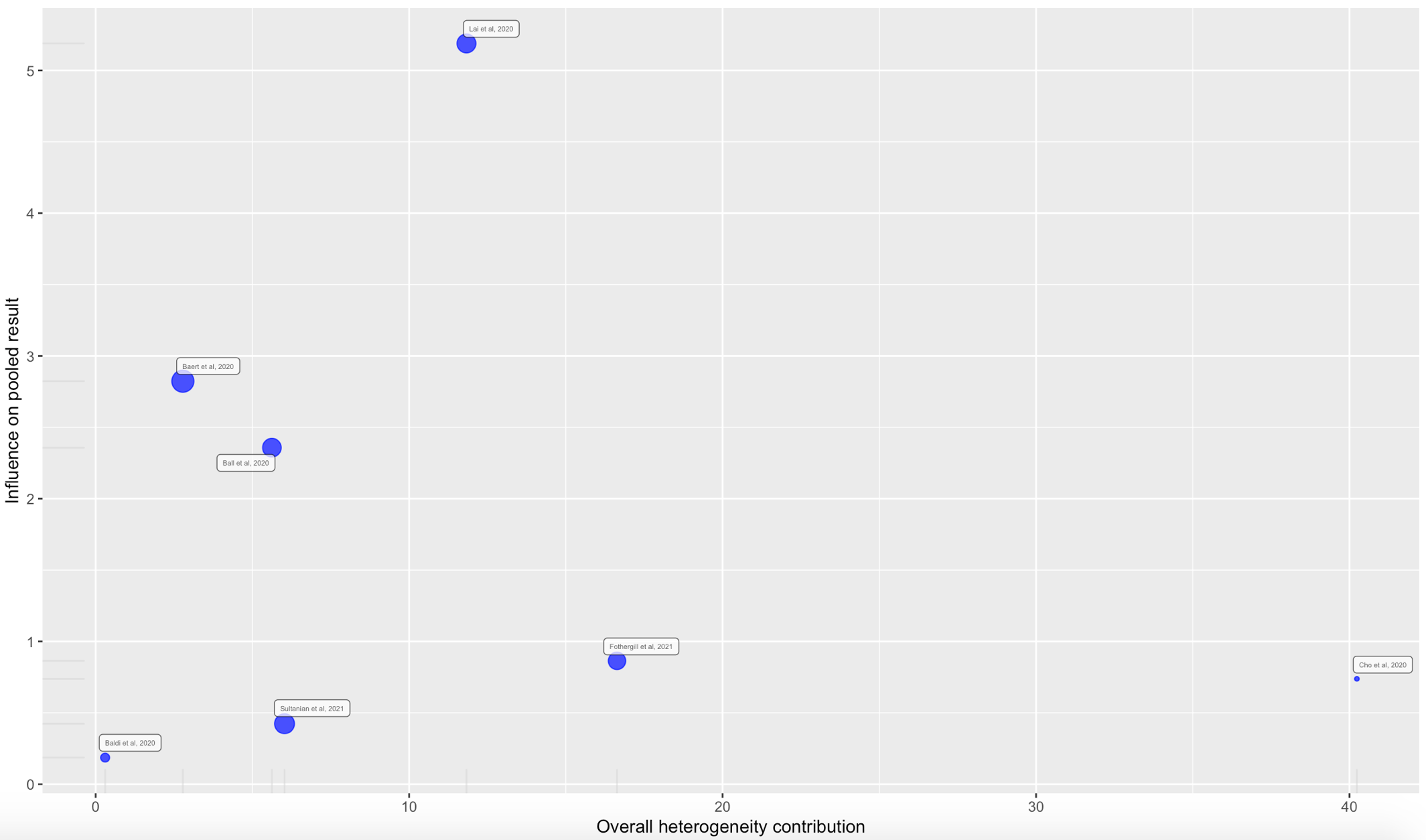


R Core Team (2021). R: A language and environment for statistical computing. R Foundation for Statistical Computing, Vienna, Austria. URL https://www.R-project.org/.

**Supplemental Figure 34. Leave-One-Out Analysis for Epinephrine**


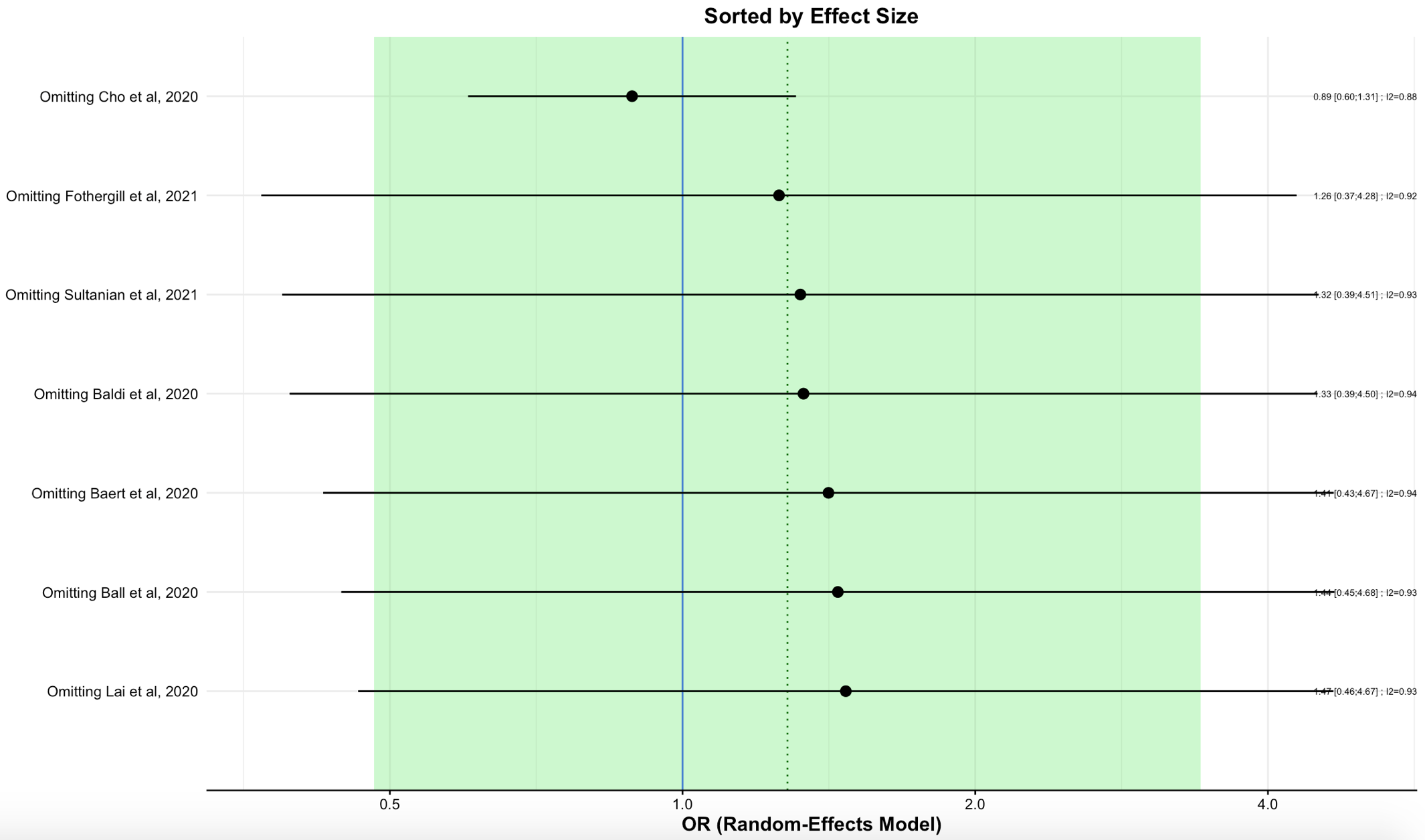


R Core Team (2021). R: A language and environment for statistical computing. R Foundation for Statistical Computing, Vienna, Austria. URL https://www.R-project.org/.

1. **Online Supplemental Data**

**Supplemental Data 1. PRISMA-P 2020 Checklist**

| **Section and Topic** | **Item #** | **Checklist item** | **Location where item is reported** |
| --- | --- | --- | --- |
| **TITLE** | | |  |
| Title | 1 | Identify the report as a systematic review. | 1 |
| **ABSTRACT** | | |  |
| Abstract | 2 | See the PRISMA 2020 for Abstracts checklist. | 2 |
| **INTRODUCTION** | | |  |
| Rationale | 3 | Describe the rationale for the review in the context of existing knowledge. | 3-4 |
| Objectives | 4 | Provide an explicit statement of the objective(s) or question(s) the review addresses. | 3-4 |
| **METHODS** | | |  |
| Eligibility criteria | 5 | Specify the inclusion and exclusion criteria for the review and how studies were grouped for the syntheses. | 5-6 |
| Information sources | 6 | Specify all databases, registers, websites, organisations, reference lists and other sources searched or consulted to identify studies. Specify the date when each source was last searched or consulted. | 5 |
| Search strategy | 7 | Present the full search strategies for all databases, registers and websites, including any filters and limits used. | 5 |
| Selection process | 8 | Specify the methods used to decide whether a study met the inclusion criteria of the review, including how many reviewers screened each record and each report retrieved, whether they worked independently, and if applicable, details of automation tools used in the process. | 6 |
| Data collection process | 9 | Specify the methods used to collect data from reports, including how many reviewers collected data from each report, whether they worked independently, any processes for obtaining or confirming data from study investigators, and if applicable, details of automation tools used in the process. | 6 |
| Data items | 10a | List and define all outcomes for which data were sought. Specify whether all results that were compatible with each outcome domain in each study were sought (e.g. for all measures, time points, analyses), and if not, the methods used to decide which results to collect. | 6 |
|  | 10b | List and define all other variables for which data were sought (e.g. participant and intervention characteristics, funding sources). Describe any assumptions made about any missing or unclear information. | 6-7 |
| Study risk of bias assessment | 11 | Specify the methods used to assess risk of bias in the included studies, including details of the tool(s) used, how many reviewers assessed each study and whether they worked independently, and if applicable, details of automation tools used in the process. | 7 |
| Effect measures | 12 | Specify for each outcome the effect measure(s) (e.g. risk ratio, mean difference) used in the synthesis or presentation of results. | 7-8 |
| Synthesis methods | 13a | Describe the processes used to decide which studies were eligible for each synthesis (e.g. tabulating the study intervention characteristics and comparing against the planned groups for each synthesis (item #5)). | 5-7 |
|  | 13b | Describe any methods required to prepare the data for presentation or synthesis, such as handling of missing summary statistics, or data conversions. | 6-7 |
|  | 13c | Describe any methods used to tabulate or visually display results of individual studies and syntheses. | 5-7 |
|  | 13d | Describe any methods used to synthesize results and provide a rationale for the choice(s). If meta-analysis was performed, describe the model(s), method(s) to identify the presence and extent of statistical heterogeneity, and software package(s) used. | 7-8 |
|  | 13e | Describe any methods used to explore possible causes of heterogeneity among study results (e.g. subgroup analysis, meta-regression). | 7-8 |
|  | 13f | Describe any sensitivity analyses conducted to assess robustness of the synthesized results. | 8 |
| Reporting bias assessment | 14 | Describe any methods used to assess risk of bias due to missing results in a synthesis (arising from reporting biases). | 7 |
| Certainty assessment | 15 | Describe any methods used to assess certainty (or confidence) in the body of evidence for an outcome. | 7-8 |
| **RESULTS** | | |  |
| Study selection | 16a | Describe the results of the search and selection process, from the number of records identified in the search to the number of studies included in the review, ideally using a flow diagram. | 8, Figure 1 |
|  | 16b | Cite studies that might appear to meet the inclusion criteria, but which were excluded, and explain why they were excluded. | 8, Figure 1 |
| Study characteristics | 17 | Cite each included study and present its characteristics. | 9-13, Table 1 |
| Risk of bias in studies | 18 | Present assessments of risk of bias for each included study. | 14, Supplemental Table 1 |
| Results of individual studies | 19 | For all outcomes, present, for each study: (a) summary statistics for each group (where appropriate) and (b) an effect estimate and its precision (e.g. confidence/credible interval), ideally using structured tables or plots. | 9-28, Supplemental Tables 2-3 |
| Results of syntheses | 20a | For each synthesis, briefly summarise the characteristics and risk of bias among contributing studies. | 9-28, Tables 2-3 |
|  | 20b | Present results of all statistical syntheses conducted. If meta-analysis was done, present for each the summary estimate and its precision (e.g. confidence/credible interval) and measures of statistical heterogeneity. If comparing groups, describe the direction of the effect. | 9-28, Figures 2-5 |
|  | 20c | Present results of all investigations of possible causes of heterogeneity among study results. | 28-30 |
|  | 20d | Present results of all sensitivity analyses conducted to assess the robustness of the synthesized results. | 28-30 |
| Reporting biases | 21 | Present assessments of risk of bias due to missing results (arising from reporting biases) for each synthesis assessed. | 29 |
| Certainty of evidence | 22 | Present assessments of certainty (or confidence) in the body of evidence for each outcome assessed. | 29 |
| **DISCUSSION** | | |  |
| Discussion | 23a | Provide a general interpretation of the results in the context of other evidence. | 31-36 |
|  | 23b | Discuss any limitations of the evidence included in the review. | 35-36 |
|  | 23c | Discuss any limitations of the review processes used. | 35-36 |
|  | 23d | Discuss implications of the results for practice, policy, and future research. | 34-35 |
| **OTHER INFORMATION** | | |  |
| Registration and protocol | 24a | Provide registration information for the review, including register name and registration number, or state that the review was not registered. | 5 |
|  | 24b | Indicate where the review protocol can be accessed, or state that a protocol was not prepared. | 5 |
|  | 24c | Describe and explain any amendments to information provided at registration or in the protocol. | Nil |
| Support | 25 | Describe sources of financial or non-financial support for the review, and the role of the funders or sponsors in the review. | 42 |
| Competing interests | 26 | Declare any competing interests of review authors. | 42 |
| Availability of data, code and other materials | 27 | Report which of the following are publicly available and where they can be found: template data collection forms; data extracted from included studies; data used for all analyses; analytic code; any other materials used in the review. | Supplementary Materials |

*From:*  Page MJ, McKenzie JE, Bossuyt PM, Boutron I, Hoffmann TC, Mulrow CD, et al. The PRISMA 2020 statement: an updated guideline for reporting systematic reviews. BMJ 2021;372:n71. doi: 10.1136/bmj.n71
